# Supplementary material for: Detailed systematic analysis of recruitment strategies in randomised controlled trials in patients with an unscheduled admission to hospital
Source: BMJ Open. 2018 Feb 2;8(2):e018581. doi: 10.1136/bmjopen-2017-018581 (PMC5829602; doi:10.1136/bmjopen-2017-018581)
Supplement: Author's manuscript [file bmjopen-2017-018581.draft_revisions.pdf]

BMJ Open is committed to open peer review. As part of this commitment we make the peer review history of every article we publish publicly available.

When an article is published we post the peer reviewers' comments and the authors' responses online. We also post the versions of the paper that were used during peer review. These are the versions that the peer review comments apply to.

The versions of the paper that follow are the versions that were submitted during the peer review process. They are not the versions of record or the final published versions. They should not be cited or distributed as the published version of this manuscript.

BMJ Open is an open access journal and the full, final, typeset and author-corrected version of record of the manuscript is available on our site with no access controls, subscription charges or pay-per-view fees (http://bmjopen.bmj.com).

If you have any questions on BMJ Open's open peer review process please email editorial.bmjopen@bmj.com

BMJ Open

A detailed systematic analysis of recruitment strategies in randomised controlled trials in patients with an unscheduled admission to hospital

Journal:	*BMJ Open*
Manuscript ID	bmjopen-2017-018581
Article Type:	Research
Date Submitted by the Author:	11-Jul-2017
Complete List of Authors:	Rowlands, Ceri; MRC ConDuCT-II Hub for Trials Methodology Research, School of Social & Community Medicine<br>Rooshenas, Leila; MRC ConDuCT-II Hub for Trials Methodology Research, School of Social & Community Medicine, University of Bristol<br>Fairhurst, Katherine; MRC ConDuCT-II Hub for Trials Methodology Research, School of Social & Community Medicine<br>Rees, Jonathan; University Hospitals Bristol NHS Foundation Trust, Division of Surgery, Head & Neck<br>Gamble, Carrol; University of Liverpool, MRC North West Hub for Trials Methodology Research<br>Blazeby, Jane; University of Bristol, Department of Social Medicine
**Primary Subject Heading**:	Research methods
Secondary Subject Heading:	Evidence based practice, Emergency medicine, Health services research
Keywords:	Clinical trials < THERAPEUTICS, STATISTICS & RESEARCH METHODS, ACCIDENT & EMERGENCY MEDICINE, QUALITATIVE RESEARCH

SCHOLARONE™
Manuscripts

A detailed systematic analysis of recruitment strategies in randomised controlled trials in patients with an unscheduled admission to hospital

C Rowlands[1], L Rooshenas[1], K Fairhurst[1], J Rees[2], C Gamble[3], JM Blazeby[1,2]

1. MRC ConDuCT-II Hub for Trials Methodology Research, School of Social & Community Medicine, University of Bristol, UK
2. Division of Surgery, Head & Neck, University Hospitals Bristol NHS Foundation Trust, Bristol, UK
3. MRC North West Hub for Trials Methodology Research, Institute of Translational Medicine, University of Liverpool, UK

Corresponding author: Leila.Rooshenas@bristol.ac.uk

Word count = 3330 excluding tables, figures and references.

For peer review only

Abstract

Objectives To examine and summarise recruitment details of RCTs undertaken in patients with a UHA to consider how to optimise recruitment in future studies.

Design Studies within the ORRCA database (Online Resource for Recruitment Research in Clinical Trials; www.orrca.org.uk) which reported on recruitment to RCTs involving UHAs in patients >18 years were included. Extracted data included trial clinical details, and the rationale and main findings of the recruitment study.

Studies were categorised according to the design of the recruitment study and the nature of the host trials (real or hypothetical). Data on the rationale for the recruitment study and the methods used to optimise recruitment were collected and summarised.

Results Of 3114 articles populating ORRCA, 39 recruitment studies were eligible, focusing on 68 real and 13 hypothetical host RCTs. Four studies were prospectively planned investigations of recruitment interventions, one of which was a nested RCT. Most recruitment papers were reports of recruitment experiences from one or more 'real' RCTs (n=24) or studies using hypothetical RCTs (n=11). Rationales for conducting recruitment studies included limited time for informed consent (IC) and patients being too unwell to provide IC. Methods to optimise recruitment included providing patients with trial information in the pre-hospital setting, technology to allow recruiters to cover multiple sites, screening logs to uncover recruitment barriers, and verbal rather than written information and consent.

Conclusion There is a paucity of high quality research into recruitment in RCTs involving UHAs with only one nested randomised study evaluating a recruitment intervention. Amongst the remaining studies methods to optimise recruitment focused on how to improve information provision in the pre-hospital setting and use of screening logs. Future research in this setting should focus on the prospective evaluation of the well-developed interventions to optimise recruitment.

Abstract word count = 289 words

For peer review only - http://bmjopen.bmj.com/site/about/guidelines.xhtml

Strengths and limitations of this study

- This review is the first to focus on the complex issue of recruitment to RCTs involving patients undergoing an unscheduled hospital admission
- This review is the first publication to utilise the ORRCA database in generating recruitment research
- The ORRCA database continues to evolve as updates encompass newly published recruitment research. Updates to the database may have generated further UHA research articles since the search for this review.

For peer review only

For peer review only - http://bmjopen.bmj.com/site/about/guidelines.xhtml

Introduction

Recruitment to randomised controlled trials (RCTs) is the biggest obstacle to successful trial conduct.(1) Recruitment may be particularly challenging amongst patients who have an unscheduled hospital admission (UHA). In this situation, the condition of the potential participant and the demanding working environment for clinical teams can complicate the process of identification, screening, and informed consent. Patients are often in pain, unwell, and anxious about the underlying problem. There may also be time limitations due to the urgent need to deliver the clinical treatments under evaluation.(2, 3)

There are a number of existing systematic reviews of methods to optimise recruitment to trials in a variety of clinical contexts and patient populations, including cancer (4-6), primary care, (7) geriatrics (8, 9) and minority community patients (10), or a mix of clinical settings.(11-17) None specifically focus on recruitment of patients undergoing UHA.

Established methods exist for recruiting potential RCT participants who are unwell or unconscious and lack capacity. In these circumstances, permission for enrolment into an RCT may be sought from a surrogate decision maker (SDM) (18), or through deferred consent (also known as 'exception from informed consent'): a process whereby a participant is recruited into the trial in order for urgent treatment to be provided and subsequently asked to provide written consent for ongoing participation once they regain capacity.(19) However, recruitment may be particularly challenging where patients are acutely unwell, but retain capacity to decide on enrolment into an RCT. Reviewing the literature on how to optimise recruitment in this setting may lead to valuable insights, and identify areas where further research is needed.

The aim of this paper was to examine recruitment processes and consider the design of recruitment studies in RCTs involving patients with an unscheduled hospital admission, with a view to considering how to optimise recruitment in this setting.

Methods

Search strategy

Articles were identified through manually screening each entry within the ORRCA recruitment research database (Online Resource for Recruitment research in Clinical triAls;

For peer review only - http://bmjopen.bmj.com/site/about/guidelines.xhtml

http://www.orrca.org.uk/). The ORRCA project was funded by the UK Medical Research Council (MRC) Hubs for Trials Methodology Research Network (HTMRN). It provides a comprehensive online database of published empirical and non- empirical papers about recruitment to clinical research. ORRCA is populated from an extensive systematic search of the Cochrane Library, MEDLINE (Ovid), SCOPUS, ERIC and SCI-EXPANDED and SSCI (via ISI Web of Science). The search strategy employed by ORRCA was based on a Cochrane systematic review of trial recruitment.(16) Further details about the formation of the ORRCA database is reported on their website (http://www.orrca.org.uk/). In this review, a full up-to-date copy of the ORRCA database was obtained in January 2016, and the database was searched in February 2016. At this stage ORRCA contained publications relevant to recruitment published up to 2014.

Study eligibility criteria

Any study held within ORRCA that reported on recruitment to RCTs involving UHAs was eligible for inclusion. UHA was defined as an unscheduled admission to hospital at short notice because of clinical need. This included pre-hospital care, intensive care (ICU) admissions, and accident and emergency (A&E) department attendances. Studies which reported on a mix of patients undergoing scheduled and unscheduled admissions were eligible if the findings for the UHA study population were described separately. Papers that reported recruitment strategies for a mixture of RCTs and other types of research (i.e. non-RCTs) were only eligible for inclusion is the recruitment strategies for RCTs were described separately. RCTs that involved children (age <18 years) or patients with acute psychiatric illness were excluded, as these patients would not normally be subject to the usual recruitment processes due to differences in the consent processes. Systematic reviews of methods to optimise recruitment were scrutinised for relevant primary articles, but not included in the analysis. Abstracts, editorials, and studies of recruitment to non-RCTs were excluded.

Screening and selection process

One author (CR) screened all articles included within the ORRCA database. Duplicate screening was carried out by one other author (KF) on 10% of the database. Papers were assessed at title and abstract level according to the eligibility criteria. Full versions of any papers not definitely excluded at this stage were reviewed for a final decision regarding inclusion. Differences in opinion were resolved by discussion between CR and KF, with the

For peer review only - http://bmjopen.bmj.com/site/about/guidelines.xhtml

aim of reaching agreement for screening decisions on all studies within this sample. Any differences in opinion were referred to another member of the study team (JB) if required. Agreement was reached on all studies during duplicate screening.

Definitions – host RCT

All studies focused on recruitment to one or more specific 'host' RCTs. In this paper, a host RCT refers to the underlying randomised controlled trial, (i.e. addressing a clinical question) in which the recruitment of participants took place. A host RCT could be a pilot or a main trial. Some recruitment papers reported on community consultations in which the views of the public were sought to establish the likelihood of recruitment success or acceptability of a proposed trial. This approach is typically used when the study population may be critically ill at the time of recruitment (and therefore may be unable to provide full, written informed consent).(20) In recognition of this, a clear differentiation was made between studies that focused on recruitment to an existing clinical RCT (a 'real' host RCT) versus potential recruitment to an RCT that did not exist (a 'hypothetical' host RCT), but is proposed to exist in order to estimate its acceptability to potential participants. A 'recruitment study' refers to research into the process of recruiting eligible participants, in the context of one or more real or hypothetical host RCTs.

Definitions - recruitment study design

In order to group similarly designed recruitment studies together and enhance data analysis a new categorisation system for different recruitment study designs was developed (Categories A to D). Consideration was given to the design of the recruitment study and whether a real or hypothetical host RCT was used. The categories are provided in Table 1.

Contacting study authors

If an appropriate recruitment study did not adequately describe the host trial, the study authors were contacted by email to determine whether the host trial met the eligibility criteria. Three attempts, each two weeks apart, were made to contact study authors. If no response was received, then the paper was excluded.

Data Extraction and Synthesis

CR extracted the data using a pre-designed and piloted data extraction form. Data extracted from eligible studies included clinical details of the underlying host RCT, the rationale for

For peer review only - http://bmjopen.bmj.com/site/about/guidelines.xhtml

conducting a recruitment study, a summary of the recruitment study findings, recommendations for improving recruitment, and suggestions for further research. Where specific recruitment interventions had been evaluated, further details regarding the interventions were collected, including the timing of information exchange, informed consent, and randomisation. No statistical analyses were planned, as the review was expected to provide a descriptive analysis of results due to the anticipated heterogeneous nature of recruitment strategies presented. During the process, multiple meetings were undertaken with JB to examine papers and check data extraction processes as required.

Results

Study selection

A total of 3114 articles were identified within the ORRCA database. After initial screening at title and abstract level, 3044 articles were excluded, leaving 70 potentially eligible for which full texts were obtained. A further 31 articles were excluded following full text screening. Duplicate screening did not produce any discrepancies which could not be resolved through discussion. In total therefore, 39 recruitment studies (21-58) which related to 68 real host RCTs and 13 hypothetical host RCTs were included in this review (Figure 1).

The 68 real host trials (around which recruitment was focused) were predominantly multi-centre RCTs (63/68) with large study populations (median = 624 participants, range = 4-58,050), and typically evaluated non-invasive medical interventions (61/68) (Table 2). The apparent predominance of RCTs in neurology is caused by one recruitment study which included data from 32 separate RCTs. With exception to this, the clinical settings of the host RCTs varied, encompassing several medical disciplines.

The majority of recruitment studies were simple descriptive non-randomised studies reporting previous experiences/challenges of recruitment to a host RCT (Category C, n =24). There were 11 that proposed a hypothetical RCT to a study population (Category D, n =11). Only one article used what is considered to be the optimal method for evaluating an intervention - a randomised comparison of a recruitment intervention nested within a host RCT (Category A). Three studies prospectively evaluated recruitment interventions using non-randomised study designs (Category B) (Table 1).

For peer review only - http://bmjopen.bmj.com/site/about/guidelines.xhtml

RCTs of interventions to optimise recruitment nested within one or more host RCTs (Category A)

Only one of the included recruitment studies investigated two recruitment strategies using a randomised design (Category A studies, Table 3). The rationale for this study was the limited time available for recruitment due the acute medical treatment required by patients. (21) Patients randomised to the intervention group received 'advanced notification' of the trial (via fax or phone) designed to offer patients more time to consider trial participation, compared to the control group who only received information once they met with the clinical team. Consent to participate in the host RCT was obtained in 27/50 (54%) and 25/50 (50%) patients in the intervention and control groups respectively (P= 0.69). Although no improvement in overall recruitment rates was demonstrated using advance notification, the provision of early information was demonstrated to be feasible.

Prospectively designed, non-randomised studies of interventions to optimise recruitment to one or more host RCTs (Category B)

The common rationale for this type of recruitment study design (Category B studies, Table 3) was the limitations of time when dealing with patients who required acute treatment. The need for urgent treatment was thought to hinder the ability of the study team to gain informed consent (IC) for trial participation.

The strategy of optimising information provision in the pre-hospital setting used in the one Category A study was mirrored by two Category B studies, which utilised the presence of pre-hospital staff to engage potential trial participants.(22, 24) Recruitment in these studies was reportedly optimised through provision of brief verbal information to participants as they travelled to hospital, and initial verbal consent that sought permission to deliver the emergency trial intervention. Further information was provided and written consent was subsequently obtained when the patients were stabilised, in hospital. One of these studies (24) also provided training to pre-hospital staff to improve their understanding of trial conduct, and devised a simple assessment of capacity to ensure that patients' initial verbal consent was valid.

The remaining prospectively designed (Category B) study focused on using technology to enhance recruitment during an influenza outbreak.(23) As patient numbers would be expected to rise rapidly across a wide geographical area, the study team devised a system

For peer review only - http://bmjopen.bmj.com/site/about/guidelines.xhtml

which provided them with automated, real-time alerts whenever an eligible participant was identified in each hospital. This allowed one centralised study team to cover numerous study sites, enhancing recruitment opportunities. Although none of these studies provided numerical evidence of the effectiveness of their recruitment strategies, all authors concluded that their presented strategies were feasible and acceptable for use in UHA RCTs.

Studies describing recruitment experiences involving one or more host RCTs (Category C)

Rationales for reporting authors' experiences of recruitment were similar to those in prospectively designed studies, including the limited time available for consent (n=13), and recruitment difficulties caused by the clinical condition of the patients (n=18) (Category C studies, Table 4). Some studies were prompted by a host trial encountering recruitment difficulties (n=3). Of the 24 non-randomised studies describing recruitment experiences involving one or more host RCTs, 14 were observational and five were qualitative studies. These studies reported experiences of trial participants and surrogate decision makers (SDMs), or extracted verbatim information from written patient information sheets (PISs). Recommendations for optimising recruitment also mirrored Category A and B studies, highlighting the acceptability of verbal information provision and consent, or deferring consent altogether until an unwell patient is suitably stabilised. Additional benefits were seen in RCTs which used data from screening logs to uncover recruitment barriers, and trials which performed regular site visits.(29-31, 33, 35, 38, 39, 44) A sub section of qualitative studies, although not presenting recommendations for future trial conduct, highlighted their findings that many patients or SDMs who had provided consent to participate in an RCT did not recall much of the information provided to them during the consent process, suggesting that work was needed to improve consent in this setting.(31, 32, 40)

Studies to consider recruitment within proposed hypothetical RCTs (commonly known as community consultations) (Category D)

Eleven studies reported community views about proposed 'hypothetical' RCTs (Category D studies, Table 5). Although the rationales for conducting the studies were similar to studies involving real host RCTs (unwell patients and lack of time for consent), the study designs were varied including questionnaire surveys, interviews, and focus group meetings. Verbal information provision, verbal consent, and recruitment in the pre-hospital setting were identified as helpful recruitment strategies in these studies. However, these articles raised new issues around the appropriateness of using of SDMs when patients are too unwell to

For peer review only - http://bmjopen.bmj.com/site/about/guidelines.xhtml

provide consent for themselves, and raised additional issues around who the SDM should be (next of kin (NOK), or an available physician). Some studies found a preference for the use of SDMs,(50, 52, 56) while others expressed that NOK or physicians should not be used for their specific hypothetical RCTs.(53, 55, 58)

Discussion

This review aimed to examine and summarise studies and methods used to optimise recruitment in RCTs in patients with an unscheduled hospital admission (UHA). It had the purpose of using the information to consider how to optimise recruitment in this challenging clinical setting in future studies. In the ORCCA database of recruitment research, only 39 out of a possible 3114 articles (1.25%) focused on recruitment to RCTs in the UHA setting. Only one of these studies was a randomised comparison of recruitment strategies; the majority of studies consisted of simple study designs describing recruitment experiences. Eleven further studies involved hypothetical RCTs, and whilst of some value, it is uncertain how these types of investigations translate into optimal RCT design. This work therefore highlights the need for development of interventions to optimise recruitment in the UHA setting and prospective evaluation of their effectiveness and acceptability.

Comparison to existing literature

Other authors have examined recruitment in challenging situations. One systematic review focused on recruitment to RCTs in patients with cancer or organ failure and similarly concluded that a lack of high quality evidence hampered recommendations on recruitment.(59) In addition, it highlighted that providing audio-visual information (such as a video explain the RCT) could enhance recruitment. This was not a suggested mechanism for improving recruitment in this review, however it does align with the suggestion that large amounts of written information may not be appropriate in patients with ill-health and is keeping with guidance issued by the NHS Health Research Authority (HRA) on using alternatives to written information (60).

A verbal exchange of information and providing initial verbal consent, rather than labouring unwell patients with written information has been previously suggested as a preferred strategy for trials in the emergency setting(61) and is echoed by the findings in this review. Using verbal consent, however, carries a degree of complexity. Patients who are unwell, may

For peer review only - http://bmjopen.bmj.com/site/about/guidelines.xhtml

have a limited amount of capacity and may not be able to fully engage with a researcher, but nevertheless retain the right to have a role in the decision-making process. One suggested solution to this problem is the use of an independent patient advocate, who may oversee such conversations between trial team members and acutely unwell patients, to verify that appropriate information exchange took place, and to act as an assessor of a patient's willingness to participate in the RCT.(62) This strategy provides one potential solution to a significant obstacle in recruitment of patients undergoing an UHA. However, it may be practically difficult to achieve given that UHAs can occur at any time of the day or night and such trained patient advocates would also need to be available during these times.

Finally, a non-systematic review article focusing on recruitment to emergency medicine research also highlighted a similar problem in a lack of high quality evidence on recruitment. It suggested support for the use of deferred consent (also known as exception from informed consent) which was highlighted in this review and additionally raised the issue that the use of SDMs for consent may be problematic due to the pressures of time and the emotional stress family members will be under while a relative is acutely unwell.(3)

Strengths and weaknesses of this study

This review is the first to systematically focus on recruitment strategies in the UHA setting. With a reported growing number of unscheduled hospital admissions presenting great challenges to modern-day health care provision, the conduct of RCTs in the UHA will inevitably develop as an area of research.

The review may be limited through its reliance on a single search of the ORRCA database, conducted in February 2016, at which point the database contained publications relevant to recruitment published up to 2014. The ORRCA database continues to evolve as updates encompass newly published recruitment research. Updates to the database may have generated further UHA research articles since the search for this review. However, given the small proportion of recruitment papers which were eligible for inclusion (1.25%), it is unlikely that a significant body of research has been omitted during this period.

Unanswered questions and future research

Some articles within this review demonstrated inconsistent conclusions about the value of surrogate decision makers, who these should be, and how these should operate. Future

For peer review only - http://bmjopen.bmj.com/site/about/guidelines.xhtml

research should examine these issues in more depth, in a variety of clinical contexts, focusing on the roles of SDM in different RCTs and to what extent the public it acceptable for an SDM to decide on their behalf for trials involving acutely unwell patients.

It is uncertain how well the results of studies using hypothetical RCTs can be translated to the conduct of real RCTs. There may be some validity in the findings from hypothetical RCTs, as highlighted in this review by the similarity of the results from studies which used real RCTs and hypothetical RCTs. Further work is required to ascertain the extent to which findings from studies which use hypothetical RCTs reliably influence the design and conduct of real RCTs.

As part of this review a classification system was devised in order to group together similarly designed recruitment research studies. Before any such classification system could be used more widely, it would require validation by testing its applicability to at least one further set of recruitment papers, preferably in a different health care setting.

This review highlighted strategies to deal with patients in the pre-hospital setting who required urgent treatment. However, not all patients who are cared for in the pre-hospital setting require treatment immediately. Some may require transport to hospital for further assessment and potential treatment. Further research could explore whether providing early trial information to such patients based on their symptoms or presumed diagnosis, could affect trial recruitment, should treatment be required later. This strategy could prove to be useful in a broader range of unscheduled hospital admissions.

Although some recommendations for optimising recruitment could be drawn from this review, the overall lack of research in this area, particularly amongst high quality, methodologically robust studies, is a limiting factor. Future recruitment studies in this clinical setting should focus on studies with higher methodological rigour, by developing novel interventions to optimise recruitment and prospectively evaluating their effectiveness through an appropriate study design.

Conclusion

For peer review only - http://bmjopen.bmj.com/site/about/guidelines.xhtml

There is a relative paucity of high quality research on strategies to optimise recruitment to RCTs involving unscheduled hospital admissions. Some emerging recommendations include optimising information provision about the trial in the pre-hospital setting to improve recruitment where treatment is required urgently, or using technology to facilitate recruitment across many hospital sites. Screening log data can also provide useful insight to specific barriers to recruitment. Future research in this setting should focus on conducting studies with higher methodological rigour, by developing interventions to optimise recruitment and prospectively evaluating their effectiveness.

Acknowledgements

The following are gratefully acknowledged for their assistance and support with this work: Shaun Treweek, Anna Kearney, Nicola Harman.

Funding

CR is supported by the Medical Research Council (MRC) Hub for Trials Methodology Research Network and the Collaboration and Innovation for Difficult Trials in Invasive procedures (ConDuCT-II) Hub for Trials Methodology Research (MR/K025643/1). The funders had no role in study design, data collection and analysis, decision to publish, or preparation of manuscript.

Contributor statement

CR, LR JR, and JMB designed the study and formulated the protocol. CR and KF performed all data extraction, which was reviewed and critically analysed by CR, LR and JMB. CG facilitated access to the ORRCA database and critically appraised the review. CR produced the first draft of the manuscript and LR and JMB made substantial contributions to the formulation of the final version. All authors read and approved the final manuscript.

Competing interests

No competing interests

Data sharing statement

As this is a review of published literature, there are no additional data available.

References

For peer review only - http://bmjopen.bmj.com/site/about/guidelines.xhtml
For peer review only

1. Tudur Smith C, Hickey H, Clarke M, Blazeby J, Williamson P. The trials methodological research agenda: results from a priority setting exercise. Trials. 2014;15:32-.
2. Schandelmaier S, von Elm E, You JJ, Blumle A, Tomonaga Y, Lamontagne F, et al. Premature Discontinuation of Randomized Trials in Critical and Emergency Care: A Retrospective Cohort Study. Critical care medicine. 2016;44(1):130-7.
3. Cofield SS, Conwit R, Barsan W, Quinn J. Recruitment and retention of patients into emergency medicine clinical trials. Academic emergency medicine : official journal of the Society for Academic Emergency Medicine. 2010;17(10):1104-12.
4. Boland J, Currow DC, Wilcock A, Tieman J, Hussain JA, Pitsillides C, et al. A systematic review of strategies used to increase recruitment of people with cancer or organ failure into clinical trials: implications for palliative care research. Journal of pain and symptom management. 2015;49(4):762-72.e5.
5. Fayter D, McDaid C, Eastwood A. A systematic review highlights threats to validity in studies of barriers to cancer trial participation. Journal of clinical epidemiology. 2007;60(10):990-1001.
6. Mc Daid C, Hodges Z, Fayter D, Stirk L, Eastwood A. Increasing participation of cancer patients in randomised controlled trials: a systematic review. Trials. 2006;7:16.
7. Ngune I, Jiwa M, Dadich A, Lotriet J, Sriram D. Effective recruitment strategies in primary care research: a systematic review. Quality in primary care. 2012;20(2):115-23.
8. Auster J, Janda M. Recruiting older adults to health research studies: A systematic review. Australasian journal on ageing. 2009;28(3):149-51.
9. Provencher V, Mortenson WB, Tanguay-Garneau L, Belanger K, Dagenais M. Challenges and strategies pertaining to recruitment and retention of frail elderly in research studies: a systematic review. Archives of gerontology and geriatrics. 2014;59(1):18-24.
10. Ibrahim S, Sidani S. Strategies to recruit minority persons: a systematic review. Journal of immigrant and minority health / Center for Minority Public Health. 2014;16(5):882-8.
11. Caldwell PH, Hamilton S, Tan A, Craig JC. Strategies for increasing recruitment to randomised controlled trials: systematic review. PLoS medicine. 2010;7(11):e1000368.
12. Fletcher B, Gheorghe A, Moore D, Wilson S, Damery S. Improving the recruitment activity of clinicians in randomised controlled trials: a systematic review. BMJ open. 2012;2(1):e000496.
13. Huynh L, Johns B, Liu SH, Vedula SS, Li T, Puhan MA. Cost-effectiveness of health research study participant recruitment strategies: a systematic review. Clinical trials (London, England). 2014;11(5):576-83.
14. Mapstone J, Elbourne D, Roberts I. Strategies to improve recruitment to research studies. The Cochrane database of systematic reviews. 2007(2):Mr000013.
15. Raftery J, Bryant J, Powell J, Kerr C, Hawker S. Payment to healthcare professionals for patient recruitment to trials: systematic review and qualitative study. Health technology assessment (Winchester, England). 2008;12(10):1-128, iii.

For peer review only

For peer review only - http://bmjopen.bmj.com/site/about/guidelines.xhtml

16. Treweek S, Mitchell E, Pitkethly M, Cook J, Kjeldstrom M, Taskila T, et al. Strategies to improve recruitment to randomised controlled trials. The Cochrane database of systematic reviews. 2010(1):Mr000013.
17. Watson JM, Torgerson DJ. Increasing recruitment to randomised trials: a review of randomised controlled trials. BMC medical research methodology. 2006;6:34.
18. Burns KE, Prats CJ, Maione M, Lanceta M, Zubrinich C, Jeffs L, et al. The Experience of Surrogate Decision Makers on Being Approached for Consent for Patient Participation in Research. A Multicenter Study. Annals of the American Thoracic Society. 2017;14(2):238-45.
19. Whitesides LW, Baren JM, Biros MH, Fleischman RJ, Govindarajan PR, Jones EB, et al. Impact of individual clinical outcomes on trial participants' perspectives on enrollment in emergency research without consent. Clinical trials (London, England). 2017;14(2):180-6.
20. Fordyce CB, Roe MT, Dickert NW. Maximizing value and minimizing barriers: Patient-centered community consultation for research in emergency settings. Clinical trials (London, England). 2017;14(1):88-93.
21. Leira EC, Ahmed A, Lamb DL, Olalde HM, Callison CR, Torner JC, et al. Extending Acute Trials to Remote Populations A Pilot Study During Interhospital Helicopter Transfer. Stroke. 2009;40(3):895-901.
22. Beshansky JR, Sheehan PR, Klima KJ, Hadar N, Vickery EM, Selker HP. A community consultation survey to evaluate support for and success of the IMMEDIATE trial. Clinical Trials. 2014;11(2):178-86.
23. Chow E, Zuberi M, Seto R, Hota S, Fish EN, Morra D. Using real-time alerts for clinical trials Identifying potential study subjects. Applied Clinical Informatics. 2011;2(4):472-80.
24. Shaw L, Price C, McLure S, Howel D, McColl E, Younger P, et al. Paramedic Initiated Lisinopril For Acute Stroke Treatment (PIL-FAST): results from the pilot randomised controlled trial. Emerg Med J. 2014;31(12):994-9.
25. Abramson NS, Safar P. Deferred consent: use in clinical resuscitation research. Brain Resuscitation Clinical Trial II Study Group. Annals of Emergency Medicine. 1990;19(7):781-4.
26. Adeoye O, Pancioli A, Khoury J, Moomaw CJ, Schmit P, Ewing I, et al. Efficiency of Enrollment in a Successful Phase II Acute Stroke Clinical Trial. Journal of Stroke & Cerebrovascular Diseases. 2012;21(8):667-72.
27. Ågård A, Hermerén G, Herlitz J. Patients' experiences of intervention trials on the treatment of myocardial infarction: Is it time to adjust the informed consent procedure to the patient's capacity? Heart. 2001;86(6):632-7.
28. Annane D, Outin H, Fisch C, Bellissant E. The effect of waiving consent on enrollment in a sepsis trial. Intensive Care Medicine. 2004;30(2):321-4.
29. Bellomo R, Trial RRT. Screening and Study Enrolment in the Randomized Evaluation of Normal vs. Augmented Level (RENAL) Replacement Therapy Trial. Blood Purification. 2009;27(2):199-205.
30. Burns KEA, Zubrinich C, Tan WL, Raptis S, Xiong W, Smith O, et al. Research Recruitment Practices and Critically Ill Patients A Multicenter, Cross-Sectional Study (The Consent Study). American Journal of Respiratory and Critical Care Medicine. 2013;187(11):1212-8.

For peer review only - http://bmjopen.bmj.com/site/about/guidelines.xhtml

31. Chlan L, Guttormson J, Tracy MF, Bremer KL. STRATEGIES FOR OVERCOMING SITE AND RECRUITMENT CHALLENGES IN RESEARCH STUDIES BASED IN INTENSIVE CARE UNITS. American Journal of Critical Care. 2009;18(5):410-7.
32. Collins JF. Data and safety monitoring board issues raised in the VA Status Epilepticus Study. Controlled Clinical Trials. 2003;24(1):71-7.
33. Cook D, Arabi Y, Ferguson ND, Heels-Ansdell D, Freitag A, McDonald E, et al. Physicians declining patient enrollment in a critical care trial: a case study in thromboprophylaxis. Intensive Care Medicine. 2013;39(12):2115-25.
34. Costescu DJW, Cullimore AJ. Lessons learned from a resident-led clinical trial in obstetrics. Clinical Trials. 2013;10(4):612-6.
35. Crowley ST, Chertow GM, Vitale J, O'Connor T, Zhang JN, Schein RMH, et al. Lessons for successful study enrollment from the Veterans Affairs/National Institutes of Health Acute Renal Failure Trial Network Study. Clinical Journal of the American Society of Nephrology. 2008;3(4):955-61.
36. Elkins JS, Khatabi T, Fung L, Rootenberg J, Johnston SC. Recruiting subjects for acute stroke trials: a meta-analysis. Stroke [Internet]. 2006; 37(1):[123-8 pp.]. Available from: http://onlinelibrary.wiley.com/o/cochrane/clcmr/articles/CMR-10639/frame.html
http://stroke.ahajournals.org/content/37/1/123.full.pdf.
37. Flaherty ML, Karlawish J, Khoury JC, Kleindorfer D, Woo D, Broderick JP. How important is surrogate consent for stroke research? Neurology. 2008;71(20):1566-71.
38. Foster D, Cook D, Granton J, Steinberg M, Marshall J. Use of a screen log to audit patient recruitment into multiple randomized trials in the intensive care unit. Canadian Critical Care Trials Group. Critical care medicine [Internet]. 2000; 28(3):[867-71 pp.]. Available from: http://onlinelibrary.wiley.com/o/cochrane/clcmr/articles/CMR-11625/frame.html.
39. Glassberg AE, Luce JM, Matthay MA. Reasons for nonenrollment in a clinical trial of acute lung injury. Chest [Internet]. 2008; 134(4):[719-23 pp.]. Available from: http://onlinelibrary.wiley.com/o/cochrane/clcmr/articles/CMR-12450/frame.html
http://journal.publications.chestnet.org/data/Journals/CHEST/22076/zcb01008000719.pdf.
40. Kendall B, Stadeli R, Schegg B, Olbrich M, Chen E, Harmelin-Kadouri R, et al. Clinical Trial Educator program - a novel approach to accelerate enrollment in a phase III International Acute Coronary Syndrome Trial. Clinical Trials. 2012;9(3):358-66.
41. Kenyon S, Dixon-Woods M, Jackson CJ, Windridge K, Pitchforth E. Participating in a trial in a critical situation: a qualitative study in pregnancy. Quality & Safety in Health Care. 2006;15(2):98-101.
42. Roozenbeek B, Maas AIR, Marmarou A, Butcher I, Lingsma HF, Lu J, et al. The influence of enrollment criteria on recruitment and outcome distribution in traumatic brain injury studies: results from the impact study. Journal of Neurotrauma [Internet]. 2009; 26(7):[1069-75 pp.]. Available from: http://onlinelibrary.wiley.com/o/cochrane/clcmr/articles/CMR-14708/frame.html
http://online.liebertpub.com/doi/pdfplus/10.1089/neu.2008.0569.
43. Schats R, Brilstra EH, Rinkel GJ, Algra A, Gijn J. Informed consent in trials for neurological emergencies: the example of subarachnoid haemorrhage. Journal of Neurology, Neurosurgery, and Psychiatry [Internet]. 2003; 74(7):[988-91 pp.].

For peer review only - http://bmjopen.bmj.com/site/about/guidelines.xhtml

Available from: http://onlinelibrary.wiley.com/o/cochrane/clcmr/articles/CMR-12831/frame.html
http://www.ncbi.nlm.nih.gov/pmc/articles/PMC1738516/pdf/v074p00988.pdf.
44. Smith OM, McDonald E, Zytaruk N, Foster D, Matte A, Clarke F, et al. Rates and determinants of informed consent: A case study of an international thromboprophylaxis trial. Journal of Critical Care. 2013;28(1):28-39.
45. Smyth RM, Jacoby A, Elbourne D. Deciding to join a perinatal randomised controlled trial: experiences and views of pregnant women enroled in the Magpie Trial. Midwifery. 2012;28(4):E478-85.
46. Williams BF, French JK, White HD. Informed consent during the clinical emergency of acute myocardial infarction (HERO-2 consent substudy): A prospective observational study. Lancet. 2003;361(9361):918-22.
47. Yamal JM, Robertson CS, Rubin ML, Benoit JS, Hannay HJ, Tilley BC. Enrollment of racially/ethnically diverse participants in traumatic brain injury trials: Effect of availability of exception from informed consent. Clinical Trials. 2014;11(2):187-94.
48. Yuval R, Halon DA, Merdler A, Khader N, Karkabi B, Uziel K, et al. Patient comprehension and reaction to participating in a double-blind randomized clinical trial (ISIS-4) in acute myocardial infarction. Archives of Internal Medicine. 2000;160(8):1142-6.
49. Abboud PA, Heard K, Al-Marshad AA, Lowenstein SR. What determines whether patients are willing to participate in resuscitation studies requiring exception from informed consent? Journal of Medical Ethics [Internet]. 2006; 32(8):[468-72 pp.]. Available from: http://onlinelibrary.wiley.com/o/cochrane/clcmr/articles/CMR-10612/frame.html.
50. Ali K, Roffe C, Crome P. What patients want: consumer involvement in the design of a randomized controlled trial of routine oxygen supplementation after acute stroke. Stroke [Internet]. 2006; 37(3):[865-71 pp.]. Available from: http://onlinelibrary.wiley.com/o/cochrane/clcmr/articles/CMR-10638/frame.html
http://stroke.ahajournals.org/content/37/3/865.full.pdf.
51. Bulger EM, Schmidt TA, Cook AJ, Brasel KJ, Griffiths DE, Kudenchuk PJ, et al. The Random Dialing Survey as a tool for community consultation for research involving the emergency medicine exception from informed consent. Annals of Emergency Medicine [Internet]. 2009; 53(3):[341-50 pp.]. Available from: http://onlinelibrary.wiley.com/o/cochrane/clcmr/articles/CMR-14468/frame.html
http://ac.els-cdn.com/S0196064408015126/1-s2.0-S0196064408015126-main.pdf?_tid=3127512a-62ab-11e5-948a-00000aab0f27&acdnat=1443092490_2b79f56df4905bd0b2acd045224491cf.
52. Clark DJ, Kolias AG, Corteen EA, Ingham SC, Piercy J, Crick SJ, et al. Community consultation in emergency neurotrauma research: results from a pre-protocol survey. Acta Neurochirurgica. 2013;155(7):1329-34; discussion 34.
53. Giudice AD, Plaum J, Maloney E, Kasner SE, Le Roux PD, Baren JM. Who will consent to emergency treatment trials for subarachnoid hemorrhage? Academic Emergency Medicine. 2009;16(4):309-15.
54. Goldstein JN, Espinola JA, Fisher J, Pallin DJ, Camargo Jr CA. Public opinion of a stroke clinical trial using exception from informed consent. International Journal of Emergency Medicine. 2010;3(4):385-9.

For peer review only - http://bmjopen.bmj.com/site/about/guidelines.xhtml

55. Newman JT, Smart A, Reese TR, Williams A, Moss M. Surrogate and patient discrepancy regarding consent for critical care research. Critical care medicine. 2012;40(9):2590-4.
56. Scotton WJ, Kolias AG, Ban VS, Crick SJ, Sinha R, Gardner A, et al. Community consultation in emergency neurosurgical research: lessons from a proposed trial for patients with chronic subdural haematomas. British Journal of Neurosurgery. 2013;27(5):590-4.
57. Sims CA, Isserman JA, Holena D, Sundaram LM, Tolstoy N, Greer S, et al. Exception from informed consent for emergency research: Consulting the trauma community. Journal of Trauma and Acute Care Surgery. 2013;74(1):157-66.
58. Snowdon C, Elbourne D, Forsey M, Alfirevic Z. Views of emergency research (VERA): A qualitative study of women and their partners' views of recruitment to trials in severe postpartum haemorrhage. Midwifery. 2012;28(6):800-8.
59. Boland J, Currow DC, Wilcock A, Tieman J, Hussain JA, Pitsillides C, et al. A systematic review of strategies used to increase recruitment of people with cancer or organ failure into clinical trials: implications for palliative care research. Journal of pain and symptom management. 2015;49(4):762-72 e5.
60. Authority NHR. Applying a proportionate approach to the process of seeking consent - HRA Guidance. 2017.
61. Roberts I, Prieto-Merino D, Shakur H, Chalmers I, Nicholl J. Effect of consent rituals on mortality in emergency care research. Lancet. 2011;377(9771):1071-2.
62. Sahan KM, Channon KM, Choudhury RP, Kharbanda RK, Lee R, Sheehan M. Refining the Enrolment Process in Emergency Medicine Research. Eur J Cardiovasc Med. 2016;4(1):506-10.

For peer review only

For peer review only - http://bmjopen.bmj.com/site/about/guidelines.xhtml

Figure 1: Study selection PRISMA flow diagram

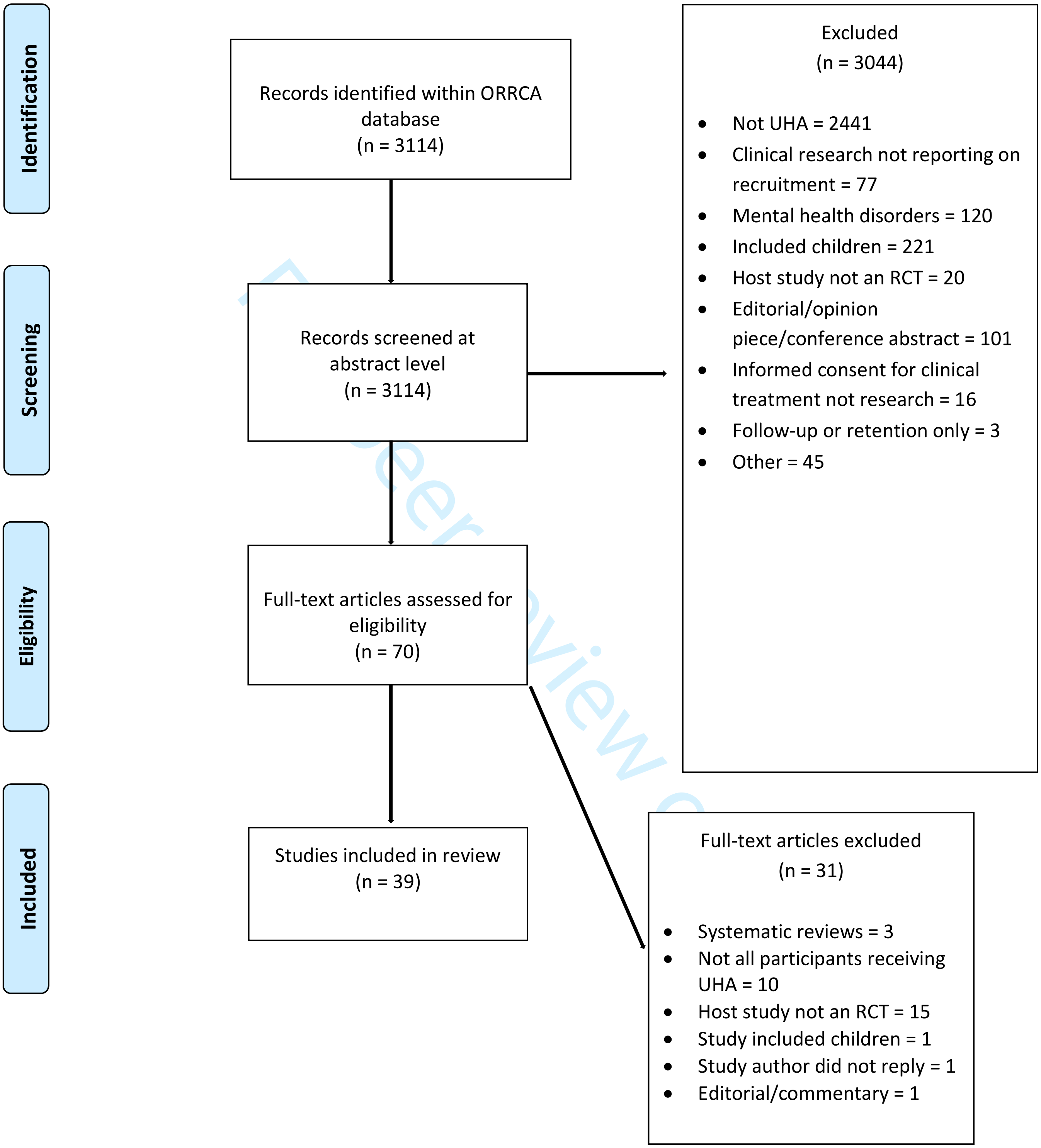

*RCT = randomised controlled trial, UHA = unscheduled hospital admission, ORRCA = Online Resource for Recruitment Research in Clinical Trials

Table 1: Number of included studies, host RCTs and hypothetical RCTs categorised according to the new recruitment study types (A to E)

Category	Recruitment study design	Recruitment studies (n=39)	Real host RCTs (n=68)	Hypothetical host RCTs (n=13)
A	Randomised controlled trials (RCTs) of interventions to optimise recruitment nested within one or more host RCTs	1	1	N/A
B	Prospectively designed, non-randomised studies of interventions to optimise recruitment to one or more host RCTs	3	3	N/A
C	Studies describing recruitment experiences involving one or more host RCTs	24	64	N/A
D	Studies to consider recruitment within proposed hypothetical RCTs (commonly known as community consultations)	11	N/A	13

*RCT = randomised controlled trial, N/A = not applicable – category does not apply to type of host RCT

For peer review only

Table 2: Summary characteristics of the host RCTs and hypothetical host RCTs in this review

Trial characteristic	Real RCTs (n = 68)	Hypothetical RCTs (n = 13)	Total (n=81)
**Clinical Setting**			
Neurology	39*	4	43
Cardiology	8	4	12
Obstetrics	3	1	4
Infection	3	0	3
Trauma	6	3	9
Critical care	9	1	10
**Type of interventions**			
Invasive / non-invasive	5 / 61	4 / 9	9 / 70
Unknown	2	0	2
**Trial design**			
Main RCT/ pilot RCT	65/3	n/a	65 / 3
Two groups / more than two groups	63 / 5	11 / 2	74 / 7
**Number of centres****			
Single centre / multi-centre / unknown	3 / 63 / 2	0 / 4 / 9	3 / 67 / 11
<20 centres	18		
20 -100 centres	33		
>100 centres	15		
Median number of centres (range)	45 (1 - 818)		
**Number of participants****			
<500 participants	22		
500 -1500 participants	32		
>1500 participants	14		
Median number of participants (range)	624 (4 - 58,050)		

*One recruitment paper included data from 32 Stroke host RCTs. RCT = randomised controlled trial

** Hypothetical RCT data did not include information beyond single or multicentre, nor the suggested no. of participants

For peer review

Table 3: Rationale and types of recruitment interventions reported in Category A or B studies (i.e. prospectively nested within RCTs)

Author and year	Rationale(s)	Descriptive summary of recruitment intervention(s)	Classification of recruitment intervention	Timing of information provision regarding host RCT	Timing of informed consent for host RCT	Timing of randomisation for host RCT
**Prospectively designed RCTs of interventions to optimise recruitment nested within one or more host RCTs (Category A studies)**						
Leira** 2009	Limited time for IC in host RCT due to the clinical condition requiring urgent treatment.	Intervention group: 'Advance notification' about host RCT using phone and fax prior to meeting pre-hospital team<br>Control group: No information prior to meeting pre-hospital team	Advance notification using phone and fax	Intervention group: 'Advance notification' prior to face-to face meeting with pre-hospital team<br>Control group: During face-to-face meeting with pre-hospital team	Intervention group: During face-to-face meeting with pre-hospital team<br>Control group: During face-to-face meeting with pre-hospital team	Intervention group: Whilst patient in pre-hospital setting.<br>Control group: Whilst patient in pre-hospital setting.
**Prospectively designed, non-randomised studies of interventions to optimise recruitment to one or more host RCTs (Category B studies)**						
Chow 2011	Recruitment team would be overwhelmed by covering multiple sites over a wide geographical area.	An automated service notified recruitment team in real time when a potentially eligible participant was identified across multiple centres	Automated service to facilitate real time notifications to recruitment team covering multiple sites	During face-to-face meeting with recruitment staff (once eligibility confirmed)	During face-to-face meeting with recruitment team	Whilst patient in hospital.
Shaw 2014	Limited time for IC in host RCT due to the clinical condition requiring urgent treatment.<br>Pre-hospital staff have limited experience of RCTs	Standardised verbal information was provided by pre-hospital staff before a simple capacity assessment, verbal consent and delivery of emergency treatment<br>Host RCT procedures were tailored towards pre-hospital staff, who also attended a one-day training event	Optimising information provision in pre-hospital setting.<br>Allowing verbal consent from patient or SDM.<br>Training of pre-hospital staff	Simple verbal information provided in pre-hospital setting.<br>Further full information provided in hospital	Initial verbal consent given in pre-hospital setting.<br>Written informed consent gained in hospital.	Whilst patient in pre-hospital setting.
Beshansky 2014	Limited time for IC in host RCT due to the clinical condition requiring urgent treatment.	Standardised verbal information was provided by pre-hospital staff before a simple capacity assessment, verbal consent and delivery of emergency treatment	Optimising information provision in pre-hospital setting.<br>Allowing verbal consent from patient.	Simple verbal information provided in pre-hospital setting.<br>Further full information provided in hospital	Initial verbal consent in pre-hospital setting.<br>Written informed consent in hospital.	Whilst patient in pre-hospital setting.

RCT = randomised controlled trial, IC= informed consent, SDM = surrogate decision maker
** Leira et al – patients were randomised to the *recruitment* intervention or control group prior to the recruitment team seeing the patient, and without consent from the participant.

For peer review only

Table 4: Frequency of rationales, study designs and recommendations from non-randomised studies describing recruitment experiences involving one or more host RCTs (i.e. Category C studies).

Recruitment study characteristic	Description	Frequency in Category C recruitment studies (n=24)**
**Rationale**	Patients too unwell to provide IC	18
	Limited time for IC in host RCT due to the clinical condition requiring urgent treatment	13
	Host RCT not meeting recruitment targets (at one or more sites) or terminated due to poor recruitment	3
	To better understand the impact of altering eligibility criteria on recruitment	2
	To better understand the impact of availability of SDMs on recruitment	2
	To better understand the recruitment process in a host RCT	1
	To better understand clinicians reasons for refusing patient participation in host RCT	1
**Recruitment study design**	Observational study of recruitment	14
	Qualitative studies of host participants/SDMs or PIS	5
	Survey of host RCT participants	2
	Survey of clinical staff involved in host RCT	1
	Simulation study evaluating the effect of altering eligibility criteria in multiple host RCTs	1
	Meta-analysis of recruitment data in host RCTs	1
**Recommendations for optimising recruitment in future RCTs or areas for further research into recruitment*** **	To provide RCT information verbally and allow a verbal consent process	10
	To use a screening log can to provide insight into recruitment difficulties	8
	Patients or SDM were unable to recall key RCT information after providing IC***	5
	To use a 'waiver of consent'/ 'deferred consent'/ 'EFIC'	4
	To perform regular site visits	3
	To use a broad eligibility criteria /broad therapeutic window	2
	To use SDMs	2
	Novel methods for obtaining IC are required***	2
	To replace poorly recruiting centres	1
	To approach more eligible patients	1
	To survey staff involved with host RCT to provide insight into recruitment difficulties	1

RCT = randomised controlled trial, IC= informed consent, EFIC = exception from informed consent, SDM = surrogate decision maker, PIS= patient information sheet.
**each study may appear more than once in the relevant characteristics section (e.g. if it described more >1 rationale, or produced >1 finding/recommendation)
*** items for further research and not recommendations for optimising recruitment

For peer review only - http://bmjopen.bmj.com/site/about/guidelines.xhtml

Table 5: Frequency of rationales, study designs, main findings and recommendations from Non-randomised studies designed study to consider recruitment within proposed hypothetical RCTs (i.e. Category D studies, commonly known as community consultations).

Recruitment study characteristic	Description	Frequency in Category D recruitment studies (n=11)**
**Rationale**	Patients too unwell to provide IC	9
	Limited time for IC in host RCT due to the clinical condition requiring urgent treatment	6
	To explore the accuracy of decisions made by NOK when acting as SDM	1
**Recruitment study design**	Questionnaire survey	5
	Face-to-face interview	4
	Telephone survey	1
	Focus group meetings	1
**Recommendations for optimising recruitment in future RCTs or main findings*** **	To use a physician as a SDM	4
	To use NOK as a SDM	4
	To use EFIC	3
	**Not** to use NOK as a SDM	2
	To provide RCT information verbally and allow a verbal consent process	1
	To allow recruitment in pre-hospital setting	1
	To perform community consultations to estimate host RCT recruitment rates***	1
	**Not** to use a physician as a SDM	1
	**Not** to use EFIC	1
	To perform community consultations to aid selection of relevant study outcomes***	1

*RCT = randomised controlled trial, EFIC = exception from informed consent, NOK = next of kin, SDM = surrogate decision maker

**each study may appear more than once in the relevant characteristics section (e.g. if it described more >1 rationale, or produced >1 finding/recommendation)

*** items reported as main findings, but not recommendations for optimising recruitment

For peer review only

BMJ Open

A detailed systematic analysis of recruitment strategies in randomised controlled trials in patients with an unscheduled admission to hospital

Journal:	*BMJ Open*
Manuscript ID	bmjopen-2017-018581.R1
Article Type:	Research
Date Submitted by the Author:	19-Sep-2017
Complete List of Authors:	Rowlands, Ceri; MRC ConDuCT-II Hub for Trials Methodology Research, School of Social & Community Medicine<br>Rooshenas, Leila; MRC ConDuCT-II Hub for Trials Methodology Research, School of Social & Community Medicine, University of Bristol<br>Fairhurst, Katherine; MRC ConDuCT-II Hub for Trials Methodology Research, School of Social & Community Medicine<br>Rees, Jonathan; University Hospitals Bristol NHS Foundation Trust, Division of Surgery, Head & Neck<br>Gamble, Carrol; University of Liverpool, MRC North West Hub for Trials Methodology Research<br>Blazeby, Jane; University of Bristol, Department of Social Medicine
**Primary Subject Heading**:	Research methods
Secondary Subject Heading:	Evidence based practice, Emergency medicine, Health services research
Keywords:	Clinical trials < THERAPEUTICS, STATISTICS & RESEARCH METHODS, ACCIDENT & EMERGENCY MEDICINE, QUALITATIVE RESEARCH

SCHOLARONE™
Manuscripts

A detailed systematic analysis of recruitment strategies in randomised controlled trials in patients with an unscheduled admission to hospital

C Rowlands[1], L Rooshenas[1], K Fairhurst[1], J Rees[2], C Gamble[3], JM Blazeby[1,2]

1. MRC ConDuCT-II Hub for Trials Methodology Research, School of Social & Community Medicine, University of Bristol, UK
2. Division of Surgery, Head & Neck, University Hospitals Bristol NHS Foundation Trust, Bristol, UK
3. MRC North West Hub for Trials Methodology Research, Institute of Translational Medicine, University of Liverpool, UK

Corresponding author: Leila.Rooshenas@bristol.ac.uk

Word count = 3330 excluding tables, figures and references.

For peer review only

For peer review only - http://bmjopen.bmj.com/site/about/guidelines.xhtml

Abstract

Objectives To examine the design and findings of recruitment studies in RCTs involving patients with an unscheduled admission to hospital (UHA), to consider how to optimise recruitment in future RCTs of this nature.

Design Studies within the ORRCA database (Online Resource for Recruitment Research in Clinical Trials; www.orrca.org.uk) which reported on recruitment to RCTs involving UHAs in patients >18 years were included. Extracted data included trial clinical details, and the rationale and main findings of the recruitment study.

Studies were categorised according to the design of the recruitment study and the nature of the host trials (real or hypothetical). Data on the rationale for the recruitment study and the methods used to optimise recruitment were collected and summarised.

Results Of 3114 articles populating ORRCA, 39 recruitment studies were eligible, focusing on 68 real and 13 hypothetical host RCTs. Four studies were prospectively planned investigations of recruitment interventions, one of which was a nested RCT. Most recruitment papers were reports of recruitment experiences from one or more 'real' RCTs (n=24) or studies using hypothetical RCTs (n=11). Rationales for conducting recruitment studies included limited time for informed consent (IC) and patients being too unwell to provide IC. Methods to optimise recruitment included providing patients with trial information in the pre-hospital setting, technology to allow recruiters to cover multiple sites, screening logs to uncover recruitment barriers, and verbal rather than written information and consent.

Conclusion There is a paucity of high quality research into recruitment in RCTs involving UHAs with only one nested randomised study evaluating a recruitment intervention. Amongst the remaining studies methods to optimise recruitment focused on how to improve information provision in the pre-hospital setting and use of screening logs. Future research in this setting should focus on the prospective evaluation of the well-developed interventions to optimise recruitment.

Abstract word count = 297 words

For peer review only - http://bmjopen.bmj.com/site/about/guidelines.xhtml

Strengths and limitations of this study

- This review is the first to focus on the complex issue of recruitment to RCTs involving patients undergoing an unscheduled hospital admission
- This review is the first publication to utilise the ORRCA database in generating recruitment research
- The ORRCA database continues to evolve as updates encompass newly published recruitment research. Updates to the database may have generated further UHA research articles since the completion of this review.

For peer review only

For peer review only - http://bmjopen.bmj.com/site/about/guidelines.xhtml

Introduction

Recruitment to randomised controlled trials (RCTs) is the biggest obstacle to successful trial conduct.(1) Recruitment may be particularly challenging amongst patients who have an unscheduled hospital admission (UHA). In this situation, the condition of the potential participant and the demanding working environment for clinical teams can complicate the process of identification, screening, and informed consent. Patients are often in pain, unwell, and anxious about the underlying problem. There may also be time limitations due to the urgent need to deliver the clinical treatments under evaluation.(2, 3)

There are a number of existing systematic reviews of methods to optimise recruitment to trials in a variety of clinical contexts and patient populations, including cancer (4-6), primary care, (7) geriatrics (8, 9) and minority community patients (10), or a mix of clinical settings.(11-17) None specifically focus on recruitment of patients undergoing UHA.

Established methods exist for recruiting potential RCT participants who are unwell or unconscious and lack capacity. In these circumstances, permission for enrolment into an RCT may be sought from a surrogate decision maker (SDM) (18), or through deferred consent (also known as 'exception from informed consent'): a process whereby a participant is recruited into the trial in order for urgent treatment to be provided and subsequently asked to provide written consent for ongoing participation once they regain capacity.(19) However, recruitment may be particularly challenging where patients are acutely unwell, but retain capacity to decide on enrolment into an RCT. Reviewing the literature on how to optimise recruitment in this setting may lead to valuable insights, and identify areas where further research is needed.

The aim of this paper was to examine the design and findings of recruitment studies in RCTs involving patients with an unscheduled admission to hospital (UHA), to consider how to optimise recruitment in future RCTs of this nature..

Methods

Search strategy

Articles were identified through manually screening each entry within the ORRCA recruitment research database (Online Resource for Recruitment research in Clinical triAls;

For peer review only - http://bmjopen.bmj.com/site/about/guidelines.xhtml

http://www.orrca.org.uk/). The ORRCA project was funded by the UK Medical Research Council (MRC) Hubs for Trials Methodology Research Network (HTMRN). It provides a comprehensive online database of published empirical and non- empirical papers about recruitment to clinical research. ORRCA is populated from an extensive systematic search of the Cochrane Library, MEDLINE (Ovid), SCOPUS, ERIC and SCI-EXPANDED and SSCI (via ISI Web of Science). The search strategy employed by ORRCA was based on a Cochrane systematic review of trial recruitment.(16) Further details about the formation of the ORRCA database is reported on their website (http://www.orrca.org.uk/). In this review, a full up-to-date copy of the ORRCA database was obtained in January 2016, and the database was searched in February 2016. At this stage ORRCA contained publications relevant to recruitment published up to and including the end of December 2014. Publications from 2015 onwards were not available due to ongoing work in processing articles in the ORRCA database.

Study eligibility criteria

Any study held within ORRCA that reported on recruitment to RCTs involving UHAs was eligible for inclusion. UHA was defined as an unscheduled admission to hospital at short notice because of clinical need. This included pre-hospital care, intensive care (ICU) admissions, and accident and emergency (A&E) department attendances. Studies which reported on a mix of patients undergoing scheduled and unscheduled admissions were eligible if the findings for the UHA study population were described separately. Papers that reported recruitment strategies for a mixture of RCTs and other types of research (i.e. non-RCTs) were only eligible for inclusion if the recruitment strategies for RCTs were described separately. RCTs that involved children (age <18 years) or patients with acute psychiatric illness were excluded, as these patients would not normally be subject to the usual recruitment processes due to differences in the consent processes. Systematic reviews of methods to optimise recruitment were scrutinised for relevant primary articles, but not included in the analysis. Editorials, and studies of recruitment to non-RCTs were excluded. Abstracts were also excluded because these rarely included the necessary contextual information and data needed to make a meaningful contribution to the dataset for this study.

For peer review only - http://bmjopen.bmj.com/site/about/guidelines.xhtml

Screening and selection process

One author (CR) screened all articles included within the ORRCA database. Duplicate screening was carried out by one other author (KF) on 10% of the database. KF was blinded to the original screening decisions. Papers were assessed at title and abstract level according to the eligibility criteria. Differences in opinion were resolved by discussion between CR and KF, and any remaining differences in opinion were referred to another member of the study team (JMB) if required. The aim was to reach agreement for screening decisions on all studies within this sample. Agreement was reached on 271/300 articles screened. Of the 29 discrepancies raised, 22 were resolved following discussion between CR and KF. The seven remaining papers were discussed with a third author (JMB), which resulted in two of these papers being included and five being excluded. No paper which was suggested to be included by the second reviewer was eventually included in the review.

We calculated a kappa statistic for the double screened articles above. Ten per cent of articles were double screened with a Kappa = 0.677 (SE= 0.048, p <0.001) suggesting "good" agreement. As such, the remaining 90% of articles in the ORRCA database were screened by a single screener (CR). The third arbiter (JMB) involved in screening the 10% sample was also consulted for screening the remaining 90% of articles in the ORRCA database, in instances where the single screener (CR) was uncertain about an article's eligibility.

Definitions – host RCT

All studies focused on recruitment to one or more specific 'host' RCTs. In this paper, a host RCT refers to the underlying randomised controlled trial, (i.e. addressing a clinical question) in which the recruitment of participants took place. A host RCT could be a pilot or a main trial. Some recruitment papers reported on community consultations in which the views of the public were sought to establish the likelihood of recruitment success or acceptability of a proposed trial. This approach is typically used when the study population may be critically ill at the time of recruitment (and therefore may be unable to provide full, written informed consent).(20) In recognition of this, a clear differentiation was made between studies that focused on recruitment to an existing clinical RCT (a 'real' host RCT) versus potential recruitment to an RCT that did not exist (a 'hypothetical' host RCT), but is proposed to exist in order to estimate its acceptability to potential participants. A 'recruitment study' refers to research into the process of recruiting eligible participants, in the context of one or more real or hypothetical host RCTs.

For peer review only - http://bmjopen.bmj.com/site/about/guidelines.xhtml

Definitions - recruitment study design

In order to group similarly designed recruitment studies together and enhance data analysis a new categorisation system for different recruitment study designs was developed (Categories A to D). Consideration was given to the design of the recruitment study and whether a real or hypothetical host RCT was used. The categories are provided in Table 1.

Contacting study authors

If an appropriate recruitment study did not adequately describe the host trial, the study authors were contacted by email to determine whether the host trial met the eligibility criteria. Three attempts, each two weeks apart, were made to contact study authors. If no response was received, then the paper was excluded.

Data Extraction and Synthesis

CR extracted the data using a pre-designed and piloted data extraction form. Data extracted from eligible studies included clinical details of the underlying host RCT, the rationale for conducting a recruitment study, a summary of the recruitment study findings, recommendations for improving recruitment, and suggestions for further research. Where specific recruitment interventions had been evaluated, further details regarding the interventions were collected, including the timing of information exchange, informed consent, and randomisation. No statistical analyses were planned, as the review was expected to provide a descriptive analysis of results due to the anticipated heterogeneous nature of recruitment strategies presented. During the process, multiple meetings were undertaken with JB to examine papers and check data extraction processes as required.

Results

Study selection

A total of 3114 articles were identified within the ORRCA database. After initial screening at title and abstract level, 3044 articles were excluded, leaving 70 potentially eligible for which full texts were obtained. A further 31 articles were excluded following full text screening. Duplicate screening did not produce any discrepancies which could not be resolved through discussion. In total, 39 recruitment studies (21-58) were identified which reported results from 68 real host RCTs and 13 hypothetical host RCTs and were included in this review

For peer review only - http://bmjopen.bmj.com/site/about/guidelines.xhtml

Table 1: Number of included studies, host RCTs and hypothetical RCTs categorised according to the new recruitment study types (A to D)

Category	Recruitment study design	Recruitment studies (n=39)	Real host RCTs (n=68)	Hypothetical host RCTs (n=13)
**A**	Randomised controlled trials (RCTs) of interventions to optimise recruitment nested within one or more host RCTs	1	1	N/A

For peer review only

For peer review only - http://bmjopen.bmj.com/site/about/guidelines.xhtml

**B**	Prospectively designed, non-randomised studies of interventions to optimise recruitment to one or more host RCTs	3	3	N/A
**C**	Studies describing recruitment experiences involving one or more host RCTs	24	64*	N/A
**D**	Studies to consider recruitment within proposed hypothetical RCTs (commonly known as community consultations)	11	N/A	13**

RCT = randomised controlled trial, N/A = not applicable – category does not apply to type of host RCT

*The 24 recruitment studies reported data from 64 real host RCTs i.e. a number of recruitment studies reported data from more than one real host RCT

**The 11 recruitment studies reported data from 13 hypothetical studies i.e. two recruitment studies reported data from more than one hypothetical host RCT

For peer review only - http://bmjopen.bmj.com/site/about/guidelines.xhtml

(Figure 1). A number of recruitment studies described results obtained from more than one real or hypothetical RCT.

The 68 real host trials (around which recruitment was focused) were predominantly multi-centre RCTs (63/68) with large study populations (median = 624 participants, range = 4-58,050), and typically evaluated non-invasive medical interventions (61/68) (Table 2). The apparent predominance of RCTs in neurology is caused by one recruitment study which included data from 32 separate RCTs. With exception to this, the clinical settings of the host RCTs varied, encompassing several medical disciplines.

The majority of recruitment studies were simple descriptive non-randomised studies reporting previous experiences/challenges of recruitment to a host RCT (Category C, n =24). There were 11 that proposed a hypothetical RCT to a study population (Category D, n =11). Only one article used what is considered to be the optimal method for evaluating an intervention - a randomised comparison of a recruitment intervention nested within a host RCT (Category A). Three studies prospectively evaluated recruitment interventions using non-randomised study designs (Category B) (Table 1).

RCTs of interventions to optimise recruitment nested within one or more host RCTs (Category A)

Only one of the included recruitment studies investigated two recruitment strategies using a randomised design (Category A studies, Table 3). The rationale for this study was the limited time available for recruitment due the acute medical treatment required by patients. (21) Patients randomised to the intervention group received 'advanced notification' of the trial (via fax or phone) designed to offer patients more time to consider trial participation, compared to the control group who only received information once they met with the clinical team. Consent to participate in the host RCT was obtained in 27/50 (54%) and 25/50 (50%) patients in the intervention and control groups respectively (*P*= 0.69). Although no improvement in overall recruitment rates was demonstrated using advance notification, the provision of early information was demonstrated to be feasible.

For peer review only - http://bmjopen.bmj.com/site/about/guidelines.xhtml

Table 2: Summary characteristics of the host RCTs and hypothetical host RCTs in this review

Trial characteristic	Real RCTs (n = 68)	Hypothetical RCTs (n = 13)	Total (n=81)
**Clinical Setting**			
Neurology	39*	4	43
Cardiology	8	4	12
Obstetrics	3	1	4
Infection	3	0	3
Trauma	6	3	9
Critical care	9	1	10
**Type of interventions**			
Invasive / non-invasive	5 / 61	4 / 9	9 / 70
Unknown	2	0	2
**Trial design**			
Main RCT/ pilot RCT	65/3	n/a	65 / 3
Two groups / more than two groups	63 / 5	11 / 2	74 / 7
**Number of centres****			
Single centre / multi-centre / unknown	3 / 63 / 2	0 / 4 / 9	3 / 67 / 11
<20 centres	18		
20 -100 centres	33		
>100 centres	15		
Median number of centres (range)	45 (1 - 818)		
**Number of participants****			
<500 participants	22		
500 -1500 participants	32		
>1500 participants	14		
Median number of participants (range)	624 (4 - 58,050)		

*One recruitment paper included data from 32 Stroke host RCTs. RCT = randomised controlled trial

** Hypothetical RCT data did not include information beyond single or multicentre, nor the suggested no. of participants

For peer review only - http://bmjopen.bmj.com/site/about/guidelines.xhtml

Prospectively designed, non-randomised studies of interventions to optimise recruitment to one or more host RCTs (Category B)

The common rationale for this type of recruitment study design (Category B studies, Table 3) was the limitations of time when dealing with patients who required acute treatment. The need for urgent treatment was thought to hinder the ability of the study team to gain informed consent (IC) for trial participation.

The strategy of optimising information provision in the pre-hospital setting used in the one Category A study was mirrored by two Category B studies, which utilised the presence of pre-hospital staff to engage potential trial participants.(22, 24) Recruitment in these studies was reportedly optimised through provision of brief verbal information to participants as they travelled to hospital, and initial verbal consent that sought permission to deliver the emergency trial intervention. Further information was provided and written consent was subsequently obtained when the patients were stabilised, in hospital. One of these studies (24) also provided training to pre-hospital staff to improve their understanding of trial conduct, and devised a simple assessment of capacity to ensure that patients' initial verbal consent was valid.

The remaining prospectively designed (Category B) study focused on using technology to enhance recruitment during an influenza outbreak.(23) As patient numbers would be expected to rise rapidly across a wide geographical area, the study team devised a system which provided them with automated, real-time alerts whenever an eligible participant was identified in each hospital. This allowed one centralised study team to cover numerous study sites, enhancing recruitment opportunities. Although none of these studies provided numerical evidence of the effectiveness of their recruitment strategies, all authors concluded that their presented strategies were feasible and acceptable for use in UHA RCTs.

Studies describing recruitment experiences involving one or more host RCTs (Category C)

Rationales for reporting authors' experiences of recruitment were similar to those in prospectively designed studies, including the limited time available for consent (n=13), and recruitment difficulties caused by the clinical condition of the patients (n=18) (Category C studies, Table 4). Some studies were prompted by a host trial encountering recruitment difficulties (n=3). Of the 24 non-randomised studies describing recruitment experiences involving one or more host RCTs, 14 were observational and five were qualitative studies.

For peer review only - http://bmjopen.bmj.com/site/about/guidelines.xhtml

Table 3: Rationale and types of recruitment interventions reported in Category A or B studies (i.e. prospectively nested within RCTs)

Author and year	Rationale(s)	Descriptive summary of recruitment intervention(s)	Classification of recruitment intervention	Timing of information provision regarding host RCT	Timing of informed consent for host RCT	Timing of randomisation for host RCT
**Prospectively designed RCTs of interventions to optimise recruitment nested within one or more host RCTs (Category A studies)**						
Leira** 2009	Limited time for IC in host RCT due to the clinical condition requiring urgent treatment.	Intervention group:<br>'Advance notification' about host RCT using phone and fax prior to meeting pre-hospital team<br><br>Control group:<br>No information prior to meeting pre-hospital team	Advance notification using phone and fax	Intervention group:<br>'Advance notification' prior to face-to face meeting with pre-hospital team<br><br>Control group:<br>During face-to-face meeting with pre-hospital team	Intervention group:<br>During face-to-face meeting with pre-hospital team<br><br>Control group:<br>During face-to-face meeting with pre-hospital team	Intervention group:<br>Whilst patient in pre-hospital setting.<br><br>Control group:<br>Whilst patient in pre-hospital setting.
**Prospectively designed, non-randomised studies of interventions to optimise recruitment to one or more host RCTs (Category B studies)**						
Chow 2011	Recruitment team would be overwhelmed by covering multiple sites over a wide geographical area.	An automated service notified recruitment team in real time when a potentially eligible participant was identified across multiple centres	Automated service to facilitate real time notifications to recruitment team covering multiple sites	During face-to-face meeting with recruitment staff (once eligibility confirmed)	During face-to-face meeting with recruitment team	Whilst patient in hospital.
Shaw 2014	Limited time for IC in host RCT due to the clinical condition requiring urgent treatment.<br><br>Pre-hospital staff have limited experience of RCTs	Standardised verbal information was provided by pre-hospital staff before a simple capacity assessment, verbal consent and delivery of emergency treatment<br><br>Host RCT procedures were tailored towards pre-hospital staff, who also attended a one-day training event	Optimising information provision in pre-hospital setting.<br><br>Allowing verbal consent from patient or SDM.<br><br>Training of pre-hospital staff	Simple verbal information provided in pre-hospital setting.<br><br>Further full information provided in hospital	Initial verbal consent given in pre-hospital setting.<br><br>Written informed consent gained in hospital.	Whilst patient in pre-hospital setting.
Beshansky 2014	Limited time for IC in host RCT due to the clinical condition requiring urgent treatment.	Standardised verbal information was provided by pre-hospital staff before a simple capacity assessment, verbal consent and delivery of emergency treatment	Optimising information provision in pre-hospital setting.<br><br>Allowing verbal consent from patient.	Simple verbal information provided in pre-hospital setting.<br><br>Further full information provided in hospital	Initial verbal consent in pre-hospital setting.<br><br>Written informed consent in hospital.	Whilst patient in pre-hospital setting.

RCT = randomised controlled trial, IC= informed consent, SDM = surrogate decision maker
** Leira et al – patients were randomised to the *recruitment* intervention or control group prior to the recruitment team seeing the patient, and without consent from the participant.

For peer review only - http://bmjopen.bmj.com/site/about/guidelines.xhtml

Table 4: Frequency of rationales, study designs and recommendations from non-randomised studies describing recruitment experiences involving one or more host RCTs (i.e. Category C studies).

Recruitment study characteristic	Description	Frequency in Category C recruitment studies (n=24)**
**Rationale**	Patients too unwell to provide IC	18
	Limited time for IC in host RCT due to the clinical condition requiring urgent treatment	13
	Host RCT not meeting recruitment targets (at one or more sites) or terminated due to poor recruitment	3
	To better understand the impact of altering eligibility criteria on recruitment	2
	To better understand the impact of availability of SDMs on recruitment	2
	To better understand the recruitment process in a host RCT	1
	To better understand clinicians reasons for refusing patient participation in host RCT	1
**Recruitment study design**	Observational study of recruitment	14
	Qualitative studies of host participants/SDMs or PIS	5
	Survey of host RCT participants	2
	Survey of clinical staff involved in host RCT	1
	Simulation study evaluating the effect of altering eligibility criteria in multiple host RCTs	1
	Meta-analysis of recruitment data in host RCTs	1
**Recommendations for optimising recruitment in future RCTs or areas for further research into recruitment*****	To provide RCT information verbally and allow a verbal consent process	10
	To use a screening log can to provide insight into recruitment difficulties	8
	Patients or SDM were unable to recall key RCT information after providing IC***	5
	To use a 'waiver of consent'/ 'deferred consent'/ 'EFIC'	4
	To perform regular site visits	3
	To use a broad eligibility criteria /broad therapeutic window	2
	To use SDMs	2
	Novel methods for obtaining IC are required***	2
	To replace poorly recruiting centres	1
	To approach more eligible patients	1
	To survey staff involved with host RCT to provide insight into recruitment difficulties	1

RCT = randomised controlled trial, IC= informed consent, EFIC = exception from informed consent, SDM = surrogate decision maker, PIS= patient information sheet.
**each study may appear more than once in the relevant characteristics section (e.g. if it described more >1 rationale, or produced >1 finding/recommendation)
*** items for further research and not recommendations for optimising recruitment

For peer review only - http://bmjopen.bmj.com/site/about/guidelines.xhtml

These studies reported experiences of trial participants and surrogate decision makers (SDMs), or extracted verbatim information from written patient information sheets (PISs). Recommendations for optimising recruitment also mirrored Category A and B studies, highlighting the acceptability of verbal information provision and consent, or deferring consent altogether until an unwell patient is suitably stabilised. Additional benefits were seen in RCTs which used data from screening logs to uncover recruitment barriers, and trials which performed regular site visits.(29-31, 33, 35, 38, 39, 44) A sub section of qualitative studies, although not presenting recommendations for future trial conduct, highlighted their findings that many patients or SDMs who had provided consent to participate in an RCT did not recall much of the information provided to them during the consent process, suggesting that work was needed to improve consent in this setting.(31, 32, 40)

Studies to consider recruitment within proposed hypothetical RCTs (commonly known as community consultations) (Category D)

Eleven studies reported community views about proposed 'hypothetical' RCTs (Category D studies, Table 5). Although the rationales for conducting the studies were similar to studies involving real host RCTs (unwell patients and lack of time for consent), the study designs were varied including questionnaire surveys, interviews, and focus group meetings. Verbal information provision, verbal consent, and recruitment in the pre-hospital setting were identified as helpful recruitment strategies in these studies. However, these articles raised new issues around the appropriateness of using of SDMs when patients are too unwell to provide consent for themselves, and raised additional issues around who the SDM should be (next of kin (NOK), or an available physician). Some studies found a preference for the use of SDMs,(50, 52, 56) while others expressed that NOK or physicians should not be used for their specific hypothetical RCTs.(53, 55, 58)

Discussion

This review aimed to examine and summarise studies and methods used to optimise recruitment in RCTs in patients with an unscheduled hospital admission (UHA). It had the purpose of using the information to consider how to optimise recruitment in this challenging clinical setting in future studies. In the ORCCA database of recruitment research, only 39 out of a possible 3114 articles (1.25%) focused on recruitment to RCTs in the UHA setting. Only one of these studies was a randomised comparison of recruitment strategies; the majority of

For peer review only - http://bmjopen.bmj.com/site/about/guidelines.xhtml

Table 5: Frequency of rationales, study designs, main findings and recommendations from Non-randomised studies designed study to consider recruitment within proposed hypothetical RCTs (i.e. Category D studies, commonly known as community consultations).

Recruitment study characteristic	Description	Frequency in Category D recruitment studies (n=11)**
**Rationale**	Patients too unwell to provide IC	9
	Limited time for IC in host RCT due to the clinical condition requiring urgent treatment	6
	To explore the accuracy of decisions made by NOK when acting as SDM	1
**Recruitment study design**	Questionnaire survey	5
	Face-to-face interview	4
	Telephone survey	1
	Focus group meetings	1
**Recommendations for optimising recruitment in future RCTs or main findings*** **	To use a physician as a SDM	4
	To use NOK as a SDM	4
	To use EFIC	3
	**Not** to use NOK as a SDM	2
	To provide RCT information verbally and allow a verbal consent process	1
	To allow recruitment in pre-hospital setting	1
	To perform community consultations to estimate host RCT recruitment rates***	1
	**Not** to use a physician as a SDM	1
	**Not** to use EFIC	1
	To perform community consultations to aid selection of relevant study outcomes***	1

*RCT = randomised controlled trial, EFIC = exception from informed consent, NOK = next of kin, SDM = surrogate decision maker
**each study may appear more than once in the relevant characteristics section (e.g. if it described more >1 rationale, or produced >1 finding/recommendation)
*** items reported as main findings, but not recommendations for optimising recruitment

For peer review only - http://bmjopen.bmj.com/site/about/guidelines.xhtml

studies consisted of simple study designs describing recruitment experiences. Eleven further studies involved hypothetical RCTs, and whilst of some value, it is uncertain how these types of investigations translate into optimal RCT design. This work therefore highlights the need for development of interventions to optimise recruitment in the UHA setting and prospective evaluation of their effectiveness and acceptability.

Comparison to existing literature

There have been several reviews that have summarised the evidence for optimising recruitment in trials in other clinical conditions or contexts, including cancer (4-6), geriatrics (8, 9), primary care (7), and a mix of clinical settings.(11-17). Similar to our findings, these reviews have commonly highlighted the lack of high quality evaluations of recruitment interventions (6,16). Despite this, some of the reviews have identified effective recruitment strategies, although these vary in the extent to which they are likely to be transferrable to the UHA setting. For example, it is unclear if interventions such as telephone reminders (16,17), 'education sessions' about the health condition (11), and use of monetary incentives (11,17) are as effective or appropriate in the UHA context, given the specific factors that may compromise recruitment in this setting (e.g. patients in pain/distress, short timeframes for recruitment, busy settings, etc.). Other reviews have drawn attention to interventions aimed at recruiters, such as appropriate training/guidance (12), reduction of clinical workload (7,12), and 'research protected' time (12). These have potential to be helpful in trials conducted in the UHA setting, although further research is needed to examine the content/nature of the training materials needed, and the logistics of implementing these types of interventions. Finally, some reviews have shown that features of RCT study design – such as open, rather than placebo-controlled trials - are associated with better recruitment outcomes (16,17). Recommendations that advise against particular study designs may limit the quality of evidence generated to guide future patient care, and dissuade from the most important (and appropriate) clinical questions from being addressed. Furthermore, there is a growing body of evidence to indicate that it is possible to recruit to more complex RCTs with appropriate training and support.(59) More generally, use of integrated qualitative research to understand and address recruitment difficulties is being increasingly recommended in more recent reviews (12)(60), and is likely to inform novel insights if applied to trials in UHA settings.

For peer review only - http://bmjopen.bmj.com/site/about/guidelines.xhtml

Some reviews and individual studies have produced recommendations that are likely to be particularly relevant for developing UHA-specific recruitment strategies for future evaluation. These strategies may tackle some of the context-specific difficulties that are likely to be experienced in UHA settings. For example, one systematic review focusing on recruitment to RCTs involving patients with cancer or organ failure highlighted the potential for providing audio-visual information (such as a video to explain the RCT) to facilitate RCT recruitment (59). Such an approach may be helpful in the UHA setting, given that that large amounts of written information may not be appropriate in patients who are in pain or feeling distressed. This recommendation is also in keeping with guidance issued by the NHS Health Research Authority (HRA), which suggests using alternatives to written information (61).

A verbal exchange of information and providing initial verbal consent has been previously suggested as a preferred strategy to written alternatives in RCT taking place in the emergency setting (62). It is agreed that for UHAs this could be preferable, although it is considered that further work is needed to develop this type of verbal consent to ensure that quality assurance is still achieved for consent. Another suggested solution to this problem is the use of an independent patient advocate, who may oversee such conversations between trial team members and acutely unwell patients, to verify that appropriate information exchange took place, and to act as an assessor of a patient's willingness to participate in the RCT.(63) This strategy provides one potential solution to a significant obstacle in recruitment of patients undergoing an UHA. However, it may be practically difficult to achieve given that UHAs can occur at any time of the day or night and such trained patient advocates would also need to be available during these times making a trial more expensive.

Finally, a non-systematic review article focusing on recruitment to emergency medicine research also highlighted a similar problem in a lack of high quality evidence on recruitment. It suggested support for the use of deferred consent (also known as exception from informed consent) which was highlighted in this review and additionally raised the issue that the use of SDMs for consent may be problematic due to the pressures of time and the emotional stress family members will be under while a relative is acutely unwell.(3)

Strengths and weaknesses of this study

This review is the first to systematically focus on recruitment strategies in the UHA setting. With a reported growing number of unscheduled hospital admissions presenting great

For peer review only - http://bmjopen.bmj.com/site/about/guidelines.xhtml

challenges to modern-day health care provision, the conduct of RCTs in the UHA will inevitably develop as an area of research.

The review may be limited through its reliance on a single search of the ORRCA database, conducted in February 2016, at which point the database contained publications relevant to recruitment published up to the end of December 2014. The ORRCA database continues to evolve as updates encompass newly published recruitment research. Updates to the database may have generated further UHA research articles since the search for this review. It is possible that any new update could make an important and significant contribution to this field because so little has been done in this area thus far.

The review may be limited because a single researcher reviewed the majority of the ORRCA entries, and it is possible that ORRCA may not have included all relevant articles in the first place. This work may also be at risk of publication bias, as it excluded abstracts. However, inclusion of abstracts may have resulted in a lot of missing data as abstracts cannot contain all the data items we were interested in. Another weakness is that the majority of recruitment studies retrospective analyses of processes and events that occurred during the host RCT. These data were not necessarily collected with the intention of evaluating RCT recruitment strategies. This may limit the quality of the data and the utility of the recommendations arising from the included studies.

Finally, a risk of bias assessment of the included recruitment studies was not performed, because only one of the recruitment studies was an RCT (i.e. a randomised, controlled evaluation of a recruitment intervention).

Unanswered questions and future research

Some articles within this review demonstrated inconsistent conclusions about the value of surrogate decision makers, who these should be, and how these should operate. Future research should examine these issues in more depth, in a variety of clinical contexts, focusing on the roles of SDM in different RCTs and to what extent the public it acceptable for an SDM to decide on their behalf for trials involving acutely unwell patients.

It is uncertain how well the results of studies using hypothetical RCTs can be translated to the conduct of real RCTs. There may be some validity in the findings from hypothetical RCTs,

For peer review only - http://bmjopen.bmj.com/site/about/guidelines.xhtml

as highlighted in this review by the similarity of the results from studies which used real RCTs and hypothetical RCTs. Further work is required to ascertain the extent to which findings from studies which use hypothetical RCTs reliably influence the design and conduct of real RCTs.

As part of this review a classification system was devised in order to group together similarly designed recruitment research studies. Before any such classification system could be used more widely, it would require validation by testing its applicability to at least one further set of recruitment papers, preferably in a different health care setting.

This review highlighted strategies to deal with patients in the pre-hospital setting who required urgent treatment. However, not all patients who are cared for in the pre-hospital setting require treatment immediately. Some may require transport to hospital for further assessment and potential treatment. Further research could explore whether providing early trial information to such patients based on their symptoms or presumed diagnosis, could affect trial recruitment, should treatment be required later. This strategy could prove to be useful in a broader range of unscheduled hospital admissions.

Although some recommendations for optimising recruitment could be drawn from this review, the overall lack of research in this area, particularly amongst high quality, methodologically robust studies, is a limiting factor. Future recruitment studies in this clinical setting should focus on studies with higher methodological rigour, by developing novel interventions to optimise recruitment and prospectively evaluating their effectiveness through an appropriate study design.

For peer review only

Conclusion

There is a relative paucity of high quality research on strategies to optimise recruitment to RCTs involving unscheduled hospital admissions. Some emerging recommendations include optimising information provision about the trial in the pre-hospital setting to improve recruitment where treatment is required urgently, or using technology to facilitate recruitment across many hospital sites. Screening log data can also provide useful insight to specific barriers to recruitment. Future research in this setting should focus on conducting

For peer review only - http://bmjopen.bmj.com/site/about/guidelines.xhtml

studies with higher methodological rigour, by developing interventions to optimise recruitment and prospectively evaluating their effectiveness.

Acknowledgements

The following are gratefully acknowledged for their assistance and support with this work: Shaun Treweek, Anna Kearney, Nicola Harman.

Funding

CR is supported by the Medical Research Council (MRC) Hub for Trials Methodology Research Network and the Collaboration and Innovation for Difficult Trials in Invasive procedures (ConDuCT-II) Hub for Trials Methodology Research (MR/K025643/1). The funders had no role in study design, data collection and analysis, decision to publish, or preparation of manuscript.

Contributor statement

CR, LR JR, and JMB designed the study and formulated the protocol. CR and KF performed all data extraction, which was reviewed and critically analysed by CR, LR and JMB. CG facilitated access to the ORRCA database and critically appraised the review. CR produced the first draft of the manuscript and LR and JMB made substantial contributions to the formulation of the final version. All authors read and approved the final manuscript.

Competing interests

No competing interests

Data sharing statement

As this is a review of published literature, there are no additional data available.

Figure 1: Study selection PRISMA flow diagram

References

1. Tudur Smith C, Hickey H, Clarke M, Blazeby J, Williamson P. The trials methodological research agenda: results from a priority setting exercise. Trials. 2014;15:32-.

2. Schandelmaier S, von Elm E, You JJ, Blumle A, Tomonaga Y, Lamontagne F, et al. Premature Discontinuation of Randomized Trials in Critical and Emergency Care: A Retrospective Cohort Study. Critical care medicine. 2016;44(1):130-7.

For peer review only - http://bmjopen.bmj.com/site/about/guidelines.xhtml

3. Cofield SS, Conwit R, Barsan W, Quinn J. Recruitment and retention of patients into emergency medicine clinical trials. Academic emergency medicine : official journal of the Society for Academic Emergency Medicine. 2010;17(10):1104-12.
4. Boland J, Currow DC, Wilcock A, Tieman J, Hussain JA, Pitsillides C, et al. A systematic review of strategies used to increase recruitment of people with cancer or organ failure into clinical trials: implications for palliative care research. Journal of pain and symptom management. 2015;49(4):762-72.e5.
5. Fayter D, McDaid C, Eastwood A. A systematic review highlights threats to validity in studies of barriers to cancer trial participation. Journal of clinical epidemiology. 2007;60(10):990-1001.
6. Mc Daid C, Hodges Z, Fayter D, Stirk L, Eastwood A. Increasing participation of cancer patients in randomised controlled trials: a systematic review. Trials. 2006;7:16.
7. Ngune I, Jiwa M, Dadich A, Lotriet J, Sriram D. Effective recruitment strategies in primary care research: a systematic review. Quality in primary care. 2012;20(2):115-23.
8. Auster J, Janda M. Recruiting older adults to health research studies: A systematic review. Australasian journal on ageing. 2009;28(3):149-51.
9. Provencher V, Mortenson WB, Tanguay-Garneau L, Belanger K, Dagenais M. Challenges and strategies pertaining to recruitment and retention of frail elderly in research studies: a systematic review. Archives of gerontology and geriatrics. 2014;59(1):18-24.
10. Ibrahim S, Sidani S. Strategies to recruit minority persons: a systematic review. Journal of immigrant and minority health / Center for Minority Public Health. 2014;16(5):882-8.
11. Caldwell PH, Hamilton S, Tan A, Craig JC. Strategies for increasing recruitment to randomised controlled trials: systematic review. PLoS medicine. 2010;7(11):e1000368.
12. Fletcher B, Gheorghe A, Moore D, Wilson S, Damery S. Improving the recruitment activity of clinicians in randomised controlled trials: a systematic review. BMJ open. 2012;2(1):e000496.
13. Huynh L, Johns B, Liu SH, Vedula SS, Li T, Puhan MA. Cost-effectiveness of health research study participant recruitment strategies: a systematic review. Clinical trials (London, England). 2014;11(5):576-83.
14. Mapstone J, Elbourne D, Roberts I. Strategies to improve recruitment to research studies. The Cochrane database of systematic reviews. 2007(2):Mr000013.
15. Raftery J, Bryant J, Powell J, Kerr C, Hawker S. Payment to healthcare professionals for patient recruitment to trials: systematic review and qualitative study. Health technology assessment (Winchester, England). 2008;12(10):1-128, iii.
16. Treweek S, Mitchell E, Pitkethly M, Cook J, Kjeldstrom M, Taskila T, et al. Strategies to improve recruitment to randomised controlled trials. The Cochrane database of systematic reviews. 2010(1):Mr000013.
17. Watson JM, Torgerson DJ. Increasing recruitment to randomised trials: a review of randomised controlled trials. BMC medical research methodology. 2006;6:34.

For peer review only

For peer review only - http://bmjopen.bmj.com/site/about/guidelines.xhtml

18. Burns KE, Prats CJ, Maione M, Lanceta M, Zubrinich C, Jeffs L, et al. The Experience of Surrogate Decision Makers on Being Approached for Consent for Patient Participation in Research. A Multicenter Study. Annals of the American Thoracic Society. 2017;14(2):238-45.
19. Whitesides LW, Baren JM, Biros MH, Fleischman RJ, Govindarajan PR, Jones EB, et al. Impact of individual clinical outcomes on trial participants' perspectives on enrollment in emergency research without consent. Clinical trials (London, England). 2017;14(2):180-6.
20. Fordyce CB, Roe MT, Dickert NW. Maximizing value and minimizing barriers: Patient-centered community consultation for research in emergency settings. Clinical trials (London, England). 2017;14(1):88-93.
21. Leira EC, Ahmed A, Lamb DL, Olalde HM, Callison CR, Torner JC, et al. Extending Acute Trials to Remote Populations A Pilot Study During Interhospital Helicopter Transfer. Stroke. 2009;40(3):895-901.
22. Beshansky JR, Sheehan PR, Klima KJ, Hadar N, Vickery EM, Selker HP. A community consultation survey to evaluate support for and success of the IMMEDIATE trial. Clinical Trials. 2014;11(2):178-86.
23. Chow E, Zuberi M, Seto R, Hota S, Fish EN, Morra D. Using real-time alerts for clinical trials Identifying potential study subjects. Applied Clinical Informatics. 2011;2(4):472-80.
24. Shaw L, Price C, McLure S, Howel D, McColl E, Younger P, et al. Paramedic Initiated Lisinopril For Acute Stroke Treatment (PIL-FAST): results from the pilot randomised controlled trial. Emerg Med J. 2014;31(12):994-9.
25. Abramson NS, Safar P. Deferred consent: use in clinical resuscitation research. Brain Resuscitation Clinical Trial II Study Group. Annals of Emergency Medicine. 1990;19(7):781-4.
26. Adeoye O, Pancioli A, Khoury J, Moomaw CJ, Schmit P, Ewing I, et al. Efficiency of Enrollment in a Successful Phase II Acute Stroke Clinical Trial. Journal of Stroke & Cerebrovascular Diseases. 2012;21(8):667-72.
27. Ågård A, Hermerén G, Herlitz J. Patients' experiences of intervention trials on the treatment of myocardial infarction: Is it time to adjust the informed consent procedure to the patient's capacity? Heart. 2001;86(6):632-7.
28. Annane D, Outin H, Fisch C, Bellissant E. The effect of waiving consent on enrollment in a sepsis trial. Intensive Care Medicine. 2004;30(2):321-4.
29. Bellomo R, Trial RRT. Screening and Study Enrolment in the Randomized Evaluation of Normal vs. Augmented Level (RENAL) Replacement Therapy Trial. Blood Purification. 2009;27(2):199-205.
30. Burns KEA, Zubrinich C, Tan WL, Raptis S, Xiong W, Smith O, et al. Research Recruitment Practices and Critically Ill Patients A Multicenter, Cross-Sectional Study (The Consent Study). American Journal of Respiratory and Critical Care Medicine. 2013;187(11):1212-8.
31. Chlan L, Guttormson J, Tracy MF, Bremer KL. STRATEGIES FOR OVERCOMING SITE AND RECRUITMENT CHALLENGES IN RESEARCH STUDIES BASED IN INTENSIVE CARE UNITS. American Journal of Critical Care. 2009;18(5):410-7.
32. Collins JF. Data and safety monitoring board issues raised in the VA Status Epilepticus Study. Controlled Clinical Trials. 2003;24(1):71-7.

For peer review only - http://bmjopen.bmj.com/site/about/guidelines.xhtml

33. Cook D, Arabi Y, Ferguson ND, Heels-Ansdell D, Freitag A, McDonald E, et al. Physicians declining patient enrollment in a critical care trial: a case study in thromboprophylaxis. Intensive Care Medicine. 2013;39(12):2115-25.
34. Costescu DJW, Cullimore AJ. Lessons learned from a resident-led clinical trial in obstetrics. Clinical Trials. 2013;10(4):612-6.
35. Crowley ST, Chertow GM, Vitale J, O'Connor T, Zhang JN, Schein RMH, et al. Lessons for successful study enrollment from the Veterans Affairs/National Institutes of Health Acute Renal Failure Trial Network Study. Clinical Journal of the American Society of Nephrology. 2008;3(4):955-61.
36. Elkins JS, Khatabi T, Fung L, Rootenberg J, Johnston SC. Recruiting subjects for acute stroke trials: a meta-analysis. Stroke [Internet]. 2006; 37(1):[123-8 pp.]. Available from: http://onlinelibrary.wiley.com/o/cochrane/clcmr/articles/CMR-10639/frame.html
http://stroke.ahajournals.org/content/37/1/123.full.pdf.
37. Flaherty ML, Karlawish J, Khoury JC, Kleindorfer D, Woo D, Broderick JP. How important is surrogate consent for stroke research? Neurology. 2008;71(20):1566-71.
38. Foster D, Cook D, Granton J, Steinberg M, Marshall J. Use of a screen log to audit patient recruitment into multiple randomized trials in the intensive care unit. Canadian Critical Care Trials Group. Critical care medicine [Internet]. 2000; 28(3):[867-71 pp.]. Available from: http://onlinelibrary.wiley.com/o/cochrane/clcmr/articles/CMR-11625/frame.html.
39. Glassberg AE, Luce JM, Matthay MA. Reasons for nonenrollment in a clinical trial of acute lung injury. Chest [Internet]. 2008; 134(4):[719-23 pp.]. Available from: http://onlinelibrary.wiley.com/o/cochrane/clcmr/articles/CMR-12450/frame.html
http://journal.publications.chestnet.org/data/Journals/CHEST/22076/zcb01008000719.pdf.
40. Kendall B, Stadeli R, Schegg B, Olbrich M, Chen E, Harmelin-Kadouri R, et al. Clinical Trial Educator program - a novel approach to accelerate enrollment in a phase III International Acute Coronary Syndrome Trial. Clinical Trials. 2012;9(3):358-66.
41. Kenyon S, Dixon-Woods M, Jackson CJ, Windridge K, Pitchforth E. Participating in a trial in a critical situation: a qualitative study in pregnancy. Quality & Safety in Health Care. 2006;15(2):98-101.
42. Roozenbeek B, Maas AIR, Marmarou A, Butcher I, Lingsma HF, Lu J, et al. The influence of enrollment criteria on recruitment and outcome distribution in traumatic brain injury studies: results from the impact study. Journal of Neurotrauma [Internet]. 2009; 26(7):[1069-75 pp.]. Available from: http://onlinelibrary.wiley.com/o/cochrane/clcmr/articles/CMR-14708/frame.html
http://online.liebertpub.com/doi/pdfplus/10.1089/neu.2008.0569.
43. Schats R, Brilstra EH, Rinkel GJ, Algra A, Gijn J. Informed consent in trials for neurological emergencies: the example of subarachnoid haemorrhage. Journal of Neurology, Neurosurgery, and Psychiatry [Internet]. 2003; 74(7):[988-91 pp.]. Available from: http://onlinelibrary.wiley.com/o/cochrane/clcmr/articles/CMR-12831/frame.html
http://www.ncbi.nlm.nih.gov/pmc/articles/PMC1738516/pdf/v074p00988.pdf.

For peer review only - http://bmjopen.bmj.com/site/about/guidelines.xhtml

44. Smith OM, McDonald E, Zytaruk N, Foster D, Matte A, Clarke F, et al. Rates and determinants of informed consent: A case study of an international thromboprophylaxis trial. Journal of Critical Care. 2013;28(1):28-39.
45. Smyth RM, Jacoby A, Elbourne D. Deciding to join a perinatal randomised controlled trial: experiences and views of pregnant women enroled in the Magpie Trial. Midwifery. 2012;28(4):E478-85.
46. Williams BF, French JK, White HD. Informed consent during the clinical emergency of acute myocardial infarction (HERO-2 consent substudy): A prospective observational study. Lancet. 2003;361(9361):918-22.
47. Yamal JM, Robertson CS, Rubin ML, Benoit JS, Hannay HJ, Tilley BC. Enrollment of racially/ethnically diverse participants in traumatic brain injury trials: Effect of availability of exception from informed consent. Clinical Trials. 2014;11(2):187-94.
48. Yuval R, Halon DA, Merdler A, Khader N, Karkabi B, Uziel K, et al. Patient comprehension and reaction to participating in a double-blind randomized clinical trial (ISIS-4) in acute myocardial infarction. Archives of Internal Medicine. 2000;160(8):1142-6.
49. Abboud PA, Heard K, Al-Marshad AA, Lowenstein SR. What determines whether patients are willing to participate in resuscitation studies requiring exception from informed consent? Journal of Medical Ethics [Internet]. 2006; 32(8):[468-72 pp.]. Available from: http://onlinelibrary.wiley.com/o/cochrane/clcmr/articles/CMR-10612/frame.html.
50. Ali K, Roffe C, Crome P. What patients want: consumer involvement in the design of a randomized controlled trial of routine oxygen supplementation after acute stroke. Stroke [Internet]. 2006; 37(3):[865-71 pp.]. Available from: http://onlinelibrary.wiley.com/o/cochrane/clcmr/articles/CMR-10638/frame.html
http://stroke.ahajournals.org/content/37/3/865.full.pdf.
51. Bulger EM, Schmidt TA, Cook AJ, Brasel KJ, Griffiths DE, Kudenchuk PJ, et al. The Random Dialing Survey as a tool for community consultation for research involving the emergency medicine exception from informed consent. Annals of Emergency Medicine [Internet]. 2009; 53(3):[341-50 pp.]. Available from: http://onlinelibrary.wiley.com/o/cochrane/clcmr/articles/CMR-14468/frame.html
http://ac.els-cdn.com/S0196064408015126/1-s2.0-S0196064408015126-main.pdf?_tid=3127512a-62ab-11e5-948a-00000aab0f27&acdnat=1443092490_2b79f56df4905bd0b2acd045224491cf.
52. Clark DJ, Kolias AG, Corteen EA, Ingham SC, Piercy J, Crick SJ, et al. Community consultation in emergency neurotrauma research: results from a pre-protocol survey. Acta Neurochirurgica. 2013;155(7):1329-34; discussion 34.
53. Giudice AD, Plaum J, Maloney E, Kasner SE, Le Roux PD, Baren JM. Who will consent to emergency treatment trials for subarachnoid hemorrhage? Academic Emergency Medicine. 2009;16(4):309-15.
54. Goldstein JN, Espinola JA, Fisher J, Pallin DJ, Camargo Jr CA. Public opinion of a stroke clinical trial using exception from informed consent. International Journal of Emergency Medicine. 2010;3(4):385-9.
55. Newman JT, Smart A, Reese TR, Williams A, Moss M. Surrogate and patient discrepancy regarding consent for critical care research. Critical care medicine. 2012;40(9):2590-4.

For peer review only - http://bmjopen.bmj.com/site/about/guidelines.xhtml

56. Scotton WJ, Kolias AG, Ban VS, Crick SJ, Sinha R, Gardner A, et al. Community consultation in emergency neurosurgical research: lessons from a proposed trial for patients with chronic subdural haematomas. British Journal of Neurosurgery. 2013;27(5):590-4.
57. Sims CA, Isserman JA, Holena D, Sundaram LM, Tolstoy N, Greer S, et al. Exception from informed consent for emergency research: Consulting the trauma community. Journal of Trauma and Acute Care Surgery. 2013;74(1):157-66.
58. Snowdon C, Elbourne D, Forsey M, Alfirevic Z. Views of emergency research (VERA): A qualitative study of women and their partners' views of recruitment to trials in severe postpartum haemorrhage. Midwifery. 2012;28(6):800-8.
59. Donovan JL, Rooshenas L, Jepson M, Elliott D, Wade J, Avery K, et al. Optimising recruitment and informed consent in randomised controlled trials: the development and implementation of the Quintet Recruitment Intervention (QRI). Trials. 2016;17(1):283.
60. Elliott D, Husbands S, Hamdy FC, Holmberg L, Donovan JL. Understanding and Improving Recruitment to Randomised Controlled Trials: Qualitative Research Approaches. Eur Urol. 2017.
61. Authority NHR. Applying a proportionate approach to the process of seeking consent - HRA Guidance. 2017.
62. Roberts I, Prieto-Merino D, Shakur H, Chalmers I, Nicholl J. Effect of consent rituals on mortality in emergency care research. Lancet. 2011;377(9771):1071-2.
63. Sahan KM, Channon KM, Choudhury RP, Kharbanda RK, Lee R, Sheehan M. Refining the Enrolment Process in Emergency Medicine Research. Eur J Cardiovasc Med. 2016;4(1):506-10.

For peer review only

For peer review only - http://bmjopen.bmj.com/site/about/guidelines.xhtml

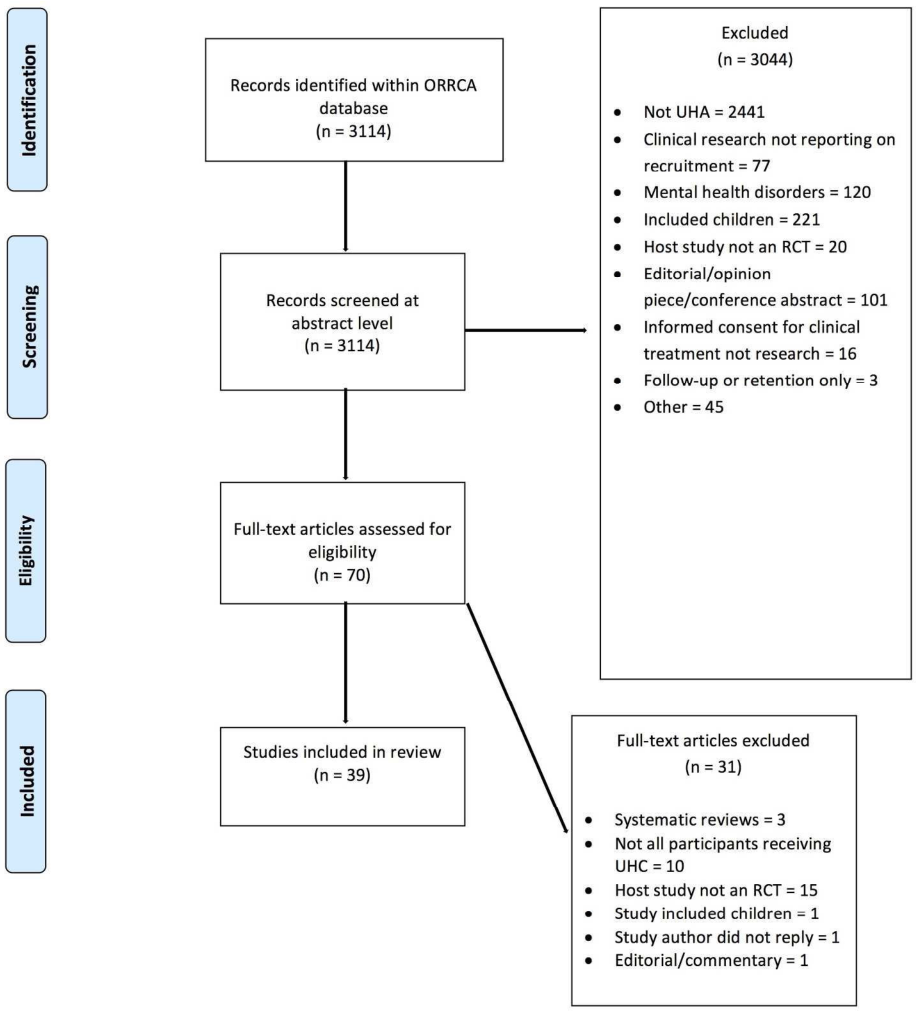

Figure 1: Study selection PRISMA flow diagram

208x242mm (300 x 300 DPI)

	#	Checklist item	Reported on page #
**TITLE**			
Title	1	Identify the report as a systematic review, meta-analysis, or both.	1
**ABSTRACT**			
Structured summary	2	Provide a structured summary including, as applicable: background; objectives; data sources; study eligibility criteria, participants, and interventions; study appraisal and synthesis methods; results; limitations; conclusions and implications of key findings; systematic review registration number.	2
**INTRODUCTION**			
Rationale	3	Describe the rationale for the review in the context of what is already known.	3
Objectives	4	Provide an explicit statement of questions being addressed with reference to participants, interventions, comparisons, outcomes, and study design (PICOS).	5
**METHODS**			
Protocol and registration	5	Indicate if a review protocol exists, if and where it can be accessed (e.g., Web address), and, if available, provide registration information including registration number.	N/A
Eligibility criteria	6	Specify study characteristics (e.g., PICOS, length of follow-up) and report characteristics (e.g., years considered, language, publication status) used as criteria for eligibility, giving rationale.	5-7
Information sources	7	Describe all information sources (e.g., databases with dates of coverage, contact with study authors to identify additional studies) in the search and date last searched.	4-5
Search	8	Present full electronic search strategy for at least one database, including any limits used, such that it could be repeated.	4-5
Study selection	9	State the process for selecting studies (i.e., screening, eligibility, included in systematic review, and, if applicable, included in the meta-analysis).	5-6

For peer review only

Data collection process	10	Describe method of data extraction from reports (e.g., piloted forms, independently, in duplicate) and any processes for obtaining and confirming data from investigators.	7
Data items	11	List and define all variables for which data were sought (e.g., PICOS, funding sources) and any assumptions and simplifications made.	7
Risk of bias in individual studies	12	Describe methods used for assessing risk of bias of individual studies (including specification of whether this was done at the study or outcome level), and how this information is to be used in any data synthesis.	N/A
Summary measures	13	State the principal summary measures (e.g., risk ratio, difference in means).	N/A
Synthesis of results	14	Describe the methods of handling data and combining results of studies, if done, including measures of consistency (e.g., I^2) for each meta-analysis.	N/A

Section/topic	#	Checklist item	Reported on page #
Risk of bias across studies	15	Specify any assessment of risk of bias that may affect the cumulative evidence (e.g., publication bias, selective reporting within studies).	N/A
Additional analyses	16	Describe methods of additional analyses (e.g., sensitivity or subgroup analyses, meta-regression), if done, indicating which were pre-specified.	N/A
**RESULTS**			
Study selection	17	Give numbers of studies screened, assessed for eligibility, and included in the review, with reasons for exclusions at each stage, ideally with a flow diagram.	7
Study characteristics	18	For each study, present characteristics for which data were extracted (e.g., study size, PICOS, follow-up period) and provide the citations.	8-10
Risk of bias within studies	19	Present data on risk of bias of each study and, if available, any outcome level assessment (see item 12).	N/A
Results of individual studies	20	For all outcomes considered (benefits or harms), present, for each study: (a) simple summary data for each intervention group (b) effect estimates and confidence intervals, ideally with a forest plot.	8-10

For peer review only

Synthesis of results	21	Present results of each meta-analysis done, including confidence intervals and measures of consistency.	N/A
Risk of bias across studies	22	Present results of any assessment of risk of bias across studies (see Item 15).	N/A
Additional analysis	23	Give results of additional analyses, if done (e.g., sensitivity or subgroup analyses, meta-regression [see Item 16]).	N/A
**DISCUSSION**			
Summary of evidence	24	Summarize the main findings including the strength of evidence for each main outcome; consider their relevance to key groups (e.g., healthcare providers, users, and policy makers).	10
Limitations	25	Discuss limitations at study and outcome level (e.g., risk of bias), and at review-level (e.g., incomplete retrieval of identified research, reporting bias).	11-12
Conclusions	26	Provide a general interpretation of the results in the context of other evidence, and implications for future research.	13
**FUNDING**			
Funding	27	Describe sources of funding for the systematic review and other support (e.g., supply of data); role of funders for the systematic review.	13-14

From: Moher D, Liberati A, Tetzlaff J, Altman DG, The PRISMA Group (2009). Preferred Reporting Items for Systematic Reviews and Meta-Analyses: The PRISMA Statement. PLoS Med 6(7): e1000097. doi:10.1371/journal.pmed1000097 N/A= not applicable

For more information, visit: **www.prisma-statement.org**.

For peer review only

For peer review only

BMJ Open

A detailed systematic analysis of recruitment strategies in randomised controlled trials in patients with an unscheduled admission to hospital

Journal:	*BMJ Open*
Manuscript ID	bmjopen-2017-018581.R2
Article Type:	Research
Date Submitted by the Author:	09-Oct-2017
Complete List of Authors:	Rowlands, Ceri; MRC ConDuCT-II Hub for Trials Methodology Research, School of Social & Community Medicine<br>Rooshenas, Leila; MRC ConDuCT-II Hub for Trials Methodology Research, School of Social & Community Medicine, University of Bristol<br>Fairhurst, Katherine; MRC ConDuCT-II Hub for Trials Methodology Research, School of Social & Community Medicine<br>Rees, Jonathan; University Hospitals Bristol NHS Foundation Trust, Division of Surgery, Head & Neck<br>Gamble, Carrol; University of Liverpool, MRC North West Hub for Trials Methodology Research<br>Blazeby, Jane; University of Bristol, Department of Social Medicine
**Primary Subject Heading**:	Research methods
Secondary Subject Heading:	Evidence based practice, Emergency medicine, Health services research
Keywords:	Clinical trials < THERAPEUTICS, STATISTICS & RESEARCH METHODS, ACCIDENT & EMERGENCY MEDICINE, QUALITATIVE RESEARCH

SCHOLARONE™ Manuscripts

A detailed systematic analysis of recruitment strategies in randomised controlled trials in patients with an unscheduled admission to hospital

C Rowlands[1], L Rooshenas[1], K Fairhurst[1], J Rees[2], C Gamble[3], JM Blazeby[1,2]

1. MRC ConDuCT-II Hub for Trials Methodology Research, School of Social & Community Medicine, University of Bristol, UK
2. Division of Surgery, Head & Neck, University Hospitals Bristol NHS Foundation Trust, Bristol, UK
3. MRC North West Hub for Trials Methodology Research, Institute of Translational Medicine, University of Liverpool, UK

Corresponding author: Leila.Rooshenas@bristol.ac.uk

Word count = 3330 excluding tables, figures and references.

For peer review only - http://bmjopen.bmj.com/site/about/guidelines.xhtml

For peer review only

Abstract

Objectives To examine the design and findings of recruitment studies in RCTs involving patients with an unscheduled admission to hospital (UHA), to consider how to optimise recruitment in future RCTs of this nature.

Design Studies within the ORRCA database (Online Resource for Recruitment Research in Clinical Trials; www.orrca.org.uk) which reported on recruitment to RCTs involving UHAs in patients >18 years were included. Extracted data included trial clinical details, and the rationale and main findings of the recruitment study.

Studies were categorised according to the design of the recruitment study and the nature of the host trials (real or hypothetical). Data on the rationale for the recruitment study and the methods used to optimise recruitment were collected and summarised.

Results Of 3114 articles populating ORRCA, 39 recruitment studies were eligible, focusing on 68 real and 13 hypothetical host RCTs. Four studies were prospectively planned investigations of recruitment interventions, one of which was a nested RCT. Most recruitment papers were reports of recruitment experiences from one or more 'real' RCTs (n=24) or studies using hypothetical RCTs (n=11). Rationales for conducting recruitment studies included limited time for informed consent (IC) and patients being too unwell to provide IC. Methods to optimise recruitment included providing patients with trial information in the pre-hospital setting, technology to allow recruiters to cover multiple sites, screening logs to uncover recruitment barriers, and verbal rather than written information and consent.

Conclusion There is a paucity of high quality research into recruitment in RCTs involving UHAs with only one nested randomised study evaluating a recruitment intervention. Amongst the remaining studies methods to optimise recruitment focused on how to improve information provision in the pre-hospital setting and use of screening logs. Future research in this setting should focus on the prospective evaluation of the well-developed interventions to optimise recruitment.

Abstract word count = 297 words

For peer review only - http://bmjopen.bmj.com/site/about/guidelines.xhtml

Strengths and limitations of this study

- This review is the first to focus on the complex issue of recruitment to RCTs involving patients undergoing an unscheduled hospital admission
- This review is the first publication to utilise the ORRCA database in generating recruitment research
- The ORRCA database continues to evolve as updates encompass newly published recruitment research. Updates to the database may have generated further UHA research articles since the completion of this review.

For peer review only

For peer review only - http://bmjopen.bmj.com/site/about/guidelines.xhtml

Introduction

Recruitment to randomised controlled trials (RCTs) is the biggest obstacle to successful trial conduct.(1) Recruitment may be particularly challenging amongst patients who have an unscheduled hospital admission (UHA). In this situation, the condition of the potential participant and the demanding working environment for clinical teams can complicate the process of identification, screening, and informed consent. Patients are often in pain, unwell, and anxious about the underlying problem. There may also be time limitations due to the urgent need to deliver the clinical treatments under evaluation.(2, 3)

There are a number of existing systematic reviews of methods to optimise recruitment to trials in a variety of clinical contexts and patient populations, including cancer (4-6), primary care, (7) geriatrics (8, 9) and minority community patients (10), or a mix of clinical settings.(11-17) None specifically focus on recruitment of patients undergoing UHA.

Established methods exist for recruiting potential RCT participants who are unwell or unconscious and lack capacity. In these circumstances, permission for enrolment into an RCT may be sought from a surrogate decision maker (SDM) (18), or through deferred consent (also known as 'exception from informed consent'): a process whereby a participant is recruited into the trial in order for urgent treatment to be provided and subsequently asked to provide written consent for ongoing participation once they regain capacity.(19) However, recruitment may be particularly challenging where patients are acutely unwell, but retain capacity to decide on enrolment into an RCT. Reviewing the literature on how to optimise recruitment in this setting may lead to valuable insights, and identify areas where further research is needed.

The aim of this paper was to examine the design and findings of recruitment studies in RCTs involving patients with an unscheduled admission to hospital (UHA), to consider how to optimise recruitment in future RCTs of this nature..

Methods

Search strategy

Articles were identified through manually screening each entry within the ORRCA recruitment research database (Online Resource for Recruitment research in Clinical triAls;

For peer review only - http://bmjopen.bmj.com/site/about/guidelines.xhtml

http://www.orrca.org.uk/). The ORRCA project was funded by the UK Medical Research Council (MRC) Hubs for Trials Methodology Research Network (HTMRN). It provides a comprehensive online database of published empirical and non- empirical papers about recruitment to clinical research. ORRCA is populated from an extensive systematic search of the Cochrane Library, MEDLINE (Ovid), SCOPUS, ERIC and SCI-EXPANDED and SSCI (via ISI Web of Science). The search strategy employed by ORRCA was based on a Cochrane systematic review of trial recruitment.(16) Further details about the formation of the ORRCA database is reported on their website (http://www.orrca.org.uk/). In this review, a full up-to-date copy of the ORRCA database was obtained in January 2016, and the database was searched in February 2016. At this stage ORRCA contained publications relevant to recruitment published up to and including the end of December 2014. Publications from 2015 onwards were not available due to ongoing work in processing articles in the ORRCA database.

Study eligibility criteria

Any study held within ORRCA that reported on recruitment to RCTs involving UHAs was eligible for inclusion. UHA was defined as an unscheduled admission to hospital at short notice because of clinical need. This included pre-hospital care, intensive care (ICU) admissions, and accident and emergency (A&E) department attendances. Studies which reported on a mix of patients undergoing scheduled and unscheduled admissions were eligible if the findings for the UHA study population were described separately. Papers that reported recruitment strategies for a mixture of RCTs and other types of research (i.e. non-RCTs) were only eligible for inclusion if the recruitment strategies for RCTs were described separately. RCTs that involved children (age <18 years) or patients with acute psychiatric illness were excluded, as these patients would not normally be subject to the usual recruitment processes due to differences in the consent processes. Systematic reviews of methods to optimise recruitment were scrutinised for relevant primary articles, but not included in the analysis. Editorials, and studies of recruitment to non-RCTs were excluded. Abstracts were also excluded because these rarely included the necessary contextual information and data needed to make a meaningful contribution to the dataset for this study.

For peer review only - http://bmjopen.bmj.com/site/about/guidelines.xhtml

Screening and selection process

One author (CR) screened all articles included within the ORRCA database. Duplicate screening was carried out by one other author (KF) on 10% of the database. KF was blinded to the original screening decisions. Papers were assessed at title and abstract level according to the eligibility criteria. Differences in opinion were resolved by discussion between CR and KF, and any remaining differences in opinion were referred to another member of the study team (JMB) if required. The aim was to reach agreement for screening decisions on all studies within this sample. Agreement was reached on 271/300 articles screened. Of the 29 discrepancies raised, 22 were resolved following discussion between CR and KF. The seven remaining papers were discussed with a third author (JMB), which resulted in two of these papers being included and five being excluded. No paper which was suggested to be included by the second reviewer was eventually included in the review.

We calculated a kappa statistic for the double screened articles above. Ten per cent of articles were double screened with a Kappa = 0.677 (SE= 0.048, p <0.001) suggesting "good" agreement. As such, the remaining 90% of articles in the ORRCA database were screened by a single screener (CR). The third arbiter (JMB) involved in screening the 10% sample was also consulted for screening the remaining 90% of articles in the ORRCA database, in instances where the single screener (CR) was uncertain about an article's eligibility.

Definitions – host RCT

All studies focused on recruitment to one or more specific 'host' RCTs. In this paper, a host RCT refers to the underlying randomised controlled trial, (i.e. addressing a clinical question) in which the recruitment of participants took place. A host RCT could be a pilot or a main trial. Some recruitment papers reported on community consultations in which the views of the public were sought to establish the likelihood of recruitment success or acceptability of a proposed trial. This approach is typically used when the study population may be critically ill at the time of recruitment (and therefore may be unable to provide full, written informed consent).(20) In recognition of this, a clear differentiation was made between studies that focused on recruitment to an existing clinical RCT (a 'real' host RCT) versus potential recruitment to an RCT that did not exist (a 'hypothetical' host RCT), but is proposed to exist in order to estimate its acceptability to potential participants. A 'recruitment study' refers to research into the process of recruiting eligible participants, in the context of one or more real or hypothetical host RCTs.

For peer review only - http://bmjopen.bmj.com/site/about/guidelines.xhtml

Definitions - recruitment study design

In order to group similarly designed recruitment studies together and enhance data analysis a new categorisation system for different recruitment study designs was developed (Categories A to D). Consideration was given to the design of the recruitment study and whether a real or hypothetical host RCT was used. The categories are provided in Table 1.

Contacting study authors

If an appropriate recruitment study did not adequately describe the host trial, the study authors were contacted by email to determine whether the host trial met the eligibility criteria. Three attempts, each two weeks apart, were made to contact study authors. If no response was received, then the paper was excluded.

Data Extraction and Synthesis

CR extracted the data using a pre-designed and piloted data extraction form. Data extracted from eligible studies included clinical details of the underlying host RCT, the rationale for conducting a recruitment study, a summary of the recruitment study findings, recommendations for improving recruitment, and suggestions for further research. Where specific recruitment interventions had been evaluated, further details regarding the interventions were collected, including the timing of information exchange, informed consent, and randomisation. No statistical analyses were planned, as the review was expected to provide a descriptive analysis of results due to the anticipated heterogeneous nature of recruitment strategies presented. During the process, multiple meetings were undertaken with JB to examine papers and check data extraction processes as required.

Results

Study selection

A total of 3114 articles were identified within the ORRCA database. After initial screening at title and abstract level, 3044 articles were excluded, leaving 70 potentially eligible for which full texts were obtained. A further 31 articles were excluded following full text screening. Duplicate screening did not produce any discrepancies which could not be resolved through discussion. In total, 39 recruitment studies (21-58) were identified which reported results from 68 real host RCTs and 13 hypothetical host RCTs and were included in this review

For peer review only - http://bmjopen.bmj.com/site/about/guidelines.xhtml

Table 1: Number of included studies, host RCTs and hypothetical RCTs categorised according to the new recruitment study types (A to D)

Category	Recruitment study design	Recruitment studies (n=39)	Real host RCTs (n=68)	Hypothetical host RCTs (n=13)
**A**	Randomised controlled trials (RCTs) of interventions to optimise recruitment nested within one or more host RCTs	1	1	N/A

For peer review only

For peer review only - http://bmjopen.bmj.com/site/about/guidelines.xhtml

**B**	Prospectively designed, non-randomised studies of interventions to optimise recruitment to one or more host RCTs	3	3	N/A
**C**	Studies describing recruitment experiences involving one or more host RCTs	24	64*	N/A
**D**	Studies to consider recruitment within proposed hypothetical RCTs (commonly known as community consultations)	11	N/A	13**

RCT = randomised controlled trial, N/A = not applicable – category does not apply to type of host RCT

*The 24 recruitment studies reported data from 64 real host RCTs i.e. a number of recruitment studies reported data from more than one real host RCT

**The 11 recruitment studies reported data from 13 hypothetical studies i.e. two recruitment studies reported data from more than one hypothetical host RCT

For peer review only - http://bmjopen.bmj.com/site/about/guidelines.xhtml

(Figure 1). A number of recruitment studies described results obtained from more than one real or hypothetical RCT.

The 68 real host trials (around which recruitment was focused) were predominantly multi-centre RCTs (63/68) with large study populations (median = 624 participants, range = 4-58,050), and typically evaluated non-invasive medical interventions (61/68) (Table 2). The apparent predominance of RCTs in neurology is caused by one recruitment study which included data from 32 separate RCTs. With exception to this, the clinical settings of the host RCTs varied, encompassing several medical disciplines.

The majority of recruitment studies were simple descriptive non-randomised studies reporting previous experiences/challenges of recruitment to a host RCT (Category C, n =24). There were 11 that proposed a hypothetical RCT to a study population (Category D, n =11). Only one article used what is considered to be the optimal method for evaluating an intervention - a randomised comparison of a recruitment intervention nested within a host RCT (Category A). Three studies prospectively evaluated recruitment interventions using non-randomised study designs (Category B) (Table 1).

RCTs of interventions to optimise recruitment nested within one or more host RCTs (Category A)

Only one of the included recruitment studies investigated two recruitment strategies using a randomised design (Category A studies, Table 3). The rationale for this study was the limited time available for recruitment due the acute medical treatment required by patients. (21) Patients randomised to the intervention group received 'advanced notification' of the trial (via fax or phone) designed to offer patients more time to consider trial participation, compared to the control group who only received information once they met with the clinical team. Consent to participate in the host RCT was obtained in 27/50 (54%) and 25/50 (50%) patients in the intervention and control groups respectively (*P*= 0.69). Although no improvement in overall recruitment rates was demonstrated using advance notification, the provision of early information was demonstrated to be feasible.

For peer review only - http://bmjopen.bmj.com/site/about/guidelines.xhtml

Table 2: Summary characteristics of the host RCTs and hypothetical host RCTs in this review

Trial characteristic	Real RCTs (n = 68)	Hypothetical RCTs (n = 13)	Total (n=81)
**Clinical Setting**			
Neurology	39*	4	43
Cardiology	8	4	12
Obstetrics	3	1	4
Infection	3	0	3
Trauma	6	3	9
Critical care	9	1	10
**Type of interventions**			
Invasive / non-invasive	5 / 61	4 / 9	9 / 70
Unknown	2	0	2
**Trial design**			
Main RCT/ pilot RCT	65/3	n/a	65 / 3
Two groups / more than two groups	63 / 5	11 / 2	74 / 7
**Number of centres****			
Single centre / multi-centre / unknown	3 / 63 / 2	0 / 4 / 9	3 / 67 / 11
<20 centres	18		
20 -100 centres	33		
>100 centres	15		
Median number of centres (range)	45 (1 - 818)		
**Number of participants****			
<500 participants	22		
500 -1500 participants	32		
>1500 participants	14		
Median number of participants (range)	624 (4 - 58,050)		

*One recruitment paper included data from 32 Stroke host RCTs. RCT = randomised controlled trial

** Hypothetical RCT data did not include information beyond single or multicentre, nor the suggested no. of participants

For peer review only - http://bmjopen.bmj.com/site/about/guidelines.xhtml

Prospectively designed, non-randomised studies of interventions to optimise recruitment to one or more host RCTs (Category B)

The common rationale for this type of recruitment study design (Category B studies, Table 3) was the limitations of time when dealing with patients who required acute treatment. The need for urgent treatment was thought to hinder the ability of the study team to gain informed consent (IC) for trial participation.

The strategy of optimising information provision in the pre-hospital setting used in the one Category A study was mirrored by two Category B studies, which utilised the presence of pre-hospital staff to engage potential trial participants.(22, 24) Recruitment in these studies was reportedly optimised through provision of brief verbal information to participants as they travelled to hospital, and initial verbal consent that sought permission to deliver the emergency trial intervention. Further information was provided and written consent was subsequently obtained when the patients were stabilised, in hospital. One of these studies (24) also provided training to pre-hospital staff to improve their understanding of trial conduct, and devised a simple assessment of capacity to ensure that patients' initial verbal consent was valid.

The remaining prospectively designed (Category B) study focused on using technology to enhance recruitment during an influenza outbreak.(23) As patient numbers would be expected to rise rapidly across a wide geographical area, the study team devised a system which provided them with automated, real-time alerts whenever an eligible participant was identified in each hospital. This allowed one centralised study team to cover numerous study sites, enhancing recruitment opportunities. Although none of these studies provided numerical evidence of the effectiveness of their recruitment strategies, all authors concluded that their presented strategies were feasible and acceptable for use in UHA RCTs.

Studies describing recruitment experiences involving one or more host RCTs (Category C)

Rationales for reporting authors' experiences of recruitment were similar to those in prospectively designed studies, including the limited time available for consent (n=13), and recruitment difficulties caused by the clinical condition of the patients (n=18) (Category C studies, Table 4). Some studies were prompted by a host trial encountering recruitment difficulties (n=3). Of the 24 non-randomised studies describing recruitment experiences involving one or more host RCTs, 14 were observational and five were qualitative studies.

For peer review only - http://bmjopen.bmj.com/site/about/guidelines.xhtml

Table 3: Rationale and types of recruitment interventions reported in Category A or B studies (i.e. prospectively nested within RCTs)

Author and year	Rationale(s)	Descriptive summary of recruitment intervention(s)	Classification of recruitment intervention	Timing of information provision regarding host RCT	Timing of informed consent for host RCT	Timing of randomisation for host RCT
**Prospectively designed RCTs of interventions to optimise recruitment nested within one or more host RCTs (Category A studies)**						
Leira** 2009	Limited time for IC in host RCT due to the clinical condition requiring urgent treatment.	Intervention group:<br>'Advance notification' about host RCT using phone and fax prior to meeting pre-hospital team<br><br>Control group:<br>No information prior to meeting pre-hospital team	Advance notification using phone and fax	Intervention group:<br>'Advance notification' prior to face-to face meeting with pre-hospital team<br><br>Control group:<br>During face-to-face meeting with pre-hospital team	Intervention group:<br>During face-to-face meeting with pre-hospital team<br><br>Control group:<br>During face-to-face meeting with pre-hospital team	Intervention group:<br>Whilst patient in pre-hospital setting.<br><br>Control group:<br>Whilst patient in pre-hospital setting.
**Prospectively designed, non-randomised studies of interventions to optimise recruitment to one or more host RCTs (Category B studies)**						
Chow 2011	Recruitment team would be overwhelmed by covering multiple sites over a wide geographical area.	An automated service notified recruitment team in real time when a potentially eligible participant was identified across multiple centres	Automated service to facilitate real time notifications to recruitment team covering multiple sites	During face-to-face meeting with recruitment staff (once eligibility confirmed)	During face-to-face meeting with recruitment team	Whilst patient in hospital.
Shaw 2014	Limited time for IC in host RCT due to the clinical condition requiring urgent treatment.<br><br>Pre-hospital staff have limited experience of RCTs	Standardised verbal information was provided by pre-hospital staff before a simple capacity assessment, verbal consent and delivery of emergency treatment<br><br>Host RCT procedures were tailored towards pre-hospital staff, who also attended a one-day training event	Optimising information provision in pre-hospital setting.<br><br>Allowing verbal consent from patient or SDM.<br><br>Training of pre-hospital staff	Simple verbal information provided in pre-hospital setting.<br><br>Further full information provided in hospital	Initial verbal consent given in pre-hospital setting.<br><br>Written informed consent gained in hospital.	Whilst patient in pre-hospital setting.
Beshansky 2014	Limited time for IC in host RCT due to the clinical condition requiring urgent treatment.	Standardised verbal information was provided by pre-hospital staff before a simple capacity assessment, verbal consent and delivery of emergency treatment	Optimising information provision in pre-hospital setting.<br><br>Allowing verbal consent from patient.	Simple verbal information provided in pre-hospital setting.<br><br>Further full information provided in hospital	Initial verbal consent in pre-hospital setting.<br><br>Written informed consent in hospital.	Whilst patient in pre-hospital setting.

RCT = randomised controlled trial, IC= informed consent, SDM = surrogate decision maker

** Leira et al – patients were randomised to the *recruitment* intervention or control group prior to the recruitment team seeing the patient, and without consent from the participant.

For peer review only - http://bmjopen.bmj.com/site/about/guidelines.xhtml

Table 4: Frequency of rationales, study designs and recommendations from non-randomised studies describing recruitment experiences involving one or more host RCTs (i.e. Category C studies).

Recruitment study characteristic	Description	Frequency in Category C recruitment studies (n=24)**
**Rationale**	Patients too unwell to provide IC	18
	Limited time for IC in host RCT due to the clinical condition requiring urgent treatment	13
	Host RCT not meeting recruitment targets (at one or more sites) or terminated due to poor recruitment	3
	To better understand the impact of altering eligibility criteria on recruitment	2
	To better understand the impact of availability of SDMs on recruitment	2
	To better understand the recruitment process in a host RCT	1
	To better understand clinicians reasons for refusing patient participation in host RCT	1
**Recruitment study design**	Observational study of recruitment	14
	Qualitative studies of host participants/SDMs or PIS	5
	Survey of host RCT participants	2
	Survey of clinical staff involved in host RCT	1
	Simulation study evaluating the effect of altering eligibility criteria in multiple host RCTs	1
	Meta-analysis of recruitment data in host RCTs	1
**Recommendations for optimising recruitment in future RCTs or areas for further research into recruitment*** **	To provide RCT information verbally and allow a verbal consent process	10
	To use a screening log can to provide insight into recruitment difficulties	8
	Patients or SDM were unable to recall key RCT information after providing IC***	5
	To use a 'waiver of consent'/ 'deferred consent'/ 'EFIC'	4
	To perform regular site visits	3
	To use a broad eligibility criteria /broad therapeutic window	2
	To use SDMs	2
	Novel methods for obtaining IC are required***	2
	To replace poorly recruiting centres	1
	To approach more eligible patients	1
	To survey staff involved with host RCT to provide insight into recruitment difficulties	1

RCT = randomised controlled trial, IC= informed consent, EFIC = exception from informed consent, SDM = surrogate decision maker, PIS= patient information sheet.
**each study may appear more than once in the relevant characteristics section (e.g. if it described more >1 rationale, or produced >1 finding/recommendation)
*** items for further research and not recommendations for optimising recruitment

For peer review only - http://bmjopen.bmj.com/site/about/guidelines.xhtml

These studies reported experiences of trial participants and surrogate decision makers (SDMs), or extracted verbatim information from written patient information sheets (PISs). Recommendations for optimising recruitment also mirrored Category A and B studies, highlighting the acceptability of verbal information provision and consent, or deferring consent altogether until an unwell patient is suitably stabilised. Additional benefits were seen in RCTs which used data from screening logs to uncover recruitment barriers, and trials which performed regular site visits.(29-31, 33, 35, 38, 39, 44) A sub section of qualitative studies, although not presenting recommendations for future trial conduct, highlighted their findings that many patients or SDMs who had provided consent to participate in an RCT did not recall much of the information provided to them during the consent process, suggesting that work was needed to improve consent in this setting.(31, 32, 40)

Studies to consider recruitment within proposed hypothetical RCTs (commonly known as community consultations) (Category D)

Eleven studies reported community views about proposed 'hypothetical' RCTs (Category D studies, Table 5). Although the rationales for conducting the studies were similar to studies involving real host RCTs (unwell patients and lack of time for consent), the study designs were varied including questionnaire surveys, interviews, and focus group meetings. Verbal information provision, verbal consent, and recruitment in the pre-hospital setting were identified as helpful recruitment strategies in these studies. However, these articles raised new issues around the appropriateness of using of SDMs when patients are too unwell to provide consent for themselves, and raised additional issues around who the SDM should be (next of kin (NOK), or an available physician). Some studies found a preference for the use of SDMs,(50, 52, 56) while others expressed that NOK or physicians should not be used for their specific hypothetical RCTs.(53, 55, 58)

Discussion

This review aimed to examine and summarise studies and methods used to optimise recruitment in RCTs in patients with an unscheduled hospital admission (UHA). It had the purpose of using the information to consider how to optimise recruitment in this challenging clinical setting in future studies. In the ORCCA database of recruitment research, only 39 out of a possible 3114 articles (1.25%) focused on recruitment to RCTs in the UHA setting. Only one of these studies was a randomised comparison of recruitment strategies; the majority of

For peer review only - http://bmjopen.bmj.com/site/about/guidelines.xhtml
For peer review only

Table 5: Frequency of rationales, study designs, main findings and recommendations from Non-randomised studies designed study to consider recruitment within proposed hypothetical RCTs (i.e. Category D studies, commonly known as community consultations).

Recruitment study characteristic	Description	Frequency in Category D recruitment studies (n=11)**
**Rationale**	Patients too unwell to provide IC	9
	Limited time for IC in host RCT due to the clinical condition requiring urgent treatment	6
	To explore the accuracy of decisions made by NOK when acting as SDM	1
**Recruitment study design**	Questionnaire survey	5
	Face-to-face interview	4
	Telephone survey	1
	Focus group meetings	1
**Recommendations for optimising recruitment in future RCTs or main findings*****	To use a physician as a SDM	4
	To use NOK as a SDM	4
	To use EFIC	3
	**Not** to use NOK as a SDM	2
	To provide RCT information verbally and allow a verbal consent process	1
	To allow recruitment in pre-hospital setting	1
	To perform community consultations to estimate host RCT recruitment rates***	1
	**Not** to use a physician as a SDM	1
	**Not** to use EFIC	1
	To perform community consultations to aid selection of relevant study outcomes***	1

*RCT = randomised controlled trial, EFIC = exception from informed consent, NOK = next of kin, SDM = surrogate decision maker
**each study may appear more than once in the relevant characteristics section (e.g. if it described more >1 rationale, or produced >1 finding/recommendation)
*** items reported as main findings, but not recommendations for optimising recruitment

For peer review only - http://bmjopen.bmj.com/site/about/guidelines.xhtml

studies consisted of simple study designs describing recruitment experiences. Eleven further studies involved hypothetical RCTs, and whilst of some value, it is uncertain how these types of investigations translate into optimal RCT design. This work therefore highlights the need for development of interventions to optimise recruitment in the UHA setting and prospective evaluation of their effectiveness and acceptability.

Comparison to existing literature

There have been several reviews that have summarised the evidence for optimising recruitment in trials in other clinical conditions or contexts, including cancer (4-6), geriatrics (8, 9), primary care (7), and a mix of clinical settings.(11-17). Similar to our findings, these reviews have commonly highlighted the lack of high quality evaluations of recruitment interventions (6,16). Despite this, some of the reviews have identified effective recruitment strategies, although these vary in the extent to which they are likely to be transferrable to the UHA setting. For example, it is unclear if interventions such as telephone reminders (16,17), 'education sessions' about the health condition (11), and use of monetary incentives (11,17) are as effective or appropriate in the UHA context, given the specific factors that may compromise recruitment in this setting (e.g. patients in pain/distress, short timeframes for recruitment, busy settings, etc.). Other reviews have drawn attention to interventions aimed at recruiters, such as appropriate training/guidance (12), reduction of clinical workload (7,12), and 'research protected' time (12). These have potential to be helpful in trials conducted in the UHA setting, although further research is needed to examine the content/nature of the training materials needed, and the logistics of implementing these types of interventions. Finally, some reviews have shown that features of RCT study design – such as open, rather than placebo-controlled trials - are associated with better recruitment outcomes (16,17). Recommendations that advise against particular study designs may limit the quality of evidence generated to guide future patient care, and dissuade from the most important (and appropriate) clinical questions from being addressed. Furthermore, there is a growing body of evidence to indicate that it is possible to recruit to more complex RCTs with appropriate training and support.(59) More generally, use of integrated qualitative research to understand and address recruitment difficulties is being increasingly recommended in more recent reviews (12)(60), and is likely to inform novel insights if applied to trials in UHA settings.

For peer review only - http://bmjopen.bmj.com/site/about/guidelines.xhtml

Some reviews and individual studies have produced recommendations that are likely to be particularly relevant for developing UHA-specific recruitment strategies for future evaluation. These strategies may tackle some of the context-specific difficulties that are likely to be experienced in UHA settings. For example, one systematic review focusing on recruitment to RCTs involving patients with cancer or organ failure highlighted the potential for providing audio-visual information (such as a video to explain the RCT) to facilitate RCT recruitment (59). Such an approach may be helpful in the UHA setting, given that that large amounts of written information may not be appropriate in patients who are in pain or feeling distressed. This recommendation is also in keeping with guidance issued by the NHS Health Research Authority (HRA), which suggests using alternatives to written information (61).

A verbal exchange of information and providing initial verbal consent has been previously suggested as a preferred strategy to written alternatives in RCT taking place in the emergency setting (62). It is agreed that for UHAs this could be preferable, although it is considered that further work is needed to develop this type of verbal consent to ensure that quality assurance is still achieved for consent. Another suggested solution to this problem is the use of an independent patient advocate, who may oversee such conversations between trial team members and acutely unwell patients, to verify that appropriate information exchange took place, and to act as an assessor of a patient's willingness to participate in the RCT.(63) This strategy provides one potential solution to a significant obstacle in recruitment of patients undergoing an UHA. However, it may be practically difficult to achieve given that UHAs can occur at any time of the day or night and such trained patient advocates would also need to be available during these times making a trial more expensive.

Finally, a non-systematic review article focusing on recruitment to emergency medicine research also highlighted a similar problem in a lack of high quality evidence on recruitment. It suggested support for the use of deferred consent (also known as exception from informed consent) which was highlighted in this review and additionally raised the issue that the use of SDMs for consent may be problematic due to the pressures of time and the emotional stress family members will be under while a relative is acutely unwell.(3)

Strengths and weaknesses of this study

This review is the first to systematically focus on recruitment strategies in the UHA setting. With a reported growing number of unscheduled hospital admissions presenting great

For peer review only - http://bmjopen.bmj.com/site/about/guidelines.xhtml

challenges to modern-day health care provision, the conduct of RCTs in the UHA will inevitably develop as an area of research.

The review may be limited through its reliance on a single search of the ORRCA database, conducted in February 2016, at which point the database contained publications relevant to recruitment published up to the end of December 2014. The ORRCA database continues to evolve as updates encompass newly published recruitment research. Updates to the database may have generated further UHA research articles since the search for this review. It is possible that any new update could make an important and significant contribution to this field because so little has been done in this area thus far.

The review may be limited because a single researcher reviewed the majority of the ORRCA entries, and it is possible that ORRCA may not have included all relevant articles in the first place. This work may also be at risk of publication bias, as we chose to exclude abstracts based on the assumption that these were unlikely to include all the data items we were interested in. Excluding abstracts may have resulted in omission of some potentially valuable information. However, there is some evidence to suggest that there is discordance between the content of abstracts and the subsequent full-text publication, and as such, including abstracts may have introduced unreliable data. (64, 65) Another weakness is that the majority of recruitment studies retrospective analyses of processes and events that occurred during the host RCT. These data were not necessarily collected with the intention of evaluating RCT recruitment strategies. This may limit the quality of the data and the utility of the recommendations arising from the included studies.

Finally, a risk of bias assessment of the included recruitment studies was not performed, because only one of the recruitment studies was an RCT (i.e. a randomised, controlled evaluation of a recruitment intervention).

Unanswered questions and future research

Some articles within this review demonstrated inconsistent conclusions about the value of surrogate decision makers, who these should be, and how these should operate. Future research should examine these issues in more depth, in a variety of clinical contexts, focusing on the roles of SDM in different RCTs and to what extent the public it acceptable for an SDM to decide on their behalf for trials involving acutely unwell patients.

For peer review only - http://bmjopen.bmj.com/site/about/guidelines.xhtml
For peer review only

It is uncertain how well the results of studies using hypothetical RCTs can be translated to the conduct of real RCTs. There may be some validity in the findings from hypothetical RCTs, as highlighted in this review by the similarity of the results from studies which used real RCTs and hypothetical RCTs. Further work is required to ascertain the extent to which findings from studies which use hypothetical RCTs reliably influence the design and conduct of real RCTs.

As part of this review a classification system was devised in order to group together similarly designed recruitment research studies. Before any such classification system could be used more widely, it would require validation by testing its applicability to at least one further set of recruitment papers, preferably in a different health care setting.

This review highlighted strategies to deal with patients in the pre-hospital setting who required urgent treatment. However, not all patients who are cared for in the pre-hospital setting require treatment immediately. Some may require transport to hospital for further assessment and potential treatment. Further research could explore whether providing early trial information to such patients based on their symptoms or presumed diagnosis, could affect trial recruitment, should treatment be required later. This strategy could prove to be useful in a broader range of unscheduled hospital admissions.

Although some recommendations for optimising recruitment could be drawn from this review, the overall lack of research in this area, particularly amongst high quality, methodologically robust studies, is a limiting factor. Future recruitment studies in this clinical setting should focus on studies with higher methodological rigour, by developing novel interventions to optimise recruitment and prospectively evaluating their effectiveness through an appropriate study design.

Conclusion

There is a relative paucity of high quality research on strategies to optimise recruitment to RCTs involving unscheduled hospital admissions. Some emerging recommendations include optimising information provision about the trial in the pre-hospital setting to improve recruitment where treatment is required urgently, or using technology to facilitate

For peer review only - http://bmjopen.bmj.com/site/about/guidelines.xhtml

recruitment across many hospital sites. Screening log data can also provide useful insight to specific barriers to recruitment. Future research in this setting should focus on conducting studies with higher methodological rigour, by developing interventions to optimise recruitment and prospectively evaluating their effectiveness.

Acknowledgements

The following are gratefully acknowledged for their assistance and support with this work: Shaun Treweek, Anna Kearney, Nicola Harman.

Funding

CR is supported by the Medical Research Council (MRC) Hub for Trials Methodology Research Network and the Collaboration and Innovation for Difficult Trials in Invasive procedures (ConDuCT-II) Hub for Trials Methodology Research (MR/K025643/1). The funders had no role in study design, data collection and analysis, decision to publish, or preparation of manuscript.

Contributor statement

CR, LR JR, and JMB designed the study and formulated the protocol. CR and KF performed all data extraction, which was reviewed and critically analysed by CR, LR and JMB. CG facilitated access to the ORRCA database and critically appraised the review. CR produced the first draft of the manuscript and LR and JMB made substantial contributions to the formulation of the final version. All authors read and approved the final manuscript.

Competing interests

No competing interests

Data sharing statement

As this is a review of published literature, there are no additional data available.

Figure 1: Study selection PRISMA flow diagram

References

1. Tudur Smith C, Hickey H, Clarke M, Blazeby J, Williamson P. The trials methodological research agenda: results from a priority setting exercise. Trials. 2014;15:32-.

For peer review only - http://bmjopen.bmj.com/site/about/guidelines.xhtml

2. Schandelmaier S, von Elm E, You JJ, Blumle A, Tomonaga Y, Lamontagne F, et al. Premature Discontinuation of Randomized Trials in Critical and Emergency Care: A Retrospective Cohort Study. Critical care medicine. 2016;44(1):130-7.
3. Cofield SS, Conwit R, Barsan W, Quinn J. Recruitment and retention of patients into emergency medicine clinical trials. Academic emergency medicine : official journal of the Society for Academic Emergency Medicine. 2010;17(10):1104-12.
4. Boland J, Currow DC, Wilcock A, Tieman J, Hussain JA, Pitsillides C, et al. A systematic review of strategies used to increase recruitment of people with cancer or organ failure into clinical trials: implications for palliative care research. Journal of pain and symptom management. 2015;49(4):762-72.e5.
5. Fayter D, McDaid C, Eastwood A. A systematic review highlights threats to validity in studies of barriers to cancer trial participation. Journal of clinical epidemiology. 2007;60(10):990-1001.
6. Mc Daid C, Hodges Z, Fayter D, Stirk L, Eastwood A. Increasing participation of cancer patients in randomised controlled trials: a systematic review. Trials. 2006;7:16.
7. Ngune I, Jiwa M, Dadich A, Lotriet J, Sriram D. Effective recruitment strategies in primary care research: a systematic review. Quality in primary care. 2012;20(2):115-23.
8. Auster J, Janda M. Recruiting older adults to health research studies: A systematic review. Australasian journal on ageing. 2009;28(3):149-51.
9. Provencher V, Mortenson WB, Tanguay-Garneau L, Belanger K, Dagenais M. Challenges and strategies pertaining to recruitment and retention of frail elderly in research studies: a systematic review. Archives of gerontology and geriatrics. 2014;59(1):18-24.
10. Ibrahim S, Sidani S. Strategies to recruit minority persons: a systematic review. Journal of immigrant and minority health / Center for Minority Public Health. 2014;16(5):882-8.
11. Caldwell PH, Hamilton S, Tan A, Craig JC. Strategies for increasing recruitment to randomised controlled trials: systematic review. PLoS medicine. 2010;7(11):e1000368.
12. Fletcher B, Gheorghe A, Moore D, Wilson S, Damery S. Improving the recruitment activity of clinicians in randomised controlled trials: a systematic review. BMJ open. 2012;2(1):e000496.
13. Huynh L, Johns B, Liu SH, Vedula SS, Li T, Puhan MA. Cost-effectiveness of health research study participant recruitment strategies: a systematic review. Clinical trials (London, England). 2014;11(5):576-83.
14. Mapstone J, Elbourne D, Roberts I. Strategies to improve recruitment to research studies. The Cochrane database of systematic reviews. 2007(2):Mr000013.
15. Raftery J, Bryant J, Powell J, Kerr C, Hawker S. Payment to healthcare professionals for patient recruitment to trials: systematic review and qualitative study. Health technology assessment (Winchester, England). 2008;12(10):1-128, iii.
16. Treweek S, Mitchell E, Pitkethly M, Cook J, Kjeldstrom M, Taskila T, et al. Strategies to improve recruitment to randomised controlled trials. The Cochrane database of systematic reviews. 2010(1):Mr000013.

For peer review only

For peer review only - http://bmjopen.bmj.com/site/about/guidelines.xhtml

17. Watson JM, Torgerson DJ. Increasing recruitment to randomised trials: a review of randomised controlled trials. BMC medical research methodology. 2006;6:34.
18. Burns KE, Prats CJ, Maione M, Lanceta M, Zubrinich C, Jeffs L, et al. The Experience of Surrogate Decision Makers on Being Approached for Consent for Patient Participation in Research. A Multicenter Study. Annals of the American Thoracic Society. 2017;14(2):238-45.
19. Whitesides LW, Baren JM, Biros MH, Fleischman RJ, Govindarajan PR, Jones EB, et al. Impact of individual clinical outcomes on trial participants' perspectives on enrollment in emergency research without consent. Clinical trials (London, England). 2017;14(2):180-6.
20. Fordyce CB, Roe MT, Dickert NW. Maximizing value and minimizing barriers: Patient-centered community consultation for research in emergency settings. Clinical trials (London, England). 2017;14(1):88-93.
21. Leira EC, Ahmed A, Lamb DL, Olalde HM, Callison CR, Torner JC, et al. Extending Acute Trials to Remote Populations A Pilot Study During Interhospital Helicopter Transfer. Stroke. 2009;40(3):895-901.
22. Beshansky JR, Sheehan PR, Klima KJ, Hadar N, Vickery EM, Selker HP. A community consultation survey to evaluate support for and success of the IMMEDIATE trial. Clinical Trials. 2014;11(2):178-86.
23. Chow E, Zuberi M, Seto R, Hota S, Fish EN, Morra D. Using real-time alerts for clinical trials Identifying potential study subjects. Applied Clinical Informatics. 2011;2(4):472-80.
24. Shaw L, Price C, McLure S, Howel D, McColl E, Younger P, et al. Paramedic Initiated Lisinopril For Acute Stroke Treatment (PIL-FAST): results from the pilot randomised controlled trial. Emerg Med J. 2014;31(12):994-9.
25. Abramson NS, Safar P. Deferred consent: use in clinical resuscitation research. Brain Resuscitation Clinical Trial II Study Group. Annals of Emergency Medicine. 1990;19(7):781-4.
26. Adeoye O, Pancioli A, Khoury J, Moomaw CJ, Schmit P, Ewing I, et al. Efficiency of Enrollment in a Successful Phase II Acute Stroke Clinical Trial. Journal of Stroke & Cerebrovascular Diseases. 2012;21(8):667-72.
27. Ågård A, Hermerén G, Herlitz J. Patients' experiences of intervention trials on the treatment of myocardial infarction: Is it time to adjust the informed consent procedure to the patient's capacity? Heart. 2001;86(6):632-7.
28. Annane D, Outin H, Fisch C, Bellissant E. The effect of waiving consent on enrollment in a sepsis trial. Intensive Care Medicine. 2004;30(2):321-4.
29. Bellomo R, Trial RRT. Screening and Study Enrolment in the Randomized Evaluation of Normal vs. Augmented Level (RENAL) Replacement Therapy Trial. Blood Purification. 2009;27(2):199-205.
30. Burns KEA, Zubrinich C, Tan WL, Raptis S, Xiong W, Smith O, et al. Research Recruitment Practices and Critically Ill Patients A Multicenter, Cross-Sectional Study (The Consent Study). American Journal of Respiratory and Critical Care Medicine. 2013;187(11):1212-8.
31. Chlan L, Guttormson J, Tracy MF, Bremer KL. STRATEGIES FOR OVERCOMING SITE AND RECRUITMENT CHALLENGES IN RESEARCH STUDIES BASED IN INTENSIVE CARE UNITS. American Journal of Critical Care. 2009;18(5):410-7.

For peer review only - http://bmjopen.bmj.com/site/about/guidelines.xhtml

32. Collins JF. Data and safety monitoring board issues raised in the VA Status Epilepticus Study. Controlled Clinical Trials. 2003;24(1):71-7.
33. Cook D, Arabi Y, Ferguson ND, Heels-Ansdell D, Freitag A, McDonald E, et al. Physicians declining patient enrollment in a critical care trial: a case study in thromboprophylaxis. Intensive Care Medicine. 2013;39(12):2115-25.
34. Costescu DJW, Cullimore AJ. Lessons learned from a resident-led clinical trial in obstetrics. Clinical Trials. 2013;10(4):612-6.
35. Crowley ST, Chertow GM, Vitale J, O'Connor T, Zhang JN, Schein RMH, et al. Lessons for successful study enrollment from the Veterans Affairs/National Institutes of Health Acute Renal Failure Trial Network Study. Clinical Journal of the American Society of Nephrology. 2008;3(4):955-61.
36. Elkins JS, Khatabi T, Fung L, Rootenberg J, Johnston SC. Recruiting subjects for acute stroke trials: a meta-analysis. Stroke [Internet]. 2006; 37(1):[123-8 pp.]. Available from: http://onlinelibrary.wiley.com/o/cochrane/clcmr/articles/CMR-10639/frame.html
http://stroke.ahajournals.org/content/37/1/123.full.pdf.
37. Flaherty ML, Karlawish J, Khoury JC, Kleindorfer D, Woo D, Broderick JP. How important is surrogate consent for stroke research? Neurology. 2008;71(20):1566-71.
38. Foster D, Cook D, Granton J, Steinberg M, Marshall J. Use of a screen log to audit patient recruitment into multiple randomized trials in the intensive care unit. Canadian Critical Care Trials Group. Critical care medicine [Internet]. 2000; 28(3):[867-71 pp.]. Available from:
http://onlinelibrary.wiley.com/o/cochrane/clcmr/articles/CMR-11625/frame.html.
39. Glassberg AE, Luce JM, Matthay MA. Reasons for nonenrollment in a clinical trial of acute lung injury. Chest [Internet]. 2008; 134(4):[719-23 pp.]. Available from:
http://onlinelibrary.wiley.com/o/cochrane/clcmr/articles/CMR-12450/frame.html
http://journal.publications.chestnet.org/data/Journals/CHEST/22076/zcb01008000719.pdf.
40. Kendall B, Stadeli R, Schegg B, Olbrich M, Chen E, Harmelin-Kadouri R, et al. Clinical Trial Educator program - a novel approach to accelerate enrollment in a phase III International Acute Coronary Syndrome Trial. Clinical Trials. 2012;9(3):358-66.
41. Kenyon S, Dixon-Woods M, Jackson CJ, Windridge K, Pitchforth E. Participating in a trial in a critical situation: a qualitative study in pregnancy. Quality & Safety in Health Care. 2006;15(2):98-101.
42. Roozenbeek B, Maas AIR, Marmarou A, Butcher I, Lingsma HF, Lu J, et al. The influence of enrollment criteria on recruitment and outcome distribution in traumatic brain injury studies: results from the impact study. Journal of Neurotrauma [Internet]. 2009; 26(7):[1069-75 pp.]. Available from:
http://onlinelibrary.wiley.com/o/cochrane/clcmr/articles/CMR-14708/frame.html
http://online.liebertpub.com/doi/pdfplus/10.1089/neu.2008.0569.
43. Schats R, Brilstra EH, Rinkel GJ, Algra A, Gijn J. Informed consent in trials for neurological emergencies: the example of subarachnoid haemorrhage. Journal of Neurology, Neurosurgery, and Psychiatry [Internet]. 2003; 74(7):[988-91 pp.]. Available from: http://onlinelibrary.wiley.com/o/cochrane/clcmr/articles/CMR-12831/frame.html
http://www.ncbi.nlm.nih.gov/pmc/articles/PMC1738516/pdf/v074p00988.pdf.

For peer review only - http://bmjopen.bmj.com/site/about/guidelines.xhtml

44. Smith OM, McDonald E, Zytaruk N, Foster D, Matte A, Clarke F, et al. Rates and determinants of informed consent: A case study of an international thromboprophylaxis trial. Journal of Critical Care. 2013;28(1):28-39.
45. Smyth RM, Jacoby A, Elbourne D. Deciding to join a perinatal randomised controlled trial: experiences and views of pregnant women enroled in the Magpie Trial. Midwifery. 2012;28(4):E478-85.
46. Williams BF, French JK, White HD. Informed consent during the clinical emergency of acute myocardial infarction (HERO-2 consent substudy): A prospective observational study. Lancet. 2003;361(9361):918-22.
47. Yamal JM, Robertson CS, Rubin ML, Benoit JS, Hannay HJ, Tilley BC. Enrollment of racially/ethnically diverse participants in traumatic brain injury trials: Effect of availability of exception from informed consent. Clinical Trials. 2014;11(2):187-94.
48. Yuval R, Halon DA, Merdler A, Khader N, Karkabi B, Uziel K, et al. Patient comprehension and reaction to participating in a double-blind randomized clinical trial (ISIS-4) in acute myocardial infarction. Archives of Internal Medicine. 2000;160(8):1142-6.
49. Abboud PA, Heard K, Al-Marshad AA, Lowenstein SR. What determines whether patients are willing to participate in resuscitation studies requiring exception from informed consent? Journal of Medical Ethics [Internet]. 2006; 32(8):[468-72 pp.]. Available from: http://onlinelibrary.wiley.com/o/cochrane/clcmr/articles/CMR-10612/frame.html.
50. Ali K, Roffe C, Crome P. What patients want: consumer involvement in the design of a randomized controlled trial of routine oxygen supplementation after acute stroke. Stroke [Internet]. 2006; 37(3):[865-71 pp.]. Available from: http://onlinelibrary.wiley.com/o/cochrane/clcmr/articles/CMR-10638/frame.html
http://stroke.ahajournals.org/content/37/3/865.full.pdf.
51. Bulger EM, Schmidt TA, Cook AJ, Brasel KJ, Griffiths DE, Kudenchuk PJ, et al. The Random Dialing Survey as a tool for community consultation for research involving the emergency medicine exception from informed consent. Annals of Emergency Medicine [Internet]. 2009; 53(3):[341-50 pp.]. Available from: http://onlinelibrary.wiley.com/o/cochrane/clcmr/articles/CMR-14468/frame.html
http://ac.els-cdn.com/S0196064408015126/1-s2.0-S0196064408015126-main.pdf?_tid=3127512a-62ab-11e5-948a-00000aab0f27&acdnat=1443092490_2b79f56df4905bd0b2acd045224491cf.
52. Clark DJ, Kolias AG, Corteen EA, Ingham SC, Piercy J, Crick SJ, et al. Community consultation in emergency neurotrauma research: results from a pre-protocol survey. Acta Neurochirurgica. 2013;155(7):1329-34; discussion 34.
53. Giudice AD, Plaum J, Maloney E, Kasner SE, Le Roux PD, Baren JM. Who will consent to emergency treatment trials for subarachnoid hemorrhage? Academic Emergency Medicine. 2009;16(4):309-15.
54. Goldstein JN, Espinola JA, Fisher J, Pallin DJ, Camargo Jr CA. Public opinion of a stroke clinical trial using exception from informed consent. International Journal of Emergency Medicine. 2010;3(4):385-9.
55. Newman JT, Smart A, Reese TR, Williams A, Moss M. Surrogate and patient discrepancy regarding consent for critical care research. Critical care medicine. 2012;40(9):2590-4.

For peer review only - http://bmjopen.bmj.com/site/about/guidelines.xhtml

56. Scotton WJ, Kolias AG, Ban VS, Crick SJ, Sinha R, Gardner A, et al. Community consultation in emergency neurosurgical research: lessons from a proposed trial for patients with chronic subdural haematomas. British Journal of Neurosurgery. 2013;27(5):590-4.
57. Sims CA, Isserman JA, Holena D, Sundaram LM, Tolstoy N, Greer S, et al. Exception from informed consent for emergency research: Consulting the trauma community. Journal of Trauma and Acute Care Surgery. 2013;74(1):157-66.
58. Snowdon C, Elbourne D, Forsey M, Alfirevic Z. Views of emergency research (VERA): A qualitative study of women and their partners' views of recruitment to trials in severe postpartum haemorrhage. Midwifery. 2012;28(6):800-8.
59. Donovan JL, Rooshenas L, Jepson M, Elliott D, Wade J, Avery K, et al. Optimising recruitment and informed consent in randomised controlled trials: the development and implementation of the Quintet Recruitment Intervention (QRI). Trials. 2016;17(1):283.
60. Elliott D, Husbands S, Hamdy FC, Holmberg L, Donovan JL. Understanding and Improving Recruitment to Randomised Controlled Trials: Qualitative Research Approaches. Eur Urol. 2017.
61. Authority NHR. Applying a proportionate approach to the process of seeking consent - HRA Guidance. 2017.
62. Roberts I, Prieto-Merino D, Shakur H, Chalmers I, Nicholl J. Effect of consent rituals on mortality in emergency care research. Lancet. 2011;377(9771):1071-2.
63. Sahan KM, Channon KM, Choudhury RP, Kharbanda RK, Lee R, Sheehan M. Refining the Enrolment Process in Emergency Medicine Research. Eur J Cardiovasc Med. 2016;4(1):506-10.
64. Tam VC, Hotte SJ. Consistency of phase III clinical trial abstracts presented at an annual meeting of the American Society of Clinical Oncology compared with their subsequent full-text publications. J Clin Oncol. 2008;26(13):2205-11.
65. Toma M, McAlister FA, Bialy L, Adams D, Vandermeer B, Armstrong PW. Transition from meeting abstract to full-length journal article for randomized controlled trials. JAMA. 2006;295(11):1281-7.

For peer review only

For peer review only - http://bmjopen.bmj.com/site/about/guidelines.xhtml

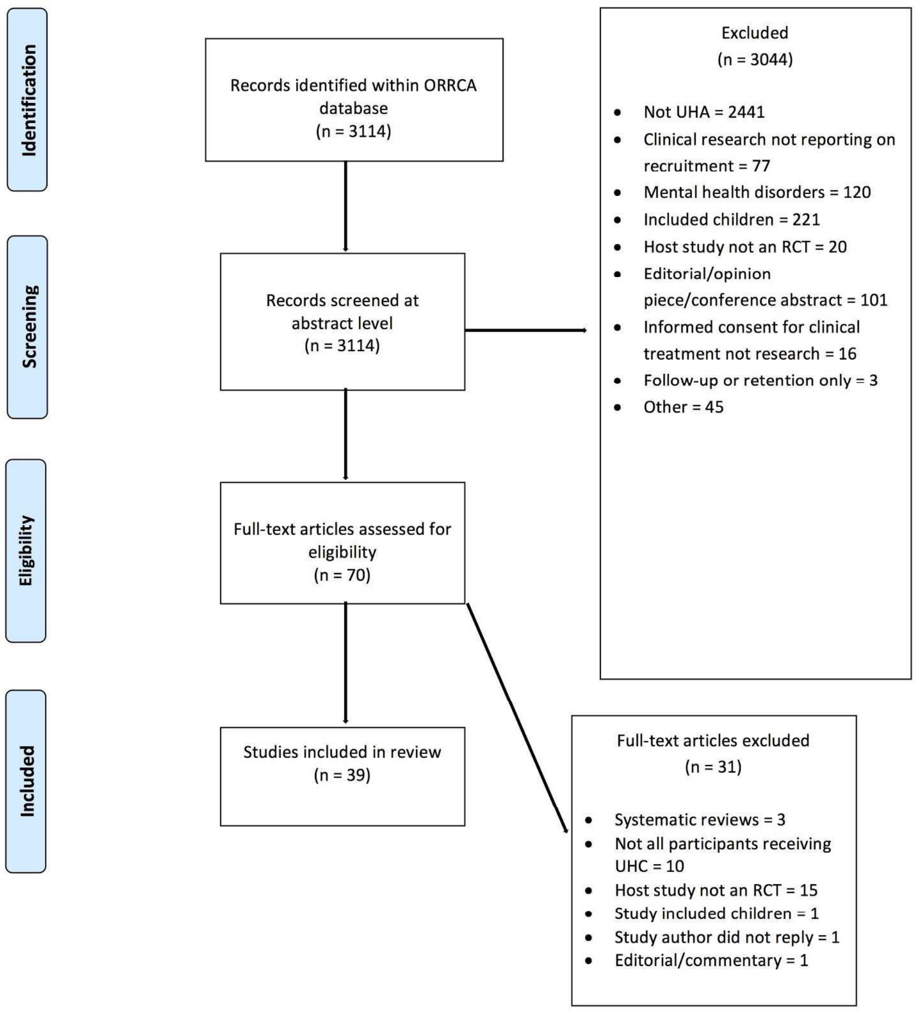

Figure 1: Study selection PRISMA flow diagram

208x242mm (300 x 300 DPI)

	#	Checklist item	Reported on page #
**TITLE**			
Title	1	Identify the report as a systematic review, meta-analysis, or both.	1
**ABSTRACT**			
Structured summary	2	Provide a structured summary including, as applicable: background; objectives; data sources; study eligibility criteria, participants, and interventions; study appraisal and synthesis methods; results; limitations; conclusions and implications of key findings; systematic review registration number.	2
**INTRODUCTION**			
Rationale	3	Describe the rationale for the review in the context of what is already known.	3
Objectives	4	Provide an explicit statement of questions being addressed with reference to participants, interventions, comparisons, outcomes, and study design (PICOS).	5
**METHODS**			
Protocol and registration	5	Indicate if a review protocol exists, if and where it can be accessed (e.g., Web address), and, if available, provide registration information including registration number.	N/A
Eligibility criteria	6	Specify study characteristics (e.g., PICOS, length of follow-up) and report characteristics (e.g., years considered, language, publication status) used as criteria for eligibility, giving rationale.	5-7
Information sources	7	Describe all information sources (e.g., databases with dates of coverage, contact with study authors to identify additional studies) in the search and date last searched.	4-5
Search	8	Present full electronic search strategy for at least one database, including any limits used, such that it could be repeated.	4-5
Study selection	9	State the process for selecting studies (i.e., screening, eligibility, included in systematic review, and, if applicable, included in the meta-analysis).	5-6

For peer review only

Data collection process	10	Describe method of data extraction from reports (e.g., piloted forms, independently, in duplicate) and any processes for obtaining and confirming data from investigators.	7
Data items	11	List and define all variables for which data were sought (e.g., PICOS, funding sources) and any assumptions and simplifications made.	7
Risk of bias in individual studies	12	Describe methods used for assessing risk of bias of individual studies (including specification of whether this was done at the study or outcome level), and how this information is to be used in any data synthesis.	N/A
Summary measures	13	State the principal summary measures (e.g., risk ratio, difference in means).	N/A
Synthesis of results	14	Describe the methods of handling data and combining results of studies, if done, including measures of consistency (e.g., I^2) for each meta-analysis.	N/A

Section/topic	#	Checklist item	Reported on page #
Risk of bias across studies	15	Specify any assessment of risk of bias that may affect the cumulative evidence (e.g., publication bias, selective reporting within studies).	N/A
Additional analyses	16	Describe methods of additional analyses (e.g., sensitivity or subgroup analyses, meta-regression), if done, indicating which were pre-specified.	N/A
**RESULTS**			
Study selection	17	Give numbers of studies screened, assessed for eligibility, and included in the review, with reasons for exclusions at each stage, ideally with a flow diagram.	7
Study characteristics	18	For each study, present characteristics for which data were extracted (e.g., study size, PICOS, follow-up period) and provide the citations.	8-10
Risk of bias within studies	19	Present data on risk of bias of each study and, if available, any outcome level assessment (see item 12).	N/A
Results of individual studies	20	For all outcomes considered (benefits or harms), present, for each study: (a) simple summary data for each intervention group (b) effect estimates and confidence intervals, ideally with a forest plot.	8-10

For peer review only

For peer review only - http://bmjopen.bmj.com/site/about/guidelines.xhtml

Synthesis of results	21	Present results of each meta-analysis done, including confidence intervals and measures of consistency.	N/A
Risk of bias across studies	22	Present results of any assessment of risk of bias across studies (see Item 15).	N/A
Additional analysis	23	Give results of additional analyses, if done (e.g., sensitivity or subgroup analyses, meta-regression [see Item 16]).	N/A
**DISCUSSION**			
Summary of evidence	24	Summarize the main findings including the strength of evidence for each main outcome; consider their relevance to key groups (e.g., healthcare providers, users, and policy makers).	10
Limitations	25	Discuss limitations at study and outcome level (e.g., risk of bias), and at review-level (e.g., incomplete retrieval of identified research, reporting bias).	11-12
Conclusions	26	Provide a general interpretation of the results in the context of other evidence, and implications for future research.	13
**FUNDING**			
Funding	27	Describe sources of funding for the systematic review and other support (e.g., supply of data); role of funders for the systematic review.	13-14

From: Moher D, Liberati A, Tetzlaff J, Altman DG, The PRISMA Group (2009). Preferred Reporting Items for Systematic Reviews and Meta-Analyses: The PRISMA Statement. PLoS Med 6(7): e1000097. doi:10.1371/journal.pmed1000097 N/A= not applicable

For more information, visit: **www.prisma-statement.org**.

For peer review only

For peer review only

BMJ Open

A detailed systematic analysis of recruitment strategies in randomised controlled trials in patients with an unscheduled admission to hospital

Journal:	*BMJ Open*
Manuscript ID	bmjopen-2017-018581.R3
Article Type:	Research
Date Submitted by the Author:	05-Dec-2017
Complete List of Authors:	Rowlands, Ceri; MRC ConDuCT-II Hub for Trials Methodology Research, School of Social & Community Medicine<br>Rooshenas, Leila; MRC ConDuCT-II Hub for Trials Methodology Research, School of Social & Community Medicine, University of Bristol<br>Fairhurst, Katherine; MRC ConDuCT-II Hub for Trials Methodology Research, School of Social & Community Medicine<br>Rees, Jonathan; University Hospitals Bristol NHS Foundation Trust, Division of Surgery, Head & Neck<br>Gamble, Carrol; University of Liverpool, MRC North West Hub for Trials Methodology Research<br>Blazeby, Jane; University of Bristol, Department of Social Medicine
**Primary Subject Heading**:	Research methods
Secondary Subject Heading:	Evidence based practice, Emergency medicine, Health services research
Keywords:	Clinical trials < THERAPEUTICS, STATISTICS & RESEARCH METHODS, ACCIDENT & EMERGENCY MEDICINE, QUALITATIVE RESEARCH

SCHOLARONE™
Manuscripts

A detailed systematic analysis of recruitment strategies in randomised controlled trials in patients with an unscheduled admission to hospital

C Rowlands[1], L Rooshenas[1], K Fairhurst[1], J Rees[2], C Gamble[3], JM Blazeby[1,2]

1. MRC ConDuCT-II Hub for Trials Methodology Research, School of Social & Community Medicine, University of Bristol, UK
2. Division of Surgery, Head & Neck, University Hospitals Bristol NHS Foundation Trust, Bristol, UK
3. MRC North West Hub for Trials Methodology Research, Institute of Translational Medicine, University of Liverpool, UK

Corresponding author: Dr Leila Rooshenas - Leila.Rooshenas@bristol.ac.uk

Word count = 3330 excluding tables, figures and references.

For peer review only

For peer review only - http://bmjopen.bmj.com/site/about/guidelines.xhtml

Abstract

Objectives To examine the design and findings of recruitment studies in RCTs involving patients with an unscheduled admission to hospital (UHA), to consider how to optimise recruitment in future RCTs of this nature.

Design Studies within the ORRCA database (Online Resource for Recruitment Research in Clinical Trials; www.orrca.org.uk) which reported on recruitment to RCTs involving UHAs in patients >18 years were included. Extracted data included trial clinical details, and the rationale and main findings of the recruitment study.

Studies were categorised according to the design of the recruitment study and the nature of the host trials (real or hypothetical). Data on the rationale for the recruitment study and the methods used to optimise recruitment were collected and summarised.

Results Of 3114 articles populating ORRCA, 39 recruitment studies were eligible, focusing on 68 real and 13 hypothetical host RCTs. Four studies were prospectively planned investigations of recruitment interventions, one of which was a nested RCT. Most recruitment papers were reports of recruitment experiences from one or more 'real' RCTs (n=24) or studies using hypothetical RCTs (n=11). Rationales for conducting recruitment studies included limited time for informed consent (IC) and patients being too unwell to provide IC. Methods to optimise recruitment included providing patients with trial information in the pre-hospital setting, technology to allow recruiters to cover multiple sites, screening logs to uncover recruitment barriers, and verbal rather than written information and consent.

Conclusion There is a paucity of high quality research into recruitment in RCTs involving UHAs with only one nested randomised study evaluating a recruitment intervention. Amongst the remaining studies methods to optimise recruitment focused on how to improve information provision in the pre-hospital setting and use of screening logs. Future research in this setting should focus on the prospective evaluation of the well-developed interventions to optimise recruitment.

Abstract word count = 297 words

For peer review only - http://bmjopen.bmj.com/site/about/guidelines.xhtml

Strengths and limitations of this study

- This review is the first to focus on the complex issue of recruitment to RCTs involving patients undergoing an unscheduled hospital admission
- This review is the first publication to utilise the ORRCA database in generating recruitment research
- The ORRCA database continues to evolve as updates encompass newly published recruitment research. Updates to the database may have generated further UHA research articles since the completion of this review.

For peer review only

For peer review only - http://bmjopen.bmj.com/site/about/guidelines.xhtml

Introduction

Recruitment to randomised controlled trials (RCTs) is the biggest obstacle to successful trial conduct.(1) Recruitment may be particularly challenging amongst patients who have an unscheduled hospital admission (UHA). In this situation, the condition of the potential participant and the demanding working environment for clinical teams can complicate the process of identification, screening, and informed consent. Patients are often in pain, unwell, and anxious about the underlying problem. There may also be time limitations due to the urgent need to deliver the clinical treatments under evaluation.(2, 3)

There are a number of existing systematic reviews of methods to optimise recruitment to trials in a variety of clinical contexts and patient populations, including cancer (4-6), primary care, (7) geriatrics (8, 9) and minority community patients (10), or a mix of clinical settings.(11-17) None specifically focus on recruitment of patients undergoing UHA.

Established methods exist for recruiting potential RCT participants who are unwell or unconscious and lack capacity. In these circumstances, permission for enrolment into an RCT may be sought from a surrogate decision maker (SDM) (18), or through deferred consent (also known as 'exception from informed consent'): a process whereby a participant is recruited into the trial in order for urgent treatment to be provided and subsequently asked to provide written consent for ongoing participation once they regain capacity.(19) However, recruitment may be particularly challenging where patients are acutely unwell, but retain capacity to decide on enrolment into an RCT. Reviewing the literature on how to optimise recruitment in this setting may lead to valuable insights, and identify areas where further research is needed.

The aim of this paper was to examine the design and findings of recruitment studies in RCTs involving patients with an unscheduled admission to hospital (UHA), to consider how to optimise recruitment in future RCTs of this nature..

Methods

Search strategy

Articles were identified through manually screening each entry within the ORRCA recruitment research database (Online Resource for Recruitment research in Clinical triAls;

For peer review only - http://bmjopen.bmj.com/site/about/guidelines.xhtml

http://www.orrca.org.uk/). The ORRCA project was funded by the UK Medical Research Council (MRC) Hubs for Trials Methodology Research Network (HTMRN). It provides a comprehensive online database of published empirical and non- empirical papers about recruitment to clinical research. ORRCA is populated from an extensive systematic search of the Cochrane Library, MEDLINE (Ovid), SCOPUS, ERIC and SCI-EXPANDED and SSCI (via ISI Web of Science). The search strategy employed by ORRCA was based on a Cochrane systematic review of trial recruitment.(16) Further details about the formation of the ORRCA database is reported on their website (http://www.orrca.org.uk/). In this review, a full up-to-date copy of the ORRCA database was obtained in January 2016, and the database was searched in February 2016. At this stage ORRCA contained publications relevant to recruitment published up to and including the end of December 2014. Publications from 2015 onwards were not available due to ongoing work in processing articles in the ORRCA database.

Study eligibility criteria

Any study held within ORRCA that reported on recruitment to RCTs involving UHAs was eligible for inclusion. UHA was defined as an unscheduled admission to hospital at short notice because of clinical need. This included pre-hospital care, intensive care (ICU) admissions, and accident and emergency (A&E) department attendances. Studies which reported on a mix of patients undergoing scheduled and unscheduled admissions were eligible if the findings for the UHA study population were described separately. Papers that reported recruitment strategies for a mixture of RCTs and other types of research (i.e. non-RCTs) were only eligible for inclusion if the recruitment strategies for RCTs were described separately. RCTs that involved children (age <18 years) or patients with acute psychiatric illness were excluded, as these patients would not normally be subject to the usual recruitment processes due to differences in the consent processes. Systematic reviews of methods to optimise recruitment were scrutinised for relevant primary articles, but not included in the analysis. Editorials, and studies of recruitment to non-RCTs were excluded. Abstracts were also excluded because these rarely included the necessary contextual information and data needed to make a meaningful contribution to the dataset for this study.

For peer review only - http://bmjopen.bmj.com/site/about/guidelines.xhtml

Screening and selection process

One author (CR) screened all articles included within the ORRCA database. Duplicate screening was carried out by one other author (KF) on 10% of the database. KF was blinded to the original screening decisions. Papers were assessed at title and abstract level according to the eligibility criteria. Differences in opinion were resolved by discussion between CR and KF, and any remaining differences in opinion were referred to another member of the study team (JMB) if required. The aim was to reach agreement for screening decisions on all studies within this sample. Agreement was reached on 271/300 articles screened. Of the 29 discrepancies raised, 22 were resolved following discussion between CR and KF. The seven remaining papers were discussed with a third author (JMB), which resulted in two of these papers being included and five being excluded. No paper which was suggested to be included by the second reviewer was eventually included in the review.

We calculated a kappa statistic for the double screened articles above. Ten per cent of articles were double screened with a Kappa = 0.677 (SE= 0.048, p <0.001) suggesting "good" agreement. As such, the remaining 90% of articles in the ORRCA database were screened by a single screener (CR). The third arbiter (JMB) involved in screening the 10% sample was also consulted for screening the remaining 90% of articles in the ORRCA database, in instances where the single screener (CR) was uncertain about an article's eligibility.

Definitions – host RCT

All studies focused on recruitment to one or more specific 'host' RCTs. In this paper, a host RCT refers to the underlying randomised controlled trial, (i.e. addressing a clinical question) in which the recruitment of participants took place. A host RCT could be a pilot or a main trial. Some recruitment papers reported on community consultations in which the views of the public were sought to establish the likelihood of recruitment success or acceptability of a proposed trial. This approach is typically used when the study population may be critically ill at the time of recruitment (and therefore may be unable to provide full, written informed consent).(20) In recognition of this, a clear differentiation was made between studies that focused on recruitment to an existing clinical RCT (a 'real' host RCT) versus potential recruitment to an RCT that did not exist (a 'hypothetical' host RCT), but is proposed to exist in order to estimate its acceptability to potential participants. A 'recruitment study' refers to research into the process of recruiting eligible participants, in the context of one or more real or hypothetical host RCTs.

For peer review only - http://bmjopen.bmj.com/site/about/guidelines.xhtml

Definitions - recruitment study design

In order to group similarly designed recruitment studies together and enhance data analysis a new categorisation system for different recruitment study designs was developed (Categories A to D). Consideration was given to the design of the recruitment study and whether a real or hypothetical host RCT was used. The categories are provided in Table 1.

Contacting study authors

If an appropriate recruitment study did not adequately describe the host trial, the study authors were contacted by email to determine whether the host trial met the eligibility criteria. Three attempts, each two weeks apart, were made to contact study authors. If no response was received, then the paper was excluded.

Data Extraction and Synthesis

CR extracted the data using a pre-designed and piloted data extraction form. Data extracted from eligible studies included clinical details of the underlying host RCT, the rationale for conducting a recruitment study, a summary of the recruitment study findings, recommendations for improving recruitment, and suggestions for further research. Where specific recruitment interventions had been evaluated, further details regarding the interventions were collected, including the timing of information exchange, informed consent, and randomisation. No statistical analyses were planned, as the review was expected to provide a descriptive analysis of results due to the anticipated heterogeneous nature of recruitment strategies presented. During the process, multiple meetings were undertaken with JB to examine papers and check data extraction processes as required.

Results

Study selection

A total of 3114 articles were identified within the ORRCA database. After initial screening at title and abstract level, 3044 articles were excluded, leaving 70 potentially eligible for which full texts were obtained. A further 31 articles were excluded following full text screening. Duplicate screening did not produce any discrepancies which could not be resolved through discussion. In total, 39 recruitment studies (21-58) were identified which reported results from 68 real host RCTs and 13 hypothetical host RCTs and were included in this review

For peer review only - http://bmjopen.bmj.com/site/about/guidelines.xhtml

Table 1: Number of included studies, host RCTs and hypothetical RCTs categorised according to the new recruitment study types (A to D)

Category	Recruitment study design	Recruitment studies (n=39)	Real host RCTs (n=68)	Hypothetical host RCTs (n=13)
A	Randomised controlled trials (RCTs) of interventions to optimise recruitment nested within one or more host RCTs	1	1	N/A

For peer review only - http://bmjopen.bmj.com/site/about/guidelines.xhtml

**B**	Prospectively designed, non-randomised studies of interventions to optimise recruitment to one or more host RCTs	3	3	N/A
**C**	Studies describing recruitment experiences involving one or more host RCTs	24	64*	N/A
**D**	Studies to consider recruitment within proposed hypothetical RCTs (commonly known as community consultations)	11	N/A	13**

RCT = randomised controlled trial, N/A = not applicable – category does not apply to type of host RCT

*The 24 recruitment studies reported data from 64 real host RCTs i.e. a number of recruitment studies reported data from more than one real host RCT

**The 11 recruitment studies reported data from 13 hypothetical studies i.e. two recruitment studies reported data from more than one hypothetical host RCT

For peer review only - http://bmjopen.bmj.com/site/about/guidelines.xhtml

(Figure 1). A number of recruitment studies described results obtained from more than one real or hypothetical RCT.

The 68 real host trials (around which recruitment was focused) were predominantly multi-centre RCTs (63/68) with large study populations (median = 624 participants, range = 4-58,050), and typically evaluated non-invasive medical interventions (61/68) (Table 2). The apparent predominance of RCTs in neurology is caused by one recruitment study which included data from 32 separate RCTs. With exception to this, the clinical settings of the host RCTs varied, encompassing several medical disciplines.

The majority of recruitment studies were simple descriptive non-randomised studies reporting previous experiences/challenges of recruitment to a host RCT (Category C, n =24). There were 11 that proposed a hypothetical RCT to a study population (Category D, n =11). Only one article used what is considered to be the optimal method for evaluating an intervention - a randomised comparison of a recruitment intervention nested within a host RCT (Category A). Three studies prospectively evaluated recruitment interventions using non-randomised study designs (Category B) (Table 1).

RCTs of interventions to optimise recruitment nested within one or more host RCTs (Category A)

Only one of the included recruitment studies investigated two recruitment strategies using a randomised design (Category A studies, Table 3). The rationale for this study was the limited time available for recruitment due the acute medical treatment required by patients. (21) Patients randomised to the intervention group received 'advanced notification' of the trial (via fax or phone) designed to offer patients more time to consider trial participation, compared to the control group who only received information once they met with the clinical team. Consent to participate in the host RCT was obtained in 27/50 (54%) and 25/50 (50%) patients in the intervention and control groups respectively (P= 0.69). Although no improvement in overall recruitment rates was demonstrated using advance notification, the provision of early information was demonstrated to be feasible.

For peer review only - http://bmjopen.bmj.com/site/about/guidelines.xhtml

Table 2: Summary characteristics of the host RCTs and hypothetical host RCTs in this review

Trial characteristic	Real RCTs (n = 68)	Hypothetical RCTs (n = 13)	Total (n=81)
**Clinical Setting**			
Neurology	39*	4	43
Cardiology	8	4	12
Obstetrics	3	1	4
Infection	3	0	3
Trauma	6	3	9
Critical care	9	1	10
**Type of interventions**			
Invasive / non-invasive	5 / 61	4 / 9	9 / 70
Unknown	2	0	2
**Trial design**			
Main RCT/ pilot RCT	65/3	n/a	65 / 3
Two groups / more than two groups	63 / 5	11 / 2	74 / 7
**Number of centres****			
Single centre / multi-centre / unknown	3 / 63 / 2	0 / 4 / 9	3 / 67 / 11
<20 centres	18		
20 -100 centres	33		
>100 centres	15		
Median number of centres (range)	45 (1 - 818)		
**Number of participants****			
<500 participants	22		
500 -1500 participants	32		
>1500 participants	14		
Median number of participants (range)	624 (4 - 58,050)		

*One recruitment paper included data from 32 Stroke host RCTs. RCT = randomised controlled trial

** Hypothetical RCT data did not include information beyond single or multicentre, nor the suggested no. of participants

For peer review only - http://bmjopen.bmj.com/site/about/guidelines.xhtml

Prospectively designed, non-randomised studies of interventions to optimise recruitment to one or more host RCTs (Category B)

The common rationale for this type of recruitment study design (Category B studies, Table 3) was the limitations of time when dealing with patients who required acute treatment. The need for urgent treatment was thought to hinder the ability of the study team to gain informed consent (IC) for trial participation.

The strategy of optimising information provision in the pre-hospital setting used in the one Category A study was mirrored by two Category B studies, which utilised the presence of pre-hospital staff to engage potential trial participants.(22, 24) Recruitment in these studies was reportedly optimised through provision of brief verbal information to participants as they travelled to hospital, and initial verbal consent that sought permission to deliver the emergency trial intervention. Further information was provided and written consent was subsequently obtained when the patients were stabilised, in hospital. One of these studies (24) also provided training to pre-hospital staff to improve their understanding of trial conduct, and devised a simple assessment of capacity to ensure that patients' initial verbal consent was valid.

The remaining prospectively designed (Category B) study focused on using technology to enhance recruitment during an influenza outbreak.(23) As patient numbers would be expected to rise rapidly across a wide geographical area, the study team devised a system which provided them with automated, real-time alerts whenever an eligible participant was identified in each hospital. This allowed one centralised study team to cover numerous study sites, enhancing recruitment opportunities. Although none of these studies provided numerical evidence of the effectiveness of their recruitment strategies, all authors concluded that their presented strategies were feasible and acceptable for use in UHA RCTs.

Studies describing recruitment experiences involving one or more host RCTs (Category C)

Rationales for reporting authors' experiences of recruitment were similar to those in prospectively designed studies, including the limited time available for consent (n=13), and recruitment difficulties caused by the clinical condition of the patients (n=18) (Category C studies, Table 4). Some studies were prompted by a host trial encountering recruitment difficulties (n=3). Of the 24 non-randomised studies describing recruitment experiences involving one or more host RCTs, 14 were observational and five were qualitative studies.

For peer review only - http://bmjopen.bmj.com/site/about/guidelines.xhtml

Table 3: Rationale and types of recruitment interventions reported in Category A or B studies (i.e. prospectively nested within RCTs)

Author and year	Rationale(s)	Descriptive summary of recruitment intervention(s)	Classification of recruitment intervention	Timing of information provision regarding host RCT	Timing of informed consent for host RCT	Timing of randomisation for host RCT
**Prospectively designed RCTs of interventions to optimise recruitment nested within one or more host RCTs (Category A studies)**						
Leira** 2009	Limited time for IC in host RCT due to the clinical condition requiring urgent treatment.	Intervention group: 'Advance notification' about host RCT using phone and fax prior to meeting pre-hospital team<br><br>Control group: No information prior to meeting pre-hospital team	Advance notification using phone and fax	Intervention group: 'Advance notification' prior to face-to face meeting with pre-hospital team<br><br>Control group: During face-to-face meeting with pre-hospital team	Intervention group: During face-to-face meeting with pre-hospital team<br><br>Control group: During face-to-face meeting with pre-hospital team	Intervention group: Whilst patient in pre-hospital setting.<br><br>Control group: Whilst patient in pre-hospital setting.
**Prospectively designed, non-randomised studies of interventions to optimise recruitment to one or more host RCTs (Category B studies)**						
Chow 2011	Recruitment team would be overwhelmed by covering multiple sites over a wide geographical area.	An automated service notified recruitment team in real time when a potentially eligible participant was identified across multiple centres	Automated service to facilitate real time notifications to recruitment team covering multiple sites	During face-to-face meeting with recruitment staff (once eligibility confirmed)	During face-to-face meeting with recruitment team	Whilst patient in hospital.
Shaw 2014	Limited time for IC in host RCT due to the clinical condition requiring urgent treatment.<br><br>Pre-hospital staff have limited experience of RCTs	Standardised verbal information was provided by pre-hospital staff before a simple capacity assessment, verbal consent and delivery of emergency treatment<br><br>Host RCT procedures were tailored towards pre-hospital staff, who also attended a one-day training event	Optimising information provision in pre-hospital setting.<br><br>Allowing verbal consent from patient or SDM.<br><br>Training of pre-hospital staff	Simple verbal information provided in pre-hospital setting.<br><br>Further full information provided in hospital	Initial verbal consent given in pre-hospital setting.<br><br>Written informed consent gained in hospital.	Whilst patient in pre-hospital setting.
Beshansky 2014	Limited time for IC in host RCT due to the clinical condition requiring urgent treatment.	Standardised verbal information was provided by pre-hospital staff before a simple capacity assessment, verbal consent and delivery of emergency treatment	Optimising information provision in pre-hospital setting.<br><br>Allowing verbal consent from patient.	Simple verbal information provided in pre-hospital setting.<br><br>Further full information provided in hospital	Initial verbal consent in pre-hospital setting.<br><br>Written informed consent in hospital.	Whilst patient in pre-hospital setting.

RCT = randomised controlled trial, IC= informed consent, SDM = surrogate decision maker

** Leira et al – patients were randomised to the *recruitment* intervention or control group prior to the recruitment team seeing the patient, and without consent from the participant.

For peer review only - http://bmjopen.bmj.com/site/about/guidelines.xhtml

Table 4: Frequency of rationales, study designs and recommendations from non-randomised studies describing recruitment experiences involving one or more host RCTs (i.e. Category C studies).

Recruitment study characteristic	Description	Frequency in Category C recruitment studies (n=24)**
**Rationale**	Patients too unwell to provide IC	18
	Limited time for IC in host RCT due to the clinical condition requiring urgent treatment	13
	Host RCT not meeting recruitment targets (at one or more sites) or terminated due to poor recruitment	3
	To better understand the impact of altering eligibility criteria on recruitment	2
	To better understand the impact of availability of SDMs on recruitment	2
	To better understand the recruitment process in a host RCT	1
	To better understand clinicians reasons for refusing patient participation in host RCT	1
**Recruitment study design**	Observational study of recruitment	14
	Qualitative studies of host participants/SDMs or PIS	5
	Survey of host RCT participants	2
	Survey of clinical staff involved in host RCT	1
	Simulation study evaluating the effect of altering eligibility criteria in multiple host RCTs	1
	Meta-analysis of recruitment data in host RCTs	1
**Recommendations for optimising recruitment in future RCTs or areas for further research into recruitment*** **	To provide RCT information verbally and allow a verbal consent process	10
	To use a screening log can to provide insight into recruitment difficulties	8
	Patients or SDM were unable to recall key RCT information after providing IC***	5
	To use a 'waiver of consent'/ 'deferred consent'/ 'EFIC'	4
	To perform regular site visits	3
	To use a broad eligibility criteria /broad therapeutic window	2
	To use SDMs	2
	Novel methods for obtaining IC are required***	2
	To replace poorly recruiting centres	1
	To approach more eligible patients	1
	To survey staff involved with host RCT to provide insight into recruitment difficulties	1

RCT = randomised controlled trial, IC= informed consent, EFIC = exception from informed consent, SDM = surrogate decision maker, PIS= patient information sheet.
**each study may appear more than once in the relevant characteristics section (e.g. if it described more >1 rationale, or produced >1 finding/recommendation)
*** items for further research and not recommendations for optimising recruitment

For peer review only - http://bmjopen.bmj.com/site/about/guidelines.xhtml

These studies reported experiences of trial participants and surrogate decision makers (SDMs), or extracted verbatim information from written patient information sheets (PISs). Recommendations for optimising recruitment also mirrored Category A and B studies, highlighting the acceptability of verbal information provision and consent, or deferring consent altogether until an unwell patient is suitably stabilised. Additional benefits were seen in RCTs which used data from screening logs to uncover recruitment barriers, and trials which performed regular site visits.(29-31, 33, 35, 38, 39, 44) A sub section of qualitative studies, although not presenting recommendations for future trial conduct, highlighted their findings that many patients or SDMs who had provided consent to participate in an RCT did not recall much of the information provided to them during the consent process, suggesting that work was needed to improve consent in this setting.(31, 32, 40)

Studies to consider recruitment within proposed hypothetical RCTs (commonly known as community consultations) (Category D)

Eleven studies reported community views about proposed 'hypothetical' RCTs (Category D studies, Table 5). Although the rationales for conducting the studies were similar to studies involving real host RCTs (unwell patients and lack of time for consent), the study designs were varied including questionnaire surveys, interviews, and focus group meetings. Verbal information provision, verbal consent, and recruitment in the pre-hospital setting were identified as helpful recruitment strategies in these studies. However, these articles raised new issues around the appropriateness of using of SDMs when patients are too unwell to provide consent for themselves, and raised additional issues around who the SDM should be (next of kin (NOK), or an available physician). Some studies found a preference for the use of SDMs,(50, 52, 56) while others expressed that NOK or physicians should not be used for their specific hypothetical RCTs.(53, 55, 58)

Discussion

This review aimed to examine and summarise studies and methods used to optimise recruitment in RCTs in patients with an unscheduled hospital admission (UHA). It had the purpose of using the information to consider how to optimise recruitment in this challenging clinical setting in future studies. In the ORCCA database of recruitment research, only 39 out of a possible 3114 articles (1.25%) focused on recruitment to RCTs in the UHA setting. Only one of these studies was a randomised comparison of recruitment strategies; the majority of

For peer review only - http://bmjopen.bmj.com/site/about/guidelines.xhtml

Table 5: Frequency of rationales, study designs, main findings and recommendations from Non-randomised studies designed study to consider recruitment within proposed hypothetical RCTs (i.e. Category D studies, commonly known as community consultations).

Recruitment study characteristic	Description	Frequency in Category D recruitment studies (n=11)**
**Rationale**	Patients too unwell to provide IC	9
	Limited time for IC in host RCT due to the clinical condition requiring urgent treatment	6
	To explore the accuracy of decisions made by NOK when acting as SDM	1
**Recruitment study design**	Questionnaire survey	5
	Face-to-face interview	4
	Telephone survey	1
	Focus group meetings	1
**Recommendations for optimising recruitment in future RCTs or main findings*** **	To use a physician as a SDM	4
	To use NOK as a SDM	4
	To use EFIC	3
	**Not** to use NOK as a SDM	2
	To provide RCT information verbally and allow a verbal consent process	1
	To allow recruitment in pre-hospital setting	1
	To perform community consultations to estimate host RCT recruitment rates***	1
	**Not** to use a physician as a SDM	1
	**Not** to use EFIC	1
	To perform community consultations to aid selection of relevant study outcomes***	1

*RCT = randomised controlled trial, EFIC = exception from informed consent, NOK = next of kin, SDM = surrogate decision maker
**each study may appear more than once in the relevant characteristics section (e.g. if it described more >1 rationale, or produced >1 finding/recommendation)
*** items reported as main findings, but not recommendations for optimising recruitment

For peer review only - http://bmjopen.bmj.com/site/about/guidelines.xhtml

studies consisted of simple study designs describing recruitment experiences. Eleven further studies involved hypothetical RCTs, and whilst of some value, it is uncertain how these types of investigations translate into optimal RCT design. This work therefore highlights the need for development of interventions to optimise recruitment in the UHA setting and prospective evaluation of their effectiveness and acceptability.

Comparison to existing literature

There have been several reviews that have summarised the evidence for optimising recruitment in trials in other clinical conditions or contexts, including cancer (4-6), geriatrics (8, 9), primary care (7), and a mix of clinical settings.(11-17). Similar to our findings, these reviews have commonly highlighted the lack of high quality evaluations of recruitment interventions (6,16). Despite this, some of the reviews have identified effective recruitment strategies, although these vary in the extent to which they are likely to be transferrable to the UHA setting. For example, it is unclear if interventions such as telephone reminders (16,17), 'education sessions' about the health condition (11), and use of monetary incentives (11,17) are as effective or appropriate in the UHA context, given the specific factors that may compromise recruitment in this setting (e.g. patients in pain/distress, short timeframes for recruitment, busy settings, etc.). Other reviews have drawn attention to interventions aimed at recruiters, such as appropriate training/guidance (12), reduction of clinical workload (7,12), and 'research protected' time (12). These have potential to be helpful in trials conducted in the UHA setting, although further research is needed to examine the content/nature of the training materials needed, and the logistics of implementing these types of interventions. Finally, some reviews have shown that features of RCT study design – such as open, rather than placebo-controlled trials - are associated with better recruitment outcomes (16,17). Recommendations that advise against particular study designs may limit the quality of evidence generated to guide future patient care, and dissuade from the most important (and appropriate) clinical questions from being addressed. Furthermore, there is a growing body of evidence to indicate that it is possible to recruit to more complex RCTs with appropriate training and support.(59) More generally, use of integrated qualitative research to understand and address recruitment difficulties is being increasingly recommended in more recent reviews (12)(60), and is likely to inform novel insights if applied to trials in UHA settings.

For peer review only - http://bmjopen.bmj.com/site/about/guidelines.xhtml

Some reviews and individual studies have produced recommendations that are likely to be particularly relevant for developing UHA-specific recruitment strategies for future evaluation. These strategies may tackle some of the context-specific difficulties that are likely to be experienced in UHA settings. For example, one systematic review focusing on recruitment to RCTs involving patients with cancer or organ failure highlighted the potential for providing audio-visual information (such as a video to explain the RCT) to facilitate RCT recruitment (59). Such an approach may be helpful in the UHA setting, given that that large amounts of written information may not be appropriate in patients who are in pain or feeling distressed. This recommendation is also in keeping with guidance issued by the NHS Health Research Authority (HRA), which suggests using alternatives to written information (61).

A verbal exchange of information and providing initial verbal consent has been previously suggested as a preferred strategy to written alternatives in RCT taking place in the emergency setting (62). It is agreed that for UHAs this could be preferable, although it is considered that further work is needed to develop this type of verbal consent to ensure that quality assurance is still achieved for consent. Another suggested solution to this problem is the use of an independent patient advocate, who may oversee such conversations between trial team members and acutely unwell patients, to verify that appropriate information exchange took place, and to act as an assessor of a patient's willingness to participate in the RCT.(63) This strategy provides one potential solution to a significant obstacle in recruitment of patients undergoing an UHA. However, it may be practically difficult to achieve given that UHAs can occur at any time of the day or night and such trained patient advocates would also need to be available during these times making a trial more expensive.

Finally, a non-systematic review article focusing on recruitment to emergency medicine research also highlighted a similar problem in a lack of high quality evidence on recruitment. It suggested support for the use of deferred consent (also known as exception from informed consent) which was highlighted in this review and additionally raised the issue that the use of SDMs for consent may be problematic due to the pressures of time and the emotional stress family members will be under while a relative is acutely unwell.(3)

Strengths and weaknesses of this study

This review is the first to systematically focus on recruitment strategies in the UHA setting. With a reported growing number of unscheduled hospital admissions presenting great

For peer review only - http://bmjopen.bmj.com/site/about/guidelines.xhtml

challenges to modern-day health care provision, the conduct of RCTs in the UHA will inevitably develop as an area of research.

The review may be limited through its reliance on a single search of the ORRCA database, conducted in February 2016, at which point the database contained publications relevant to recruitment published up to the end of December 2014. The ORRCA database continues to evolve as updates encompass newly published recruitment research. Updates to the database may have generated further UHA research articles since the search for this review. It is possible that any new update could make an important and significant contribution to this field because so little has been done in this area thus far.

The review may be limited because a single researcher reviewed the majority of the ORRCA entries, and it is possible that ORRCA may not have included all relevant articles in the first place. This work may also be at risk of publication bias, as we chose to exclude abstracts based on the assumption that these were unlikely to include all the data items we were interested in. Excluding abstracts may have resulted in omission of some potentially valuable information. Although some authors recommend the inclusion of conference abstracts within a review (64, 65), there is some evidence to suggest that there is discordance between the content of abstracts and the subsequent full-text publication, and as such, including abstracts may introduce unreliable data. (66, 67) Another weakness is that the majority of recruitment studies retrospective analyses of processes and events that occurred during the host RCT. These data were not necessarily collected with the intention of evaluating RCT recruitment strategies. This may limit the quality of the data and the utility of the recommendations arising from the included studies.

Finally, a risk of bias assessment of the included recruitment studies was not performed, because only one of the recruitment studies was an RCT (i.e. a randomised, controlled evaluation of a recruitment intervention).

Unanswered questions and future research

Some articles within this review demonstrated inconsistent conclusions about the value of surrogate decision makers, who these should be, and how these should operate. Future research should examine these issues in more depth, in a variety of clinical contexts,

For peer review only - http://bmjopen.bmj.com/site/about/guidelines.xhtml

focusing on the roles of SDM in different RCTs and to what extent the public it acceptable for an SDM to decide on their behalf for trials involving acutely unwell patients.

It is uncertain how well the results of studies using hypothetical RCTs can be translated to the conduct of real RCTs. There may be some validity in the findings from hypothetical RCTs, as highlighted in this review by the similarity of the results from studies which used real RCTs and hypothetical RCTs. Further work is required to ascertain the extent to which findings from studies which use hypothetical RCTs reliably influence the design and conduct of real RCTs.

As part of this review a classification system was devised in order to group together similarly designed recruitment research studies. Before any such classification system could be used more widely, it would require validation by testing its applicability to at least one further set of recruitment papers, preferably in a different health care setting.

This review highlighted strategies to deal with patients in the pre-hospital setting who required urgent treatment. However, not all patients who are cared for in the pre-hospital setting require treatment immediately. Some may require transport to hospital for further assessment and potential treatment. Further research could explore whether providing early trial information to such patients based on their symptoms or presumed diagnosis, could affect trial recruitment, should treatment be required later. This strategy could prove to be useful in a broader range of unscheduled hospital admissions.

Although some recommendations for optimising recruitment could be drawn from this review, the overall lack of research in this area, particularly amongst high quality, methodologically robust studies, is a limiting factor. Future recruitment studies in this clinical setting should focus on studies with higher methodological rigour, by developing novel interventions to optimise recruitment and prospectively evaluating their effectiveness through an appropriate study design.

Conclusion

There is a relative paucity of high quality research on strategies to optimise recruitment to RCTs involving unscheduled hospital admissions. Some emerging recommendations include

For peer review only - http://bmjopen.bmj.com/site/about/guidelines.xhtml

optimising information provision about the trial in the pre-hospital setting to improve recruitment where treatment is required urgently, or using technology to facilitate recruitment across many hospital sites. Screening log data can also provide useful insight to specific barriers to recruitment. Future research in this setting should focus on conducting studies with higher methodological rigour, by developing interventions to optimise recruitment and prospectively evaluating their effectiveness.

Acknowledgements

The following are gratefully acknowledged for their assistance and support with this work: Shaun Treweek, Anna Kearney, Nicola Harman.

Funding

CR is supported by the Medical Research Council (MRC) Hub for Trials Methodology Research Network and the Collaboration and Innovation for Difficult Trials in Invasive procedures (ConDuCT-II) Hub for Trials Methodology Research (MR/K025643/1). The funders had no role in study design, data collection and analysis, decision to publish, or preparation of manuscript.

Contributor statement

CR, LR JR, and JMB designed the study and formulated the protocol. CR and KF performed all data extraction, which was reviewed and critically analysed by CR, LR and JMB. CG facilitated access to the ORRCA database and critically appraised the review. CR produced the first draft of the manuscript and LR and JMB made substantial contributions to the formulation of the final version. All authors read and approved the final manuscript.

Competing interests

No competing interests

Data sharing statement

As this is a review of published literature, there are no additional data available.

Figure 1: Study selection PRISMA flow diagram

References

For peer review only

1. Tudur Smith C, Hickey H, Clarke M, Blazeby J, Williamson P. The trials methodological research agenda: results from a priority setting exercise. Trials. 2014;15:32-.
2. Schandelmaier S, von Elm E, You JJ, Blumle A, Tomonaga Y, Lamontagne F, et al. Premature Discontinuation of Randomized Trials in Critical and Emergency Care: A Retrospective Cohort Study. Critical care medicine. 2016;44(1):130-7.
3. Cofield SS, Conwit R, Barsan W, Quinn J. Recruitment and retention of patients into emergency medicine clinical trials. Academic emergency medicine : official journal of the Society for Academic Emergency Medicine. 2010;17(10):1104-12.
4. Boland J, Currow DC, Wilcock A, Tieman J, Hussain JA, Pitsillides C, et al. A systematic review of strategies used to increase recruitment of people with cancer or organ failure into clinical trials: implications for palliative care research. Journal of pain and symptom management. 2015;49(4):762-72.e5.
5. Fayter D, McDaid C, Eastwood A. A systematic review highlights threats to validity in studies of barriers to cancer trial participation. Journal of clinical epidemiology. 2007;60(10):990-1001.
6. Mc Daid C, Hodges Z, Fayter D, Stirk L, Eastwood A. Increasing participation of cancer patients in randomised controlled trials: a systematic review. Trials. 2006;7:16.
7. Ngune I, Jiwa M, Dadich A, Lotriet J, Sriram D. Effective recruitment strategies in primary care research: a systematic review. Quality in primary care. 2012;20(2):115-23.
8. Auster J, Janda M. Recruiting older adults to health research studies: A systematic review. Australasian journal on ageing. 2009;28(3):149-51.
9. Provencher V, Mortenson WB, Tanguay-Garneau L, Belanger K, Dagenais M. Challenges and strategies pertaining to recruitment and retention of frail elderly in research studies: a systematic review. Archives of gerontology and geriatrics. 2014;59(1):18-24.
10. Ibrahim S, Sidani S. Strategies to recruit minority persons: a systematic review. Journal of immigrant and minority health / Center for Minority Public Health. 2014;16(5):882-8.
11. Caldwell PH, Hamilton S, Tan A, Craig JC. Strategies for increasing recruitment to randomised controlled trials: systematic review. PLoS medicine. 2010;7(11):e1000368.
12. Fletcher B, Gheorghe A, Moore D, Wilson S, Damery S. Improving the recruitment activity of clinicians in randomised controlled trials: a systematic review. BMJ open. 2012;2(1):e000496.
13. Huynh L, Johns B, Liu SH, Vedula SS, Li T, Puhan MA. Cost-effectiveness of health research study participant recruitment strategies: a systematic review. Clinical trials (London, England). 2014;11(5):576-83.
14. Mapstone J, Elbourne D, Roberts I. Strategies to improve recruitment to research studies. The Cochrane database of systematic reviews. 2007(2):Mr000013.
15. Raftery J, Bryant J, Powell J, Kerr C, Hawker S. Payment to healthcare professionals for patient recruitment to trials: systematic review and qualitative study. Health technology assessment (Winchester, England). 2008;12(10):1-128, iii.

For peer review only - http://bmjopen.bmj.com/site/about/guidelines.xhtml

16. Treweek S, Mitchell E, Pitkethly M, Cook J, Kjeldstrom M, Taskila T, et al. Strategies to improve recruitment to randomised controlled trials. The Cochrane database of systematic reviews. 2010(1):Mr000013.
17. Watson JM, Torgerson DJ. Increasing recruitment to randomised trials: a review of randomised controlled trials. BMC medical research methodology. 2006;6:34.
18. Burns KE, Prats CJ, Maione M, Lanceta M, Zubrinich C, Jeffs L, et al. The Experience of Surrogate Decision Makers on Being Approached for Consent for Patient Participation in Research. A Multicenter Study. Annals of the American Thoracic Society. 2017;14(2):238-45.
19. Whitesides LW, Baren JM, Biros MH, Fleischman RJ, Govindarajan PR, Jones EB, et al. Impact of individual clinical outcomes on trial participants' perspectives on enrollment in emergency research without consent. Clinical trials (London, England). 2017;14(2):180-6.
20. Fordyce CB, Roe MT, Dickert NW. Maximizing value and minimizing barriers: Patient-centered community consultation for research in emergency settings. Clinical trials (London, England). 2017;14(1):88-93.
21. Leira EC, Ahmed A, Lamb DL, Olalde HM, Callison CR, Torner JC, et al. Extending Acute Trials to Remote Populations A Pilot Study During Interhospital Helicopter Transfer. Stroke. 2009;40(3):895-901.
22. Beshansky JR, Sheehan PR, Klima KJ, Hadar N, Vickery EM, Selker HP. A community consultation survey to evaluate support for and success of the IMMEDIATE trial. Clinical Trials. 2014;11(2):178-86.
23. Chow E, Zuberi M, Seto R, Hota S, Fish EN, Morra D. Using real-time alerts for clinical trials Identifying potential study subjects. Applied Clinical Informatics. 2011;2(4):472-80.
24. Shaw L, Price C, McLure S, Howel D, McColl E, Younger P, et al. Paramedic Initiated Lisinopril For Acute Stroke Treatment (PIL-FAST): results from the pilot randomised controlled trial. Emerg Med J. 2014;31(12):994-9.
25. Abramson NS, Safar P. Deferred consent: use in clinical resuscitation research. Brain Resuscitation Clinical Trial II Study Group. Annals of Emergency Medicine. 1990;19(7):781-4.
26. Adeoye O, Pancioli A, Khoury J, Moomaw CJ, Schmit P, Ewing I, et al. Efficiency of Enrollment in a Successful Phase II Acute Stroke Clinical Trial. Journal of Stroke & Cerebrovascular Diseases. 2012;21(8):667-72.
27. Ågård A, Hermerén G, Herlitz J. Patients' experiences of intervention trials on the treatment of myocardial infarction: Is it time to adjust the informed consent procedure to the patient's capacity? Heart. 2001;86(6):632-7.
28. Annane D, Outin H, Fisch C, Bellissant E. The effect of waiving consent on enrollment in a sepsis trial. Intensive Care Medicine. 2004;30(2):321-4.
29. Bellomo R, Trial RRT. Screening and Study Enrolment in the Randomized Evaluation of Normal vs. Augmented Level (RENAL) Replacement Therapy Trial. Blood Purification. 2009;27(2):199-205.
30. Burns KEA, Zubrinich C, Tan WL, Raptis S, Xiong W, Smith O, et al. Research Recruitment Practices and Critically Ill Patients A Multicenter, Cross-Sectional Study (The Consent Study). American Journal of Respiratory and Critical Care Medicine. 2013;187(11):1212-8.

For peer review only

For peer review only - http://bmjopen.bmj.com/site/about/guidelines.xhtml

31. Chlan L, Guttormson J, Tracy MF, Bremer KL. STRATEGIES FOR OVERCOMING SITE AND RECRUITMENT CHALLENGES IN RESEARCH STUDIES BASED IN INTENSIVE CARE UNITS. American Journal of Critical Care. 2009;18(5):410-7.
32. Collins JF. Data and safety monitoring board issues raised in the VA Status Epilepticus Study. Controlled Clinical Trials. 2003;24(1):71-7.
33. Cook D, Arabi Y, Ferguson ND, Heels-Ansdell D, Freitag A, McDonald E, et al. Physicians declining patient enrollment in a critical care trial: a case study in thromboprophylaxis. Intensive Care Medicine. 2013;39(12):2115-25.
34. Costescu DJW, Cullimore AJ. Lessons learned from a resident-led clinical trial in obstetrics. Clinical Trials. 2013;10(4):612-6.
35. Crowley ST, Chertow GM, Vitale J, O'Connor T, Zhang JN, Schein RMH, et al. Lessons for successful study enrollment from the Veterans Affairs/National Institutes of Health Acute Renal Failure Trial Network Study. Clinical Journal of the American Society of Nephrology. 2008;3(4):955-61.
36. Elkins JS, Khatabi T, Fung L, Rootenberg J, Johnston SC. Recruiting subjects for acute stroke trials: a meta-analysis. Stroke [Internet]. 2006; 37(1):[123-8 pp.]. Available from: http://onlinelibrary.wiley.com/o/cochrane/clcmr/articles/CMR-10639/frame.html
http://stroke.ahajournals.org/content/37/1/123.full.pdf.
37. Flaherty ML, Karlawish J, Khoury JC, Kleindorfer D, Woo D, Broderick JP. How important is surrogate consent for stroke research? Neurology. 2008;71(20):1566-71.
38. Foster D, Cook D, Granton J, Steinberg M, Marshall J. Use of a screen log to audit patient recruitment into multiple randomized trials in the intensive care unit. Canadian Critical Care Trials Group. Critical care medicine [Internet]. 2000; 28(3):[867-71 pp.]. Available from:
http://onlinelibrary.wiley.com/o/cochrane/clcmr/articles/CMR-11625/frame.html.
39. Glassberg AE, Luce JM, Matthay MA. Reasons for nonenrollment in a clinical trial of acute lung injury. Chest [Internet]. 2008; 134(4):[719-23 pp.]. Available from:
http://onlinelibrary.wiley.com/o/cochrane/clcmr/articles/CMR-12450/frame.html
http://journal.publications.chestnet.org/data/Journals/CHEST/22076/zcb01008000719.pdf.
40. Kendall B, Stadeli R, Schegg B, Olbrich M, Chen E, Harmelin-Kadouri R, et al. Clinical Trial Educator program - a novel approach to accelerate enrollment in a phase III International Acute Coronary Syndrome Trial. Clinical Trials. 2012;9(3):358-66.
41. Kenyon S, Dixon-Woods M, Jackson CJ, Windridge K, Pitchforth E. Participating in a trial in a critical situation: a qualitative study in pregnancy. Quality & Safety in Health Care. 2006;15(2):98-101.
42. Roozenbeek B, Maas AIR, Marmarou A, Butcher I, Lingsma HF, Lu J, et al. The influence of enrollment criteria on recruitment and outcome distribution in traumatic brain injury studies: results from the impact study. Journal of Neurotrauma [Internet]. 2009; 26(7):[1069-75 pp.]. Available from:
http://onlinelibrary.wiley.com/o/cochrane/clcmr/articles/CMR-14708/frame.html
http://online.liebertpub.com/doi/pdfplus/10.1089/neu.2008.0569.
43. Schats R, Brilstra EH, Rinkel GJ, Algra A, Gijn J. Informed consent in trials for neurological emergencies: the example of subarachnoid haemorrhage. Journal of Neurology, Neurosurgery, and Psychiatry [Internet]. 2003; 74(7):[988-91 pp.].

For peer review only - http://bmjopen.bmj.com/site/about/guidelines.xhtml

Available from: http://onlinelibrary.wiley.com/o/cochrane/clcmr/articles/CMR-12831/frame.html
http://www.ncbi.nlm.nih.gov/pmc/articles/PMC1738516/pdf/v074p00988.pdf.
44. Smith OM, McDonald E, Zytaruk N, Foster D, Matte A, Clarke F, et al. Rates and determinants of informed consent: A case study of an international thromboprophylaxis trial. Journal of Critical Care. 2013;28(1):28-39.
45. Smyth RM, Jacoby A, Elbourne D. Deciding to join a perinatal randomised controlled trial: experiences and views of pregnant women enroled in the Magpie Trial. Midwifery. 2012;28(4):E478-85.
46. Williams BF, French JK, White HD. Informed consent during the clinical emergency of acute myocardial infarction (HERO-2 consent substudy): A prospective observational study. Lancet. 2003;361(9361):918-22.
47. Yamal JM, Robertson CS, Rubin ML, Benoit JS, Hannay HJ, Tilley BC. Enrollment of racially/ethnically diverse participants in traumatic brain injury trials: Effect of availability of exception from informed consent. Clinical Trials. 2014;11(2):187-94.
48. Yuval R, Halon DA, Merdler A, Khader N, Karkabi B, Uziel K, et al. Patient comprehension and reaction to participating in a double-blind randomized clinical trial (ISIS-4) in acute myocardial infarction. Archives of Internal Medicine. 2000;160(8):1142-6.
49. Abboud PA, Heard K, Al-Marshad AA, Lowenstein SR. What determines whether patients are willing to participate in resuscitation studies requiring exception from informed consent? Journal of Medical Ethics [Internet]. 2006; 32(8):[468-72 pp.]. Available from: http://onlinelibrary.wiley.com/o/cochrane/clcmr/articles/CMR-10612/frame.html.
50. Ali K, Roffe C, Crome P. What patients want: consumer involvement in the design of a randomized controlled trial of routine oxygen supplementation after acute stroke. Stroke [Internet]. 2006; 37(3):[865-71 pp.]. Available from: http://onlinelibrary.wiley.com/o/cochrane/clcmr/articles/CMR-10638/frame.html
http://stroke.ahajournals.org/content/37/3/865.full.pdf.
51. Bulger EM, Schmidt TA, Cook AJ, Brasel KJ, Griffiths DE, Kudenchuk PJ, et al. The Random Dialing Survey as a tool for community consultation for research involving the emergency medicine exception from informed consent. Annals of Emergency Medicine [Internet]. 2009; 53(3):[341-50 pp.]. Available from: http://onlinelibrary.wiley.com/o/cochrane/clcmr/articles/CMR-14468/frame.html
http://ac.els-cdn.com/S0196064408015126/1-s2.0-S0196064408015126-main.pdf?_tid=3127512a-62ab-11e5-948a-00000aab0f27&acdnat=1443092490_2b79f56df4905bd0b2acd045224491cf.
52. Clark DJ, Kolias AG, Corteen EA, Ingham SC, Piercy J, Crick SJ, et al. Community consultation in emergency neurotrauma research: results from a pre-protocol survey. Acta Neurochirurgica. 2013;155(7):1329-34; discussion 34.
53. Giudice AD, Plaum J, Maloney E, Kasner SE, Le Roux PD, Baren JM. Who will consent to emergency treatment trials for subarachnoid hemorrhage? Academic Emergency Medicine. 2009;16(4):309-15.
54. Goldstein JN, Espinola JA, Fisher J, Pallin DJ, Camargo Jr CA. Public opinion of a stroke clinical trial using exception from informed consent. International Journal of Emergency Medicine. 2010;3(4):385-9.

For peer review only - http://bmjopen.bmj.com/site/about/guidelines.xhtml

55. Newman JT, Smart A, Reese TR, Williams A, Moss M. Surrogate and patient discrepancy regarding consent for critical care research. Critical care medicine. 2012;40(9):2590-4.
56. Scotton WJ, Kolias AG, Ban VS, Crick SJ, Sinha R, Gardner A, et al. Community consultation in emergency neurosurgical research: lessons from a proposed trial for patients with chronic subdural haematomas. British Journal of Neurosurgery. 2013;27(5):590-4.
57. Sims CA, Isserman JA, Holena D, Sundaram LM, Tolstoy N, Greer S, et al. Exception from informed consent for emergency research: Consulting the trauma community. Journal of Trauma and Acute Care Surgery. 2013;74(1):157-66.
58. Snowdon C, Elbourne D, Forsey M, Alfirevic Z. Views of emergency research (VERA): A qualitative study of women and their partners' views of recruitment to trials in severe postpartum haemorrhage. Midwifery. 2012;28(6):800-8.
59. Donovan JL, Rooshenas L, Jepson M, Elliott D, Wade J, Avery K, et al. Optimising recruitment and informed consent in randomised controlled trials: the development and implementation of the Quintet Recruitment Intervention (QRI). Trials. 2016;17(1):283.
60. Elliott D, Husbands S, Hamdy FC, Holmberg L, Donovan JL. Understanding and Improving Recruitment to Randomised Controlled Trials: Qualitative Research Approaches. Eur Urol. 2017.
61. Authority NHR. Applying a proportionate approach to the process of seeking consent - HRA Guidance. 2017.
62. Roberts I, Prieto-Merino D, Shakur H, Chalmers I, Nicholl J. Effect of consent rituals on mortality in emergency care research. Lancet. 2011;377(9771):1071-2.
63. Sahan KM, Channon KM, Choudhury RP, Kharbanda RK, Lee R, Sheehan M. Refining the Enrolment Process in Emergency Medicine Research. Eur J Cardiovasc Med. 2016;4(1):506-10.
64. Hopewell S, McDonald S, Clarke M, Egger M. Grey literature in meta-analyses of randomized trials of health care interventions. The Cochrane database of systematic reviews. 2007(2):MR000010.
65. Ziai H, Zhang R, Chan AW, Persaud N. Search for unpublished data by systematic reviewers: an audit. BMJ open. 2017;7(10):e017737.
66. Tam VC, Hotte SJ. Consistency of phase III clinical trial abstracts presented at an annual meeting of the American Society of Clinical Oncology compared with their subsequent full-text publications. J Clin Oncol. 2008;26(13):2205-11.
67. Toma M, McAlister FA, Bialy L, Adams D, Vandermeer B, Armstrong PW. Transition from meeting abstract to full-length journal article for randomized controlled trials. JAMA. 2006;295(11):1281-7.

For peer review only - http://bmjopen.bmj.com/site/about/guidelines.xhtml

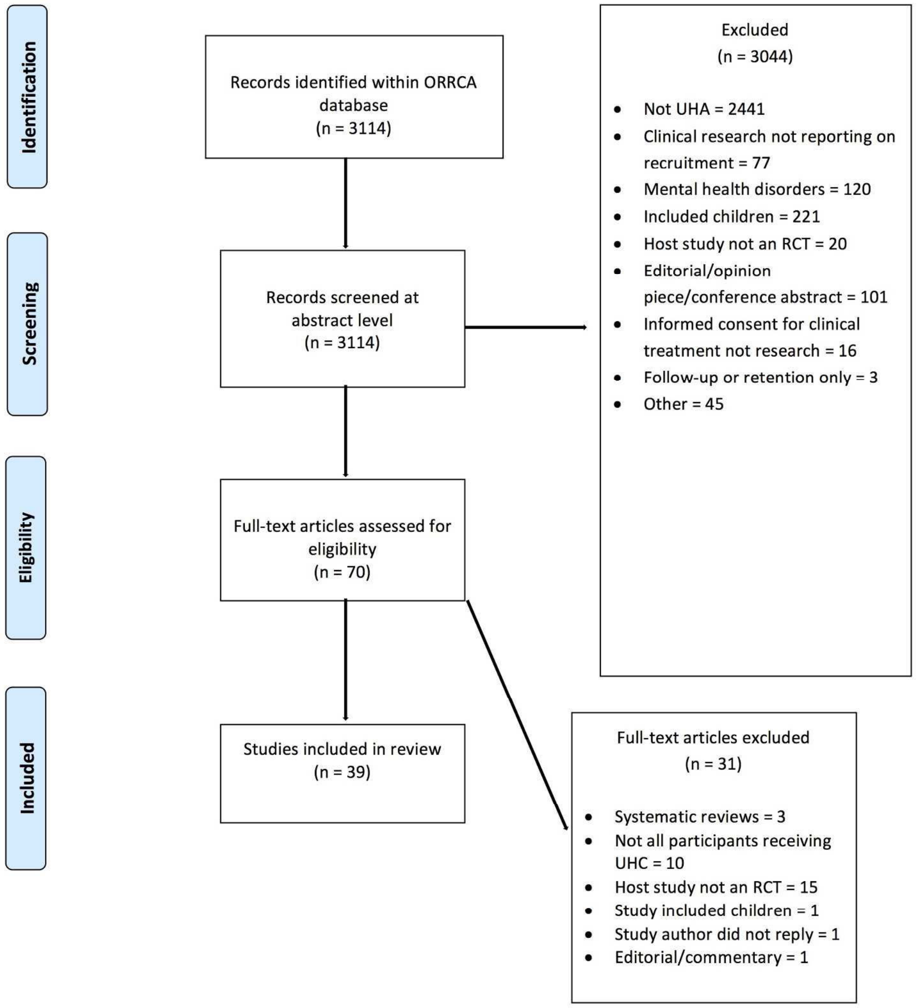

Figure 1: Study selection PRISMA flow diagram

208x242mm (300 x 300 DPI)

	#	Checklist item	Reported on page #
**TITLE**			
Title	1	Identify the report as a systematic review, meta-analysis, or both.	1
**ABSTRACT**			
Structured summary	2	Provide a structured summary including, as applicable: background; objectives; data sources; study eligibility criteria, participants, and interventions; study appraisal and synthesis methods; results; limitations; conclusions and implications of key findings; systematic review registration number.	2
**INTRODUCTION**			
Rationale	3	Describe the rationale for the review in the context of what is already known.	3
Objectives	4	Provide an explicit statement of questions being addressed with reference to participants, interventions, comparisons, outcomes, and study design (PICOS).	5
**METHODS**			
Protocol and registration	5	Indicate if a review protocol exists, if and where it can be accessed (e.g., Web address), and, if available, provide registration information including registration number.	N/A
Eligibility criteria	6	Specify study characteristics (e.g., PICOS, length of follow-up) and report characteristics (e.g., years considered, language, publication status) used as criteria for eligibility, giving rationale.	5-7
Information sources	7	Describe all information sources (e.g., databases with dates of coverage, contact with study authors to identify additional studies) in the search and date last searched.	4-5
Search	8	Present full electronic search strategy for at least one database, including any limits used, such that it could be repeated.	4-5
Study selection	9	State the process for selecting studies (i.e., screening, eligibility, included in systematic review, and, if applicable, included in the meta-analysis).	5-6

For peer review only

Data collection process	10	Describe method of data extraction from reports (e.g., piloted forms, independently, in duplicate) and any processes for obtaining and confirming data from investigators.	7
Data items	11	List and define all variables for which data were sought (e.g., PICOS, funding sources) and any assumptions and simplifications made.	7
Risk of bias in individual studies	12	Describe methods used for assessing risk of bias of individual studies (including specification of whether this was done at the study or outcome level), and how this information is to be used in any data synthesis.	N/A
Summary measures	13	State the principal summary measures (e.g., risk ratio, difference in means).	N/A
Synthesis of results	14	Describe the methods of handling data and combining results of studies, if done, including measures of consistency (e.g., I^2) for each meta-analysis.	N/A

Section/topic	#	Checklist item	Reported on page #
Risk of bias across studies	15	Specify any assessment of risk of bias that may affect the cumulative evidence (e.g., publication bias, selective reporting within studies).	N/A
Additional analyses	16	Describe methods of additional analyses (e.g., sensitivity or subgroup analyses, meta-regression), if done, indicating which were pre-specified.	N/A
**RESULTS**			
Study selection	17	Give numbers of studies screened, assessed for eligibility, and included in the review, with reasons for exclusions at each stage, ideally with a flow diagram.	7
Study characteristics	18	For each study, present characteristics for which data were extracted (e.g., study size, PICOS, follow-up period) and provide the citations.	8-10
Risk of bias within studies	19	Present data on risk of bias of each study and, if available, any outcome level assessment (see item 12).	N/A
Results of individual studies	20	For all outcomes considered (benefits or harms), present, for each study: (a) simple summary data for each intervention group (b) effect estimates and confidence intervals, ideally with a forest plot.	8-10

For peer review only

Synthesis of results	21	Present results of each meta-analysis done, including confidence intervals and measures of consistency.	N/A
Risk of bias across studies	22	Present results of any assessment of risk of bias across studies (see Item 15).	N/A
Additional analysis	23	Give results of additional analyses, if done (e.g., sensitivity or subgroup analyses, meta-regression [see Item 16]).	N/A
**DISCUSSION**			
Summary of evidence	24	Summarize the main findings including the strength of evidence for each main outcome; consider their relevance to key groups (e.g., healthcare providers, users, and policy makers).	10
Limitations	25	Discuss limitations at study and outcome level (e.g., risk of bias), and at review-level (e.g., incomplete retrieval of identified research, reporting bias).	11-12
Conclusions	26	Provide a general interpretation of the results in the context of other evidence, and implications for future research.	13
**FUNDING**			
Funding	27	Describe sources of funding for the systematic review and other support (e.g., supply of data); role of funders for the systematic review.	13-14

From: Moher D, Liberati A, Tetzlaff J, Altman DG, The PRISMA Group (2009). Preferred Reporting Items for Systematic Reviews and Meta-Analyses: The PRISMA Statement. PLoS Med 6(7): e1000097. doi:10.1371/journal.pmed1000097 N/A= not applicable

For more information, visit: **www.prisma-statement.org**.

For peer review only

For peer review only
